# Borylated Cymantrenes and Tromancenium Salts with Unusual Reactivity

**DOI:** 10.1021/acs.organomet.2c00179

**Published:** 2022-05-13

**Authors:** Reinhard Thaler, Holger Kopacka, Klaus Wurst, Thomas Müller, Dennis F. Dinu, Klaus R. Liedl, Florian R. Neururer, Stephan Hohloch, Benno Bildstein

**Affiliations:** † Institute of General, Inorganic and Theoretical Chemistry, Center for Chemistry and Biomedicine, University of Innsbruck, Innrain 80-82, 6020 Innsbruck, Austria; ‡ Institute of Organic Chemistry, Center for Chemistry and Biomedicine, University of Innsbruck, Innrain 80-82, 6020 Innsbruck, Austria

## Abstract

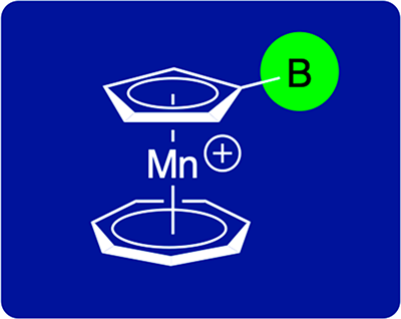

In continuation of our study of the chemistry of cationic (cycloheptatrienyl)(cyclopentadienyl)manganese(I) sandwich complexes, so-called “tromancenium” salts, we report here on their boron-substituted derivatives focusing on useful boron-mediated synthetic applications. Transmetalation of lithiated tricarbonyl(cyclopentadienyl)manganese (“cymantrene”) with boric or diboronic esters affords monoborylated cymantrenes that are converted by advanced high-power LED photosynthesis followed by oxidation with tritylium to their 8-boron-substituted tromancenium complexes. These new functionalized tromancenium salts are fully characterized by ^1^H/^11^B/^13^C/^19^F/^55^Mn NMR, IR, UV–vis, HRMS spectroscopy, single-crystal structure analysis (XRD) and cyclic voltammetry (CV). IR spectra were thoroughly analyzed by density functional theory (DFT) on the harmonic approximation in qualitative agreement of calculated vibrations with experimental values. Uncommon chemical reactivity of these borylated tromancenium salts is observed, due to the strongly electron-withdrawing cationic tromancenium moiety. No Suzuki-type cross-coupling reactions proved so far achievable, but unusual copper-promoted amination with sodium azide under microwave irradiation is possible. Diazoniation of aminotromancenium affords an extremely reactive dicationic tromanceniumdiazonium salt, which is too labile for standard Sandmeyer reactions, in contrast to analogous chemistry of cobaltocenium salts. Overall, borylated tromancenium salts display unexpected and intriguing chemical properties with the potential for novel synthetic applications in future work.

## Introduction

Cationic η^7^-cyclohetatrienyl η^5^-cyclopentadienyl manganese sandwich complexes, commonly called “tromancenium”, are an interesting and neglected class of 18-valence-electron, air-stable, polar metallocenes that have only recently been fully characterized, enabled by an innovative photochemical synthetic approach using advanced high-power LED light sources.^1^ Electronically, these tromancenium salts may be viewed as typical heteroleptic metallocenes containing manganese in the oxidation state +1 coordinated to 6π-Hückel-aromatic cyclopentadienide C_5_H_5_^–^ and tropylium C_7_H_7_^+^ ligands.

In this contribution, we aim to develop this chemistry further with a focus on their boron-substituted functionalized derivatives with potentially widespread useful applications in synthesis, thereby allowing expansion of the chemical space of tromancenium complexes. Due to the air-stability and ionicity of tromancenium salts, they are also soluble in water, a desirable feature for potential applications in green chemistry or medicinal chemistry. Tromancenium salts in general display quasi-reversible one-electron oxidation and reduction,^1^ a desirable feature for potential applications in electrochemistry, redox sensing, and redox catalysis. Given these advantageous properties, we believe it is worthy to develop this chemistry further.

## Results and Discussion

### Synthesis and Reactivity

**Scheme 1:**
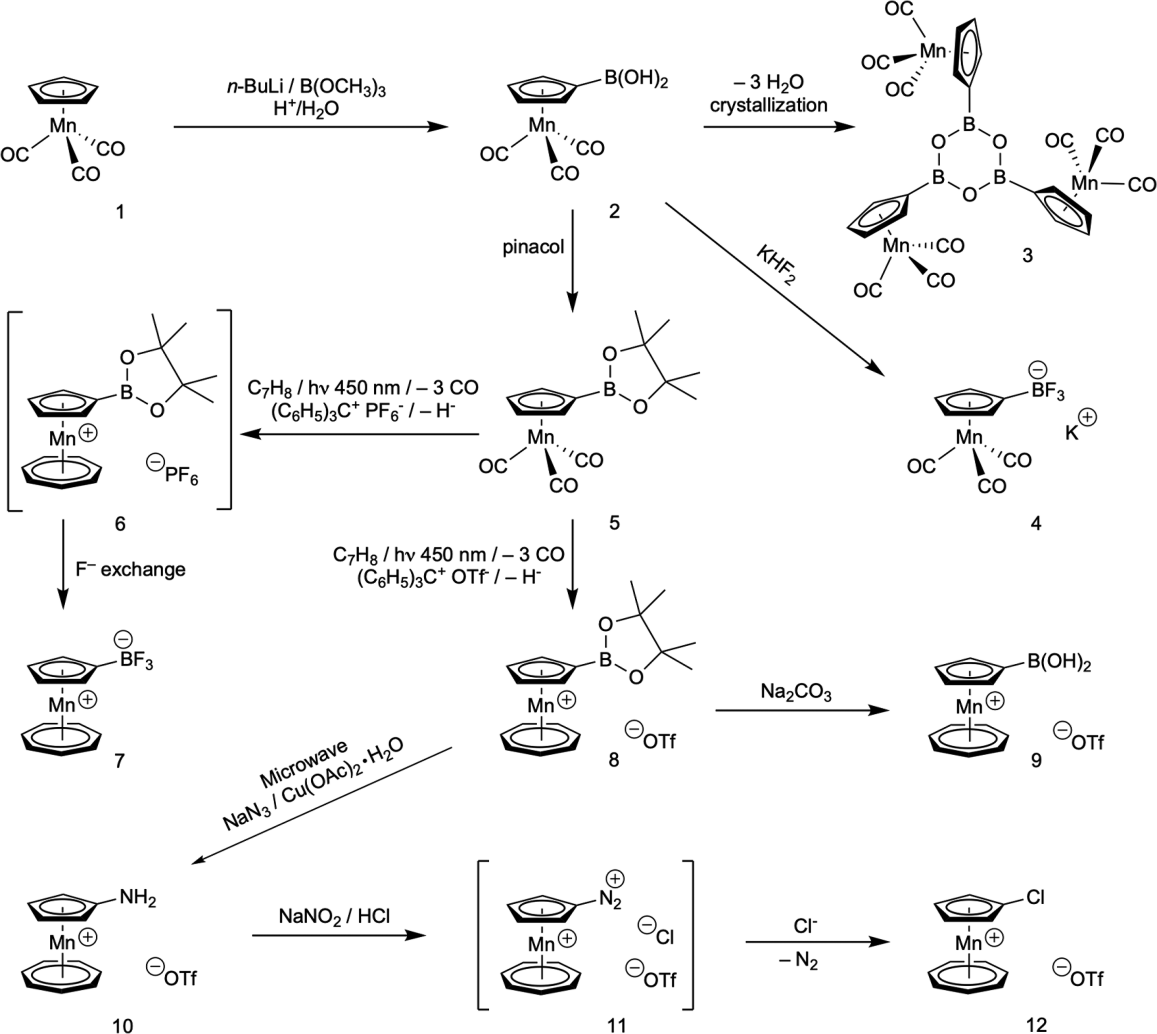
Synthesis of Compounds **2**–**12**

In our previous paper on tromancenium chemistry,^1^ we have seen that the most convenient way to functionalize tromancenium salts consists of the introduction of the functional group (FG) at an early stage of the synthetic sequence at the Cp ring of cymantrene, (CO)_3_MnCp, the key starting material for this chemistry. Subsequent photochemical substitution of all three CO ligands with cycloheptatriene under blue light irradiation (450 nm) with high-intensity LED light sources (370 W) affords air-sensitive (η^6^-C_7_H_8_)(η^5^-C_5_H_4_-FG)Mn intermediates that are oxidized with tritylium salts by the removal of a hydride from the η^6^-C_7_H_8_ ligand to yield the targeted air-stable tromancenium salts. Following this route, 8-substituted tromancenium complexes were readily accessible. Because derivatization of cymantrene by standard organometallic chemistry is very well developed,^2^ either by metalation and nucleophilic substitution with electrophiles or by electrophilic aromatic substitution, a wide range of functional groups is in principle possible. However, the following photochemical substitution of the CO ligands by cycloheptatriene is the critical step in this synthetic protocol—not all functional groups are compatible as we will see in the following (Scheme 1).

Metalation of cymantrene (**1**) by *n*-butyl lithium under standard conditions (THF, −78 °C) followed by reaction with trimethylborate and aqueous workup afforded cymantrenyl boronic acid (**2**) in a satisfying yield of 87% as yellow air-stable compound with spectroscopic properties fully in line with its structure (vide infra). Interestingly, crystallization of **2** aiming at growing suitable single crystals for XRD analysis revealed that condensation to its cyclic anhydride [(CO)_3_Mn(C_5_H_4_)]_3_B_3_O_3_ (**3**) had occurred. We note that **2** has been already synthesized about 50 years ago by Nesmeyanov^3^ and characterized by proton NMR,^4^ but no further information is available in the literature.

In general, organic boron derivatives of the type boronic acid RB(OH)_2_, boronic ester RB(OR′)_2_ and trifluoridoborate RBF_3_^–^ are very useful synthons for carbon–carbon and carbon–nitrogen Suzuki cross-coupling reactions; therefore **2** was converted with KHF_2_ to its trifluoridoborate **4** in 60% yield and with pinacol to its ester **5** in 86% yield. We note that **5** has been prepared before by an iridium-catalyzed C–H activation and borylation of cymantrene,^5^ but our route is simpler and avoids costly iridium. Having these three boronic derivatives in hand, we next tested their compatibility with the rather harsh photochemical reaction conditions^1^ (450 nm, 370 W) of the substitution of their CO ligands with cycloheptatriene. As we suspected from our earlier experience with other substituted cymantrenes,^1^ protic or highly polar substituents should be avoided because of either complete photochemical degradation or a rather poor isolated yield after workup. This proved to be the case also with **2** and **4** as substrates and no productive outcome of the reaction was observed. Contrary, pinacol ester **5** afforded the desired tromanceniumylboronic acid pinacol esters **6** and **8**.

**Scheme 2:**
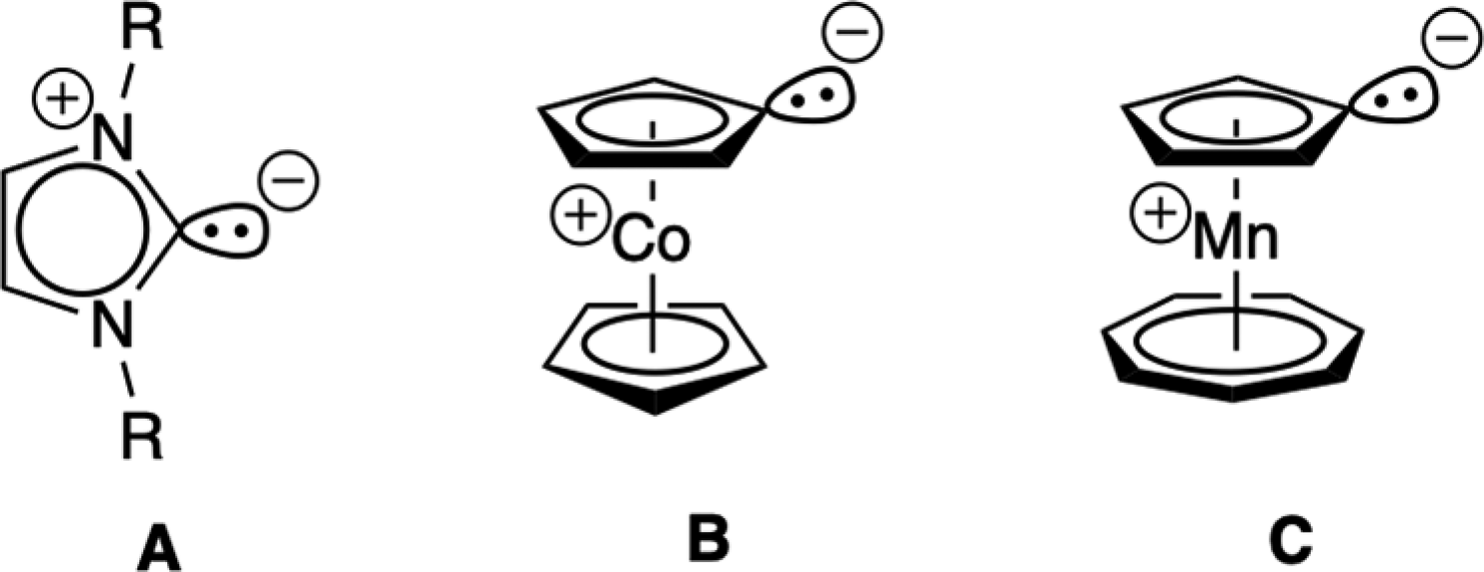
Comparison of Polar Resonance Structures of N-Heterocyclic Carbenes (A), Cobaltocenylidene (B), and Tromancenylidene (C)

Interestingly, tromanceniumylboronic ester (**6**) containing hexafluoridophosphate as counterion proved unstable in solution and reacted within 18 h to mesoionic tromanceniumyltrifluoridoborate (**7**), indicating high thermodynamic stability of this red zwitterion. Mechanistically, we assume that this unusual fluoride abstraction from an otherwise “inert” PF_6_ anion is driven by the formation of strong boron-fluoride bonds, facilitated by the electron-deficient 6-valence-electron boron, and by the high oxophilicity of phosphorus under formation of a five-coordinated heteroleptic pinacolato/fluorido anion [(C_6_H_12_O_2_)PF_3_]^−^. From an organometallic chemist’s viewpoint, **7** is an interesting zwitterionic species containing an interesting novel metallocarbene ligand. In analogy to “cobaltocenylidene” gold complex^6^ [(C_5_H_5_)Co(C_5_H_4_)]Au(CN)_3_, **7** may be viewed as a BF_3_ complex of the analogous, mesoionic metallocenocarbene “tromancenylidene” **C** with a similar electronic structure (Scheme 2). With this analogy in mind, we attempted a carbene transfer reaction of **7** with AgF, aiming at a [(tromancenylidene)_2_Ag]BF_4_ complex that would be a very useful synthon for other tromancenylidene metal complexes, similarly as silver NHC complexes that are preferred carbene precursors for other NHC metal complexes.^7^ Unfortunately, no such reactivity proved possible, even under forcing microwave irradiation, indicating very strong tromancenylidene-boron bonding. Similar earlier attempts by Arduengo^8a^ to transfer an imidazolylidene from an NHC-BF_3_ adduct to mercury by reaction with HgF_2_ were also met with failure.

To finally get access to tromanceniumyl pinacol boronic ester (**8**), we therefore had to exchange the noninnocent hexafluoridophosphate by a more suitable stable anion. This is most easily accomplished using tritylium triflate^9^ instead of tritylium hexafluorophosphate in the hydride removal/oxidation step after photolysis of **5** with cycloheptatriene. In this manner, **8** was synthesized in a satisfying yield of 80%. Subsequent mild hydrolysis with aqueous sodium bicarbonate afforded the parent tromanceniumylboronic acid triflate (**9**) also in 80% yield as an air-stable, purple salt that crystallizes (vide infra) without condensation to its anhydride, in contrast to cymantrenylboronic acid (**2**) (vide supra).

With three new tromanceniumylboronic derivatives **7**, **8**, and **9** in hand, it was obviously of interest to use them in Suzuki–Miyaura cross-coupling reactions^10^ to expand the chemical space of tromancenium salts. Toward this goal, we performed Suzuki–Miyaura reactions with bromobenzene as electrophilic test substrate, employing various standard Pd, Ni, or Cu catalysts under a variety of experimental conditions, including also dual photoredox catalysis.^11^ Unfortunately, in no case successful cross-coupling under formation of 8-phenyl-tromancenium triflate was observed, in part explainable by the strongly electron-withdrawing and deactivating cationic tromancenium moiety. Another useful reaction of boronic derivatives is the copper-catalyzed Chan–Evans–Lam amination of boronic acids or esters to primary, secondary, or tertiary aryl amines.^12^ Synthetically, primary aryl amines are clearly the most valuable compounds for further N-functionalization; therefore we focused our efforts toward 8-aminotromancenium triflate (**10**) as target. We have synthesized 8-aminotromancenium hexafluoridophosphate before by direct photochemical synthesis starting from amino cymantrene, but due to the photolabile amino substituent the isolated yield was less than 10%,^1^ thereby preventing further study of its reactivity. Applying standard Chan–Evans–Lam reaction conditions on boronic derivatives **7**, **8**, and **9** proved unfortunately unsuccessful, but interestingly, reaction of pinacol ester **8** with sodium azide under microwave irradiation gave 8-aminotromancenium triflate (**10**) in a satisfying yield of 72%. The mechanism of this unusual reaction is unclear at the moment, but besides carbon–nitrogen coupling a reduction of azide to amino has to occur, most likely by the protic solvent ethanol.

Having now aminotromancenium **10** available in sufficient quantity, we studied next its diazoniation to dicationic tromanceniumdiazonium salt (**11**), based on our experience with nucleophilic or radical substitution reactions of cobaltoceniumdiazonium bis(hexafluoridophosphate).^13^ Conversion of aminotromancenium triflate (**10**) with sodium nitrite in aqueous hydrochloric acid or with aqueous hexafluorophosphoric acid afforded extremely reactive diazonium species. All our attempts to characterize or isolate this compound by precipitation as its bis(hexafluoridophosphate) salt met without success, in contrast to its cobaltocenium analogue.^13^ Furthermore, attempts to react tromanceniumdiazonium in situ with azide and iodide as the most reactive nucleophiles in dediazoniation reactions^14^ did neither afford azidotromancenium nor iodotromancenium. Instead, only chlorotromancenium triflate (**12**) in admixture with parent unsubstituted tromancenium was obtained from **11** in <50% yield, obviously formed by reaction with the chloride counteranion. With tromanceniumdiazonium bis(hexafluoridophosphate) generated in situ by NaNO_2_/HPF_6_ followed by reaction with NaN_3_ or KI, only complete degradation to unsubstituted tromancenium was observed. This is in stark contrast to common organic aryldiazonium chemistry^14^ where dediazoniation by the poor nucleophile chloride is only possible under copper-catalyzed Sandmeyer conditions, but not without copper catalysts. Overall, tromanceniumdiazonium salts are very labile species exhibiting surprising chemoselectivity, in contrast to cobaltoceniumdiazonium salts^13^ that have a more common and synthetically more useful reactivity in nucleophilic or radical substitution reactions.

### Structural, Spectroscopic, and Electrochemical Properties

**Figure 1:**
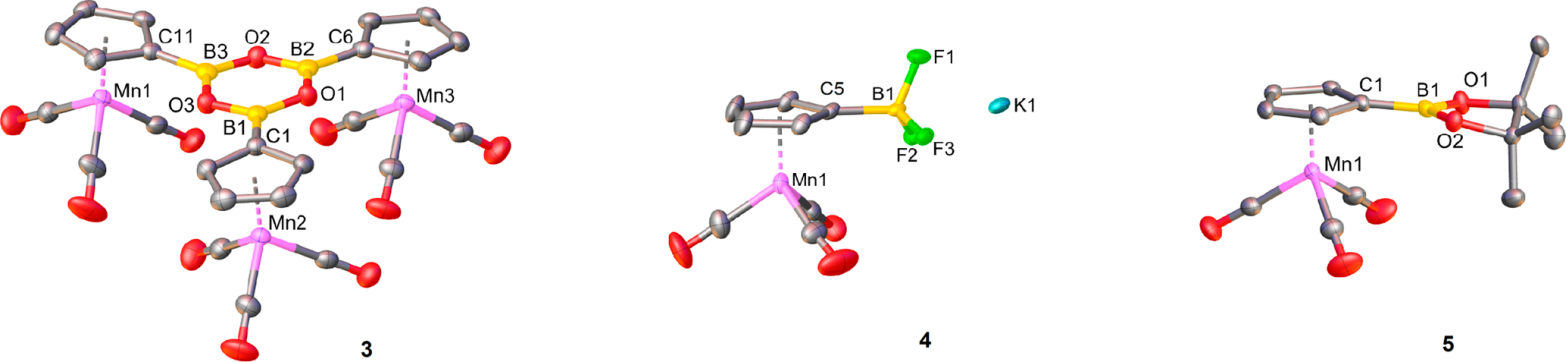
Molecular structure of cymantrene derivatives **3**, **4**, and **5**. Hydrogen atoms are omitted for clarity. Selected bond lengths (Å): **3**: Mn1–C_Cp_(avg) = 2.142, Mn2–C_Cp_(avg) = 2.140, Mn3–C_Cp_(avg) = 2.141, C1–B1 = 1.545(2), C6–B2 = 1.537(3), C11–B3 = 1.532(3), B1–O1 = 1.376(2), B1–O3 = 1.374(2), B2–O1 = 1.384(2), B2–O2 = 1.374(2), B3–O2 = 1.379(2), B3–O3 = 1.379(2); **4**: Mn1–C_Cp_(avg) = 2.147, C5–B1 = 1.594(2), B1–F1 = 1.413(2), B1–F2 = 1.411(2), B1–F3 = 1.429(2); **5**: Mn–C_Cp_(avg) = 2.143, C1–B1 = 1.547(2), B1–O1 = 1.368(2), B1–O2 = 1.364(2).

**Figure 2:**
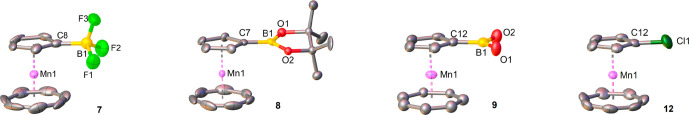
Molecular structure of tromancenium salts **7**, **8**, **9**, and **12**. Counteranions triflates and hydrogen atoms are omitted for clarity, except for zwitterionic **7**. Selected bond lengths (Å): **7**: Mn1–C_Cp_(avg) = 2.121, Mn1–C_Cht_(avg) = 2.119, C8–B1 = 1.619(11), B1–F1 = 1.421(10), B1–F2 = 1.414(9), B1–F3 = 1.417(10); **8**: Mn1–C_Cp_(avg) = 2.121, Mn1–C_Cht_(avg) = 2.120, C7–B1 = 1.542(13), B1–O1:1.339(12), B1–O2 = 1.401(12); **9**: Mn1–C_Cp_(avg) = 2.115, Mn1–C_Cht_(avg) = 2.122, C12–B1 = 1.566(5), B1–O1 = 1.352(5), B1–O2 = 1.350(5); **12**: Mn1–C_Cp_(avg) = 2.110, Mn1–C_Cht_(avg) = 2.133, C12–Cl = 1.713(3).

Single crystal analyses are available for cymantrene derivatives **3**,^15^**4**, **5** (Figure 1) and for tromancenium salts **7**, **8**, **9**, and **12** (Figure 2). The solid-state structure of an aminotromancenium **10′** containing hexafluoridophosphate as counterion is already included in our previous paper.^1^ In general, the molecular structures of these new cymantrenes and tromancenium salts prove unambiguously their chemical identity, displaying regular half-sandwich (**3**, **4**, **5**) or sandwich (**7**, **8**, **9**, **12**) structures with bond distances and angles in the expected range; detailed relevant metrics are given in the footnotes of Figures 1 and 2. In the tromancenium series, molecular structures of **7**, **8**, **9**, and **12** are very similar to those of other tromancenium derivatives reported in our previous paper,^1^ differing only in their varying substituents. A general structural feature of all tromancenium salts is the similarity of their manganese–carbon bond distances (Mn–C_averaged_ = 2.12 Å) to carbons of the cyclopentadienyl (Cp) and cycloheptatrienyl (Cht) rings, thereby the larger 7-membered Cht ligand is pulled closer to the central manganese atom with corresponding shorter Mn–Cht_centroid_ distances (approximately 1.3 Å) in comparison to those of the smaller 5-membered Cp ligand (approximately 1.7 Å). Similar structural properties have been reported for Cht/Cp sandwich metal complexes of early transition metals.^16^ The C(8)–B(1) bond distance of tromanceniumyltrifluoridoborate (“tromancenylidene”-BF_3_ adduct) (**7**) is 1.619(11) Å (Figure 2), very similar to carbene carbon–boron bond lengths of imidazolylidene-BF_3_ complexes,^8a,8b^ indicating at first sight a comparable donor strength of tromancenylidene **C** and standard Arduengo carbenes **A** (Scheme 2).

**Figure 3:**
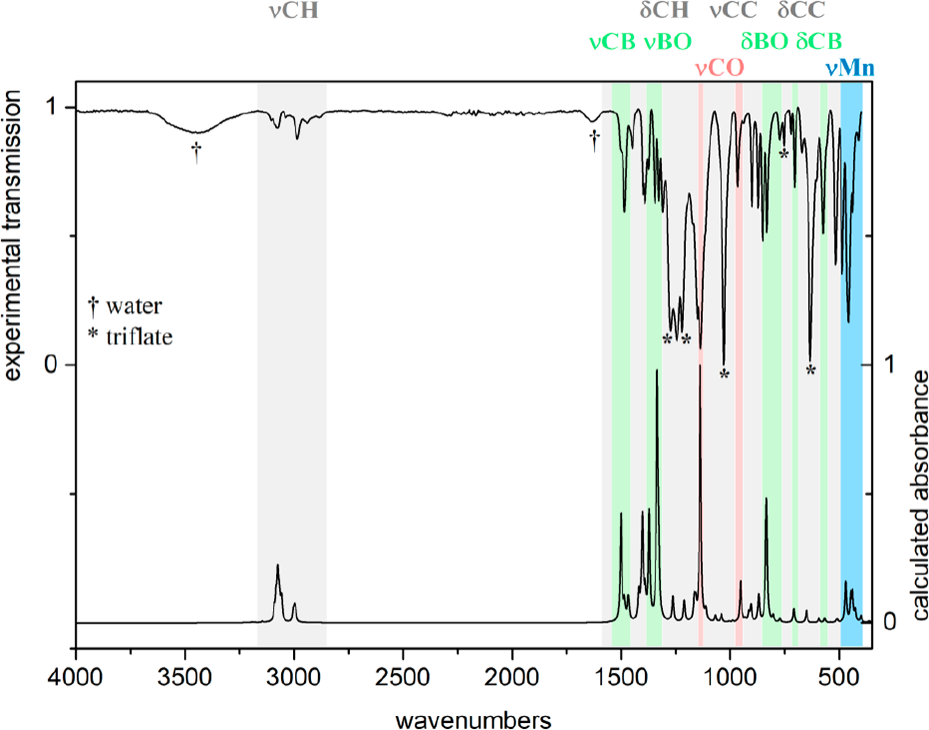
Experimental (top) and calculated (bottom, without triflate anion) IR spectrum of tromanceniumylboronic acid pinacol ester triflate (**8**).

High-resolution mass spectra show the monoisotopic most abundant signals of the molecular ions (**2**, **4**, **5**) or of the cations (**7**, **8**, **9**, **12**) in excellent agreement with theoretical values (see Supporting Information). UV–vis spectra of yellow cymantrene derivatives **2**, **4**, and **5** are rather simple, but those of tromancenium salts **7**, **8**, **9**, and **12** display a broad charge-transfer absorption at 550–580 nm, in line with their red color. IR spectroscopy of cymantrenes **2**, **4**, and **5** as well as those of tromancenium salts **7**, **8**, **9**, and **12** were measured in the ATR mode and fully analyzed by DFT calculations in the gas phase approximating vibration modes as harmonic oscillations (see Supporting Information). This allowed a detailed assignment of all relevant absorptions, a representative example of tromanceniumylboronic acid pinacol ester triflate (**8**) is depicted in Figure 3. The wavenumbers of experimental and calculated IR bands are in good qualitative agreement, whereas the relative intensities are not so well reproduced, explainable by the ATR-measurement of the experimental data in the bulk phase and by the DFT calculation in the gas phase. In general, C–C vibrations of the cyclopentadienyl/cyclohepatrienyl ligands and of the pinacol ester show higher intensities in the bulk compared to the gas phase.

Multinuclear NMR spectroscopic characterization includes ^1^H, ^11^B, ^13^C, ^19^F and most notably also ^55^Mn NMR data (compare Supporting Information). All these new cymantrenes and tromancenium complexes contain monosubstituted Cp ligands with corresponding simple signal patterns in their ^1^H (two pseudotriplets) and ^13^C (two signals and one mostly undetected weak signal for the ipso-carbon) spectra in the usually observed spectral region for metallocenes. The singlets of the unsubstituted cycloheptatrienyl ligand of tromancenium salts **7**, **8**, **9**, and **12** are observed in a narrow range of 6.7 to 6.92 ppm (^1^H) and 97.1 to 99.2 ppm (^13^C), respectively. ^1^H/^13^C chemical shifts of neutral cymantrene derivatives **2**, **4**, and **5** are at higher field in comparison to those of cationic tromancenium complexes **7**, **8**, **9**, and **12**, as expected. ^11^B NMR spectra of **2**, **4**, **5**, **7**, **8** show quadrupolar-broadened signals from −16 to +30 ppm with the most shielded chemical shifts observed for BF_3_ derivatives (**4**, δ(^11^B) = −7 ppm; **7**, δ(^11^B) = −15.7 ppm) that also show resolved quartets due to scalar coupling (**4**, ^1^*J*(^11^B–^19^F) = 48.3 Hz; **7**, ^1^*J*(^11^B–^19^F) = 48.3 Hz). Concomitantly, these couplings are also observed as quartets in a 1:1:1:1 signal intensity ratio in their ^19^F spectra, due to the *I* = 3/2 nuclear spin of ^11^B.

**Figure 4:**
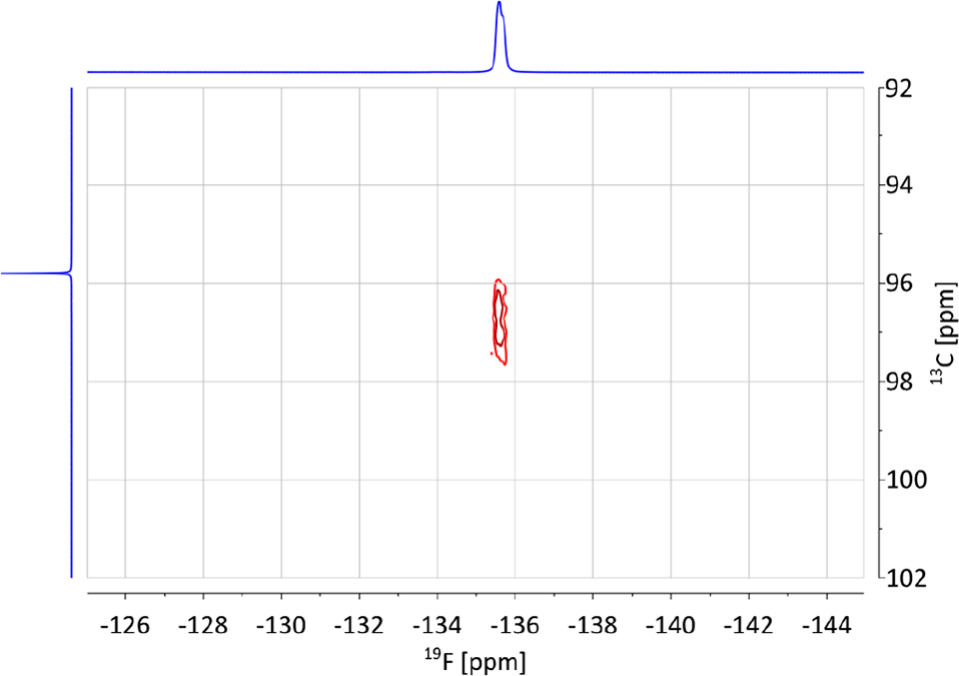
(^19^F/^13^C)-HSQC NMR spectrum of tromancenylidene-BF_3_ adduct **7** in saturated DMSO-*d*_6_ solution.

Of special interest is the ^13^C chemical shift of the quaternary ipso-carbon of tromancenylidene-BF_3_ adduct **7** to evaluate the donor character of this new metallocenocarbene **C** (Scheme 2) in comparison to standard N-heterocyclic carbenes **A** (Scheme 2). This is a challenging task, because the expected signal intensity will be extremely low due to splitting into a 16-line multiplet [quartet (^1^*J*(^13^C–^11^B[*I* = 3/2]) × quartet (^2^*J*(^13^C–^19^F)] in 80% relative intensity (natural abundance of ^11^B = 80.1%), overlaid (if resolved at all) on a 28-line multiplet [septet (^1^*J*(^13^C–^10^B[*I* = 3]) × quartet (^2^*J*(^13^C–^19^F)] in 20% relative intensity (natural abundance of ^10^B = 19.9%). Under standard ^13^C NMR measurement conditions, such a high-multiplicity signal is obviously very difficult to detect. However, for highly concentrated solutions^8b^ or with ^19^F–^13^C decoupling^8a^ the carbene centers of two NHC-BF_3_ adducts have been observed in the range of 157–163 ppm. In our case, a (^19^F/^13^C)-HSQC NMR of a saturated solution of **7** in DMSO-*d*_6_ on a 700 MHz NMR spectrometer gave a distinctive elongated cross-peak (Figure 4) with an appearance explainable as an unresolved quartet due to ^1^*J*(^13^C–^11^B) coupling. Its ^13^C chemical shift of 96.8 ppm is extremely shielded by 65 ppm in comparison to carbene signals of NHC-BF_3_ adducts (157–163 ppm).^8a,8b^ By incidence, the signals of the ipso-carbon (96.8 ppm) and cycloheptatrienyl-carbons (singlet, 95.8 ppm) of **7** are close in value, but clearly no coupling between the BF_3_ group and the remote cycloheptatrienyl ligand is possible or evident. For comparison of tromancenylidene **C** (Figure 2) with metallocenocarbene **B** (Figure 2), the ipso-carbon of cobaltocenylidene-Au(CN)_3_ has been observed at 113.6 ppm.^6^ Hence we may conclude that tromancenylidene **C** is an extraordinarily electron-rich carbene, probably even more nucleophilic than cobaltocenylidene **B** with a calculated Tolman electronic parameter (TEP) of 2037.1 cm^–1^.^6^

**Figure 5:**
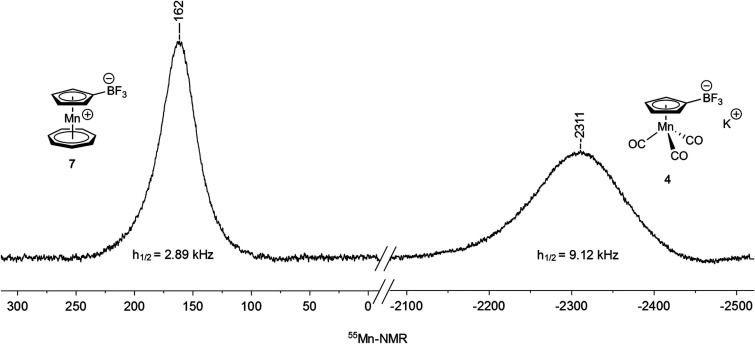
Comparison of ^55^Mn NMR chemical shifts (δ) and peak widths at half height (*h*_1/2_) of tromanceniumyltrifluoridoborate (**7**) (left) and potassium cymantrenyltrifluoridoborate (**4**) (right).

^55^Mn NMR spectroscopy (natural abundance: 100%, receptivity relative to ^1^H: 0.179, *I* = 5/2, *Q* = 0.40 × 10^–28^ m^2^) is in general very rarely applicable^17^ in molecular chemistry, mainly due the high nuclear electric quadrupole moment *Q* preventing observation of signals for nonsymmetric diamagnetic compounds. However, manganese carbonyl derivatives and tromancenium salts have a distorted but sufficiently symmetric pseudo-octahedral ligand sphere at the manganese center that allows detection of signals with peak widths at half-height (*h*_1/2_) in the kHz range.^1,17^^55^Mn NMR spectra of the new cymantrenes **2**, **4**, and **5** and tromancenium salts **7**, **8**, **9**, and **12** (Figure 5, Table 1, for spectra see Supporting Information) show broad signals with larger peak widths *h*_1/2_ for the “less” symmetric cymantrene derivatives in comparison to those of tromancenium complexes with higher local symmetry at the manganese center. Evidently, manganese complexes of similar structure give rise to ^55^Mn chemical shifts in a narrow range (approximately 300 ppm for cymantrenes and also for tromancenium salts) but in well-separated spectral domains [Δ(δ_cymantrenes_ – δ_tromancenium salts_) = 2000 ppm]. As we have observed earlier^1^ and as it is common in general in transition metal NMR spectroscopy, there is no simple correlation of chemical shifts with Hammett substituent parameters within a subclass of compounds. However, the value of 161.6 ppm for tromanceniumyltrifluoridoborate (**7**) is quite low, indicating an unusual electronic structure at manganese, correlating in part with its description as an unusual tromancenylidene-BF_3_ complex (vide supra).

**Table 1:** ^55^Mn Chemical Shifts of Cymantrene Derivatives and of Tromancenium Salts

compound formula (number)	Cp substituent	δ(^55^Mn)^a^	*h*_1/2_^b^	reference
Cymantrene Derivatives				
(CO)_3_MnC_5_H_5_	H	–2225	9.03	(17a), (17c); this work
(CO)_3_MnC_5_H_4_B(OH)_2_ (**2**)	B(OH)_2_	–2140	13.27	this work
K[(CO)_3_MnC_5_H_4_BF_3_] (**4**)	BF_3_^–^	–2311	9.12	this work
(CO)_3_MnC_5_H_4_Bpin (**5**)	pin^c^	–2138	7.76	this work
Tromancenium Salts				
[(C_7_H_7_)Mn(C_5_H_5_)]PF_6_	H	+271	1.20	(1)
[(C_7_H_7_)Mn(C_5_H_4_CH_3_)]PF_6_	CH_3_	+257	1.75	(1)
[(C_7_H_7_)Mn(C_5_H_4_NH_2_)]PF_6_ (**10**′)^d^	NH_2_	+238	3.60	(1)
[(C_7_H_7_)Mn(C_5_H_4_Br)]PF_6_	Br	+368	1.95	(1)
[(C_7_H_7_)Mn(C_5_H_4_C(O)OCH_3_]PF_6_	C(O)OCH_3_	+538	1.85	(1)
(C_7_H_7_)Mn(C_5_H_4_BF_3_) (**7**)	BF_3_^–^	+162	2.89	this work
[(C_7_H_7_)Mn(C_5_H_4_Bpin)]OTf (**8**)	pin^c^	+415	2.92	this work
[(C_7_H_7_)Mn(C_5_H_4_B(OH)_2_)]OTf (**9**)	B(OH)_2_	+367	3.80	this work
[(C_7_H_7_)Mn(C_5_H_4_Cl)]OTf (**12**)	Cl	+369	1.55	this work

a Referenced versus saturated KMnO_4_/D_2_O solution (ppm).

b Peak width at half-height (kHz).

c pin = pinacolate.

d Aminotromancenium (**10**) with PF_6_ counterion.

**Figure 6:**
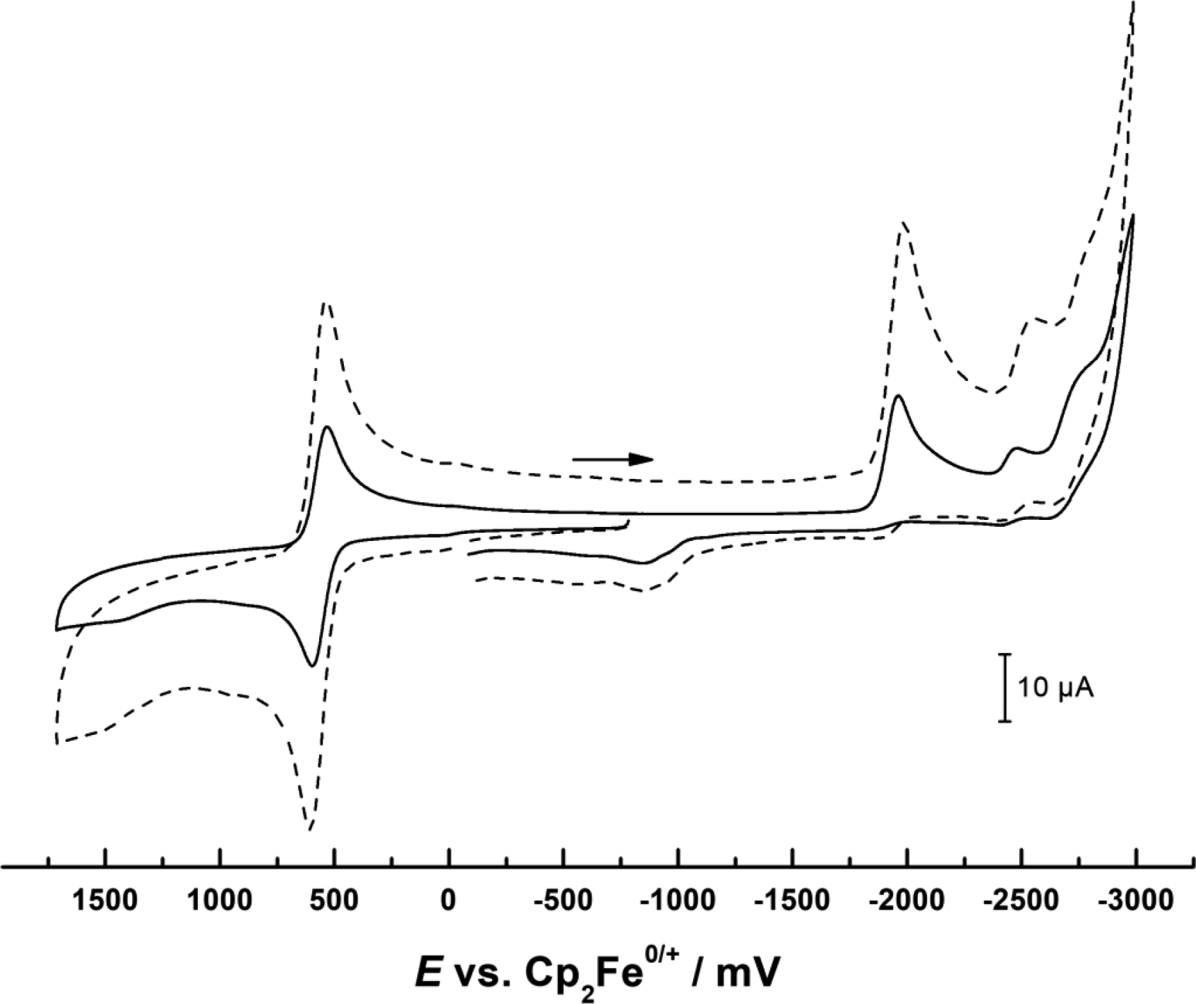
Cyclic voltammogram of tromanceniumyltrifluoridoborate (**7**) in CH_3_CN (0.15 M NBu_4_^–^ PF_6_^–^) on a glassy carbon working electrode at sweep rates of 0.1 (solid line) and 0.5 V s^–1^ (dotted line).

Electrochemical characterization of tromancenium salts **7**, **8**, **9**, and **12** was performed by cyclovoltammetry in acetonitrile as solvent using 0.15 M NBu_4_PF_6_ as the supporting electrolyte. Table 2 summarizes the results and Figure 6 shows a representative cyclovoltammogram of tromanceniumyltrifluoridoborate (**7**). Cyclic voltammetric responses for these tromancenium salts were more or less similar (Table 1, Supporting Information) and comparable to those of other tromancenium complexes in our previous paper.^1^ In general, chemically partially reversible oxidation processes at mild potential were observed, corresponding to the Mn(I)/Mn(II) couple. On the cathodic side, chemically irreversible reductions that are difficult to assign are evident. Overall, redox chemistry of tromancenium salts is more or less well behaved on the anodic side with Mn(I)/Mn(II) redox couples dependent on electron donating or electron withdrawing substituents, whereas reductions give rise to chemical follow-up products of unknown structure.

**Table 2:** Redox Potentials of Tromancenium Salts **7**, **8**, **9**, and **12**^a^

compound #	*E*_ox_^b^	*E*_red,1_^c^	*E*_red,2_^c^
**7**	+0.56	–1.96	–2.48
**8**	+0.83	–1.55	–2.46
**9**	+0.81	–1.58	
**12**	+0.01	–1.40	

a Potentials are given in volts, calibrated against the ferrocene/ferrocenium redox couple. Measurements were conducted in 0.15 M NBu_4_^+^PF_6_^–^ in acetonitrile at sweep rates of 0.1 V s^–1^.

b Half-wave potential of a chemically quasi-reversible process.

c Peak potential of a chemically irreversible process.

## Summary

Cymantrenylboronic acid, (CO)_3_Mn(C_5_H_4_B(OH)_2_), was conveniently synthesized by lithiation of cymantrene followed by reaction with trimethylborate and hydrolysis. Reaction with KHF_2_ gave the trifluoridoborate derivative and transesterification with pinacol afforded cymantrenylboronic acid pinacol ester. These three new borylated cymantrenes might become valuable synthons for Suzuki-type carbon–carbon cross-coupling reactions. Photochemical displacement of all three carbonyl ligands using high-intensity blue light in a LED photoreactor followed by hydride removal/oxidation with tritylium hexafluoridophosphate or triflate enabled access to new cationic cycloheptatrienyl cyclopentadienyl manganese sandwich complexes containing a boronic acid pinacol ester substituent and either hexafluoridophosphate or triflate as counterion. Interestingly, the hexafluoridophosphate salt is unstable in solution and reacted further by fluoride transfer from the anion to the electron-deficient boron substituent of the cation to zwitterionic tromanceniumyltrifluoridoborate, a first and very stable complex of the new electron-rich mesoionic “tromancenylidene” carbene. On the other hand, tromanceniumylboronic pinacolate triflate is a good starting material to get access to tromanceniumylboronic acid triflate by mild hydrolysis. Attempts at using these new borylated tromancenium salts in Suzuki–Miyaura cross-coupling reactions proved unsuccessful so far, even under dual photoredox catalysis. However, tromanceniumylboronic pinacolate triflate showed unusual copper-catalyzed substitution/reduction with sodium azide under microwave irradiation, resulting in aminotromencenium triflate. Diazoniation of this cationic amine with NaNO_2_/HCl afforded an extremely labile intermediate tromanceniumdiazonium dication that degraded in situ to chlorotromancenium triflate by an unusual copper-uncatalyzed intramolecular nucleophilic substitution. Thorough structural and spectroscopic characterization by ^1^H/^11^B/^13^C/^19^F/^55^Mn NMR, IR, UV–vis, HRMS, single crystal structure analysis (XRD) and cyclic voltammetry (CV) was performed. Notably, rarely applicable ^55^Mn NMR proved possible, as well as complete assignment of IR vibrations by DFT calculations.

In summary, tromancenium complexes containing functionalized boron substituents were synthesized from their cymantrene precursors and showed uncommon reactivities, due their cationic, strongly electron-withdrawing and deactivating tromancenium moiety. Further studies are under way to exploit this chemistry toward new synthetic targets and potential catalytic or bioorganometallic applications.

## Experimental Section

### General Procedures

Standard methods and procedures of organometallic synthesis were performed. Starting chemicals were obtained commercially and used as received, except for CpMn(CO)_3_ (cymantrene) (**1**), which was purified by chromatography as described recently.^1^ Tritylium triflate was synthesized as published.^9^^1^H, ^19^F NMR spectra (Bruker Ascent 400 NMR spectrometer), ^11^B, ^13^C, ^55^Mn NMR spectra (Bruker Avance DPX300 NMR spectrometer), and ^19^F/^13^C-HSQC-NMR spectra (700 MHz Bruker Avance 4 Neo spectrometer) were recorded at ambient temperature. Signals were referenced internally against ^1^H/^13^C residual solvent peaks or externally: ^11^B, B(OCH_3_)_3_ neat (Bruker Avance DPX300 NMR spectrometer; for comparison of data to the IUPAC standard BF_3_·Et_2_O + 19.1 ppm has to be added), or BF_3_·Et_2_O (Bruker Avance 4 Neo NMR spectrometer); ^19^F, CCl_3_F, neat; ^55^Mn, KMnO_4_, saturated solution in D_2_O. Mass spectrometric data were measured on a Thermo Finnigan Q Exactive Orbitrap spectrometer, IR spectra were recorded on a Bruker ALPHA IR spectrometer, UV–vis spectra were measured on a PerkinElmer Lambda XLS+ spectrometer, single-crystal X-ray diffraction data were collected on a Bruker D8 Quest diffractometer with graphite-monochromated Mo Kα radiation (λ = 0.71073 Å), and structures were solved by direct methods. Cyclic voltammograms were recorded in an argon filled glovebox, using a BioLogic SP-150 potentiostat with a three-electrode setup (glassy carbon working electrode, platinum wire counter electrode, silver wire pseudoreference) and NBu_4_^+^PF_6_^–^ as supporting electrolyte (0.15 M). All potentials were calibrated internally to the ferrocene/ferrocenium redox couple. Due to significant electrode passivation, repeated electrode polishing was necessary in order to obtain reproducible results. Photochemical synthesis was performed with blue light (450 nm) irradiation at 370 W power in a high-intensity LED photoreactor^18^ described in detail in our previous publication on tromancenium chemistry.^1^ For microwave-assisted-synthesis a CEM MARS 6 Microwave Digestion System was used.

### Density Functional Theory Calculations

The calculated infrared spectra are for single molecules in vacuo. The calculations rely on the harmonic approximation, where the overall molecular vibration is simplified as uncoupled vibrational modes each described by a parabolic function. This model generally overestimates vibrational frequencies compared to experiment, which is why a scaling factor was introduced for each computed spectrum separately to better fit the experimental spectra. The scaling factors (s.f.) are given in the figures of the IR spectra. For the molecules **2**, **4**, **5**, **7**, **8**, **9**, and **12**, the harmonic frequencies have been computed with the Gaussian16 software package,^19^ where the Hessian is constructed by numerical second derivatives of the electronic energy with respect to Cartesian displacements. The electronic energy has been computed with density functional theory, using the B3LYP functional and the 6-311G(d,p) basis set. Counterions have not been considered explicitly. The triflate ion was computed separately to assign its characteristic bands in the spectra of **8**, **9**, and **12**.

#### Cymantrenylboronic acid (**2**)

A round-bottom Schlenk flask was charged with 0.100 g of cymantrene (**1**) (0.490 mmol, 1 equiv), 18 mL of dry THF, and cooled to −78°, then 235.2 μL (0.588 mmol, 1.2 equiv) of a 2.5 M *n*-BuLi solution in hexane was added cautiously while the reaction mixture was vigorously stirred. As soon as the temperature reached −60 °C, 0.1 mL (0.895 mmol, 1.83 equiv) of trimethyl borate was added and the reaction was allowed to warm up to room temperature, whereby a color change from light red to orange was observed. Excessive *n*-BuLi as well as lithium methanolate were quenched with 10 mL of water. For hydrolysis, 10 mL of a 1 M HCl solution was added to the mixture and stirring was continued for additional 120 min, whereby a color change from orange to light yellow was observed. The solvents were removed in vacuo and the product was extracted with diethyl ether and filtered off. Diethyl ether was removed in vacuo affording cymantrenylboronic acid as yellow solid in 87.0% yield (0.105 g, 0.426 mmol). **2** is soluble in diethyl ether, dichloromethane, acetonitrile, acetone, and water. mp 120.7 °C cond. ^1^H NMR (400 MHz, CD_3_CN, ppm) δ = 4.96 (unresolved pseudo-t, 2H, C3/C4 of Cp), 5.17 (unresolved pseudo-t, 2H, C2/C5 of Cp), 5.89 (s, 2H, OH). ^13^C NMR (75 MHz, CD_3_CN, ppm) δ = 84.3 (ipso-carbon of Cp), 86.4 (C3/C4 of Cp), 92.2 (C2/C5 of Cp), 226.0 (carbonyl-carbon). ^55^Mn NMR (74 MHz, CD_3_CN, ppm) δ = −2140. ^11^B NMR (96 MHz, CD_3_CN, ppm) δ = 10.2. IR (ATR, cm^–1^) 3115 (ν_*C*–*H*_), 2011, 1901 (ν_*C*≡*O*_), 1414 (ν_*C*–*H*_), 1377, 1351, 1206 (ν_*C*=*C*_), 1318 (ν_*B*–*O*_), 1061, 1041 (δ_*ip*,*C*–*H*_), 928, 901 (δ_*ip*,*O*–*H*_), 843 (δ_*C*=*C*_), 744, 734 (δ_*oop*,*C*–*H*_), 661 (δ_*C*–*B*_), 622 (ν_*Mn*_), 529 (scaffold), 485 (δ_*oop*, *O*–*H*_). HRMS (ESI neg, *m*/*z*) 246.9614 ([M – H^+^]^−^), calc. for C_8_H_5_O_5_MnB: 246.9616. UV–vis (CH_3_CN, [nm]) λ_max_ = 323 nm. Single crystals were obtained from acetone at room temperature whereby condensation led to cymantrenylboronic acid anhydride (**3**).

#### Potassium cymantrenyltrifluoridoborate (**4**)

A Teflon round-bottom flask was charged with 0.0305 g (1.0 equiv) of cymantrenylboronic acid (**2**), 10 mL of acetone–water (1:1) and potassium hydrogen difluoride (0.022 g, 6.4 equiv). The solution was stirred overnight, then solvents were removed in vacuo, and the product was extracted with acetone and filtered off. Removal of acetone resulted in 60% yield (0.0227 g, 0.0732 mmol). **4** is soluble in acetonitrile, acetone, and water. mp 319.7 °C dec. ^1^H NMR (400 MHz, CD_3_CN, ppm) δ = 4.69 (s, 4H, C1–C4 of Cp). ^13^C NMR (75 MHz, CD_3_CN, ppm) δ = 84.0 (C3/C4 of Cp), 88.0 (C2/C5 of Cp), 228.3 (*ipso*-carbon of Cp). ^55^Mn NMR (74 MHz, CD_3_CN, ppm) δ = −2311. ^11^B NMR (96 MHz, CD_3_CN, ppm) δ = −6.92 (q, *J* = 48.3, 47.9 Hz). ^19^F NMR (376 MHz, CD_3_CN, ppm) δ = −138.74 (q, *J* = 48.2 Hz). IR (ATR, cm^–1^) 3101 (ν_*C*–*H*_), 2005, 1893 (ν_*C*≡*O*_), 1461, 1379, 1232 (ν_*C*=*C*_), 1212, 918 (ν_*C*–*B*_), 1061 (δ_*C*–*B*_), 981 (δ_*ip*,*C*–*H*_), 955 (ν_*B*–*F*_), 861, 841 (δ_*C*=*C*_), 632 (ν_*Mn*_). HRMS (ESI neg, *m*/*z*) 270.9593 ([M – K]^+^), calc. for C_8_H_4_O_3_MnBF_3_: 270.9592. UV–vis (CH_3_CN, [nm]) λ_max_ = 322 nm. Single crystals of **4** were obtained from acetonitrile and diethyl ether by diffusion crystallization at room temperature.

#### Cymantrenylboronic acid pinacol ester (**5**)

A round-bottom flask was equipped with 0.316 g of cymantrenylboronic acid (**2**) (1.27 mmol, 1.0 equiv), 0.154 g of pinacol (1.27 mmol, 1.0 equiv), 1.09 g of anhydrous sodium sulfate (6.0 equiv) and 30 mL of dichloromethane–ethyl acetate (5:1). The mixture was stirred overnight at ambient conditions. Sodium sulfate was filtered off and the solvents were removed in vacuo affording cymantrenylboronic acid pinacol ester (**5**) as yellow solid in 86.4% yield (0.363 g, 1.1 mmol). **5** is soluble in diethyl ether, dichloromethane, acetonitrile, and acetone. mp 75.2 °C. ^1^H NMR (400 MHz, CD_3_CN, ppm) δ = 1.28 (s, 12H), 4.98 (unresolved pseudo-t, 2H, C3/C4 of Cp), 5.13 (unresolved pseudo-t, 2H, C2/C5 of Cp). ^13^C NMR (75 MHz, CD_3_CN, ppm) δ = 24.9 (CH_3_), 85.0 (quaternary-carbon), 86.9 (C3/C4 of Cp), 92.9 (C2/C5 of Cp), 94.2 (ipso-carbon), 225.7 (carbonyl-carbon). ^55^Mn NMR (74 MHz, CD_3_CN, ppm) δ = −2138. ^11^B NMR (96 MHz, CD_3_CN, ppm) δ = 30.4. IR (ATR, cm^–1^) 3095 (ν_*C*–*H*(*Cp*)_), 2991, 2936, 2869 (ν_*C*–*H*(*Me*)_), 2015, 1915 (ν_*C*≡*O*_), 1506, 1492, 1414 (δ_*C*–*H*(*Me*)_), 1414, 1381, 1334, 1305, 1272, 1212 (ν_*C*–*B*_), 1167, 1126 (ν_*C*–*C*(*Pinacol*)_), 1063 (ν_*C*=*C*(*Cp*)_), 1334, 1305, 1063 (ν_*B*–*O*_), 1063, 1041, 1030 (ν_*CC*(*Cp*+*Pinacol*)_), 963 (δ_*ip*,*C*–*H*(*Cp*)_), 902 (ν_*C*–*O*(*Pinacol*)_), 847 (δ_*C*=*C*_), 775 (δ_*oop*,*C*–*H*(*Cp*)_), 661 (δ_*C*–*B*_), 626, 535 (ν_*Mn*_). HRMS (ESI pos, *m*/*z*) 330.0457, calc. for C_14_H_16_O_5_MnB: 330.0466. UV–vis (CH_3_CN, [nm]) λ_max_ = 324 nm. Single crystals of **5** were obtained from dichloromethane at room temperature.

#### 8-Tromanceniumyltrifluoridoborate (**7**)

A Schlenk flask, equipped with a cooling finger and a bubbler, was charged with 0.500 g of cymantrenylboronic acid pinacol ester (**5**) (1.52 mmol, 1.00 equiv), 209 μL of 1,3,5-cycloheptatriene (1.99 mmol, 1.32 equiv), and 35 mL of dry heptane. The apparatus was then irradiated with blue light (450 nm) and 370 W for 15 min until no further carbon monoxide evolution was observed. Note: A successful reaction is indicated by a color change from yellow to orange. Heptane was removed in vacuo. The intermediate highly air-sensitive η^6^-complex was dissolved in 60 mL of dichloromethane (abs) and cooled to 0 °C. Tritylium hexafluoridophosphate (0.774 g, 1.99 mmol, 1.32 equiv) was added in one portion, and the solution was stirred for 18 h under exclusion of light. Dichloromethane was concentrated in vacuo. After precipitation with diethyl ether, the air-stable product was filtered off and washed three times with 20 mL portions of diethyl ether. The product was dissolved with acetonitrile from the folded filter and the solvent was removed in vacuo affording **7** as red solid in 43% yield (0.183 g, 0.66 mmol). An overall quantum yield of 5.97% was calculated. Compound **7** is air-stable and soluble in acetone, acetonitrile, and dimethyl sulfoxide. mp 223.4 °C dec. ^1^H NMR (400 MHz, CD_3_CN, ppm) δ = 4.52 (unresolved pseudo-t, 2H, C3/C4 of Cp), 4.62 (unresolved pseudo-t, 2H, C2/C5 of Cp), 6.69 (s, 7H, C1–7 of Cht). ^13^C NMR (75 MHz, CD_3_CN, ppm) δ = 79.3 (C10/C11 of Cp), 82.1 (C9/C12 of Cp), 97.1 (C1–7 of Cht), C(8) not observed. ^19^F/^13^C-HSQC NMR (700 MHz, saturated solution in DMSO-*d*_6_, ppm δ = 95.8 (C1–7 of Cht), 96.8 C(8). ^55^Mn NMR (74 MHz, CD_3_CN, ppm) δ = 162. ^11^B NMR (96 MHz, CD_3_CN, ppm) δ = −15.8 (q, *J* = 46.6 Hz). ^19^F NMR (376 MHz, CD_3_CN, ppm) δ = −139.0 (q, *J*_1_ = 46 Hz). IR (ATR, cm^–1^) 3111 (ν_*C*–*H*(*Cp*)_), 3068, 3030 (ν_(*C*–*H*(*Cht*)_), 1447 (δ_*ip*, *C*–*H*(*Cht*)_), 1385, 1322 (ν_*C*=*C*(*Cp*)_), 1202 (ν_*C*–*B*_), 1049 (ν_*B*–*F*_), 1024, 995 (δ_*ip*,*C*–*H*(*Cp*)_), 969, 924, 900, 851, 816, 677 (scaffold), 635, 557 (δ_*B*–*F*_), 504, 471, 443 (ν_*Mn*_). HRMS (ESI neg, *m*/*z*) 312.9982 ([M + Cl]^−^), calc. for C_12_H_11_MnBF_3_Cl: 312.9980. UV–vis (CH_3_CN, [nm]) λ_max_ = 282 nm. Single crystals of **7** were obtained from a mixture of acetonitrile and diethyl ether at room temperature by diffusion crystallization.

#### 8-Tromanceniumylboronic acid pinacol ester triflate (**8**)

A Schlenk flask, equipped with a cooling finger and a bubbler, was charged with 0.250 g of cymantrenylboronic acid pinacol ester (**5**) (0.758 mmol, 1.00 equiv), 104 μL of 1,3,5-cycloheptatriene (0.997 mmol, 1.32 equiv), and 30 mL of dry heptane. The apparatus was then irradiated with blue light (450 nm) and 370 W for 33 min until no further carbon monoxide evolution was observed. Note: A successful reaction is indicated by a color change from yellow to red-orange. Heptane was removed in vacuo. The intermediate highly air-sensitive complex was dissolved in 40 mL of dichloromethane (abs) and cooled to 0 °C. Tritylium triflate (0.500 g, 1.274 mmol, 1.68 equiv) was added in argon counter-current and the added amount was calculated by back-weighing. The mixture was stirred for 1 h under exclusion of light. Dichloromethane was concentrated in vacuo. After precipitation with diethyl ether, the air-stable product was filtered off and washed three times with 10 mL portions of diethyl ether. The product was dissolved with acetonitrile from the folded filter and the solvent was removed in vacuo affording **8** as purple solid in 79.5% yield (0.293 g, 0.603 mmol). An overall quantum yield of 1.36% was calculated. Compound **8** is air-stable and soluble in dichloromethane, acetone, acetonitrile, and dimethyl sulfoxide. mp 139.3 °C dec. ^1^H NMR (400 MHz, CD_3_CN, ppm) δ = 1.43 (s, 12H, CH_3_), 4.83 (pseudo-t, 2H, C10/C11 of Cp, *J*_1_ = 2.0 Hz, *J*_2_ = 1.6 Hz), 4.92 (pseudo-t, 2H, C9/C12 of Cp, *J* = 2.0 Hz), 6.85 (s, 7H, C1–7 of Cht). ^13^C NMR (75 MHz, CD_3_CN, ppm) δ = 25.4 (CH_3_), 81.5 (C10/C11 of Cp), 82.7 (C9/C12 of Cp), 97.8 (C1–7 of Cht), 156.3 (ipso-carbon of Cp). ^55^Mn NMR (74 MHz, CD_3_CN, ppm) δ = 415. ^11^B NMR (96 MHz, CD_3_CN, ppm) δ = 13.2. IR (ATR, cm^–1^) 3075, 2983 (ν_*C*–*H*(*Me*)_), 1483, 1449 (ν_*C*–*B*_), 1400 (ν_*C*=*C*(*Cp*)_), 1390, 1345 (δ_*C*–*H*(*Me*)_), 1373, 1326 (ν_*B*–*O*_), 1245, 1151 (ν_*CC*_), 1134, 965 (ν_*C*–*O*_), 900 (δ_*oop*,*C*–*H*(*Cht*)_), 871 (δ_*oop*,*C*–*H*(*Cp*)_), 851, 830, 771 (δ_*B*–*O*_), 704, 573 (δ_*C*–*B*_), 516 (δ_*C*–*O*–*B*_), 486, 457, 437, 410 (ν_*Mn*_). HRMS (ESI pos, *m*/*z*) 337.1165 ([M – OTf]^+^), calc. for C_18_H_23_O_2_MnB: 337.1166. UV–vis (CH_3_CN, [nm]) λ_max1_ = 279 nm, λ_max2_ = 576 nm. Single crystals of **8** were obtained from a mixture of acetonitrile and diethyl ether at room temperature by diffusion crystallization.

#### 8-Tromanceniumylboronic acid triflate (**9**)

A round-bottom flask was charged with 0.090 g of purple 8-Tromanceniumylboronic acid pinacol ester (**8**) (0.185 mmol, 1 equiv), 10 mL of THF–water (9:1) and 362 μL of a saturated sodium carbonate solution. The mixture was stirred for 30 min at room temperature, while a pink intermediate precipitated. The precipitation was completed by adding 20 mL of diethyl ether to the mixture. The solvents were removed with a pipet and the residue was dissolved in 10 mL of water and washed three times with 20 mL portions of diethyl ether in a separating funnel in order to remove pinacol remains completely. The organic layer was discarded and the aqueous phase was acidified with 139 μL (9 equiv) of HCl (37%) until the pH value was approximately 1. A color change from pink to purple was observed. Water was removed in vacuo and the product was dissolved in acetonitrile and filtered off to remove sodium chloride. The solvent was removed in vacuo affording **9** as purple resin in 79% yield (0.059 g, 0.146 mmol). Compound **9** is air-stable and soluble in acetone, acetonitrile, water, and dimethyl sulfoxide. mp 76.2 °C. ^1^H NMR (400 MHz, CD_3_CN, ppm) δ = 4.89 (s, 4H, C8–12 of Cp), 6.40 (s, 2H of OH), 6.81 (s, 7H, C1–7 of Cht). ^13^C NMR (75 MHz, CD_3_CN, ppm) δ = 81.8 (C10/C11 of Cp), 83.3 (C9/C12 of Cp), 97.9 (C1–7 of Cht). ^55^Mn NMR (74 MHz, CD_3_CN, ppm) δ = 367. ^11^B NMR (96 MHz, CD_3_CN, ppm) δ = 29.3. IR (ATR, cm^–1^) 3101, 3079, 3035 (ν_*C*–*H*_), 1473, 1446, (ν_*C*=*C*(*Cht*)_), 1409, 1238 (ν_*C*–*B*_), 1388 (ν_*B*–*O*_), 899, 863 (δ_*ip*,*O*–*H*_), 830 (δ_*C*=*C*(*Cp*)_), 738, 720, 706 (δ_*oop*,*C*–*H*(*Cp*+*Cht*)_), 575, 516, 480 (δ_*oop*,*O*–*H*_), 449, 411 (ν_*Mn*_). HRMS (ESI pos, *m*/*z*) 255.0381 ([M – OTf]^+^), calc. for C_12_H_13_O_2_MnB: 255.0384. UV–vis (CH_3_CN, [nm]) λ_max1_ = 283 nm, λ_max2_ = 549 nm. Single crystals of **9** were obtained from a mixture of acetonitrile and diethyl ether at 4 °C by diffusion crystallization.

#### 8-Aminotromancenium triflate (**10**)

A Teflon microwave vessel was charged with 64.8 mg of 8-Tromanceniumylboronic acid pinacol ester (**8**) (0.133 mmol, 1 equiv), 86.6 mg of sodium azide (1.33 mmol, 10 equiv), 26.6 mg of copper(II) acetate monohydrate and 30 mL of dry ethanol. The mixture was saturated with argon and stirred for 30 min until almost everything was dissolved whereby a color darkening from orange to dark brown was observed. The reaction mixture was then irradiated in a microwave synthesis reactor with 600 W for 10 min at 105 °C (the ramp time is 2 min). The reaction mixture was allowed to cool to room temperature before the vessel was opened and then decanted into a round-bottom flask in order to remove the solvent in vacuo. The product was extracted with ethyl acetate and filtered off. Ethyl acetate was removed in vacuo and the residue was washed three times with 5 mL portions of diethyl ether, which were removed by pipet affording **10** as pink solid in 72.4% yield (36.2 mg, 0.096 mmol). Spectroscopic data (see Supporting Information) concur with recently published data.^1^

#### 8-Chlorotromancenium triflate (**12**)

A 10 mL round-bottom flask was charged with 30.8 mg of 8-aminotromancenium triflate (**10**) (0.082 mmol, 1 equiv) and dissolved in 4 mL of HCl (37%). The solution was cooled to −40 °C before an aqueous solution of sodium nitrite (11,33 mg, 0.165 mmol, 2 equiv) was added. An initial color change from pink to yellow was observed. The reaction was stirred for 10 min at −40 °C and then allowed to warm to room temperature, whereby a further color change from yellow to pink as well as gas evolution was observed. The hydrochloric acid was removed in vacuo and the residue was washed three times with 5 mL portions of diethyl ether. The product was dissolved in acetonitrile and filtered off through a paper filter. The solvent was removed in vacuo affording **12** in 43.2% yield (0.014 g, 0.035 mmol). mp 83.3 °C. ^1^H NMR (400 MHz, CD_3_CN, ppm) δ = 4.73 (unresolved pseudo-t, 2H, C10/C11 of Cp), 5.06 (unresolved pseudo-t, 2H, C9/C12 of Cp), 6.92 (s, 7H, C1–7 of Cht). ^13^C NMR (75 MHz, CD_3_CN, ppm) δ = 76.1 (C10/C11 of Cp), 78.8 (C9/C12 of Cp), 99.2 (C1–7 of Cht). ^55^Mn NMR (74 MHz, CD_3_CN, ppm) δ = 367. IR (ATR, cm^–1^) 3106, 3075 (ν_*C*–*H*(*Cp*)_), 3036 (ν_*C*–*H*(*Cht*)_), 1561, 1481 (ν_*C*=*C*(*Cht*)_), 1449 (δ_*ip*,*C*–*H*(*Cht*)_), 1432, 1402, 1371 (ν_*C*=*C*(*Cp*)_), 1432, 1371, 1165 (ν_*C*–*Cl*_), 1022 (δ_*ip*, *C*–*H*(*Cp*)_), 906 (δ_*oop*,*C*–*H*(*Cp*+*Cht*)_), 884 (δ_*ip*, *C*=*C*(*Cp*+*Cht*)_), 851 (δ_*oop*, *C*–*H*(*Cp*)_), 832 (δ_*oop*,*C*–*H*(*Cht*)_), 531 (δ_*oop*,*C*=*C*(*Cp*)_), 514 (δ_*oop*,*C*=*C*(*Cht*)_), 496, 480, 449, 422 (ν_*Mn*_). HRMS (ESI pos, *m*/*z*) 244.9917 ([M – OTf]^+^), calc. for C_12_H_11_MnCl: 244.9924. UV–vis (CH_3_CN, [nm]) λ_max1_ = 379 nm, λ_max2_ = 548 nm. Single crystals of **12** were obtained from a mixture of acetonitrile and diethyl ether at 4 °C by diffusion crystallization.

## Supporting Information Available

Spectra (^1^H/^13^C/^11^B/^19^F/^55^Mn NMR, IR, HRMS, UV–vis), cyclic voltammograms, DFT results (PDF)

## Accession Codes

CCDC 2159454–2159460 contain the supplementary crystallographic data for this paper. These data can be obtained free of charge via www.ccdc.cam.ac.uk/data_request/cif, or by emailing data_request@ccdc.cam.ac.uk, or by contacting The Cambridge Crystallographic Data Centre, 12 Union Road, Cambridge CB2 1EZ, UK; fax: +44 1223 336033.
