# Supplementary material for: Borylated Cymantrenes and Tromancenium Salts with Unusual Reactivity
Source: Organometallics. 2022 May 13;41(11):1464–73. doi: 10.1021/acs.organomet.2c00179 (PMC9490842; doi:10.1021/acs.organomet.2c00179)
Supplement: Supplementary file 1 — om2c00179_si_001.pdf [file om2c00179_si_001.pdf]

# Supporting Information

# Borylated Cymantrenes and Tromancenium Salts with Unusual Reactivity

Reinhard Thaler,[#] Holger Kopacka,[#] Klaus Wurst,[#] Thomas Müller,[◊] Dennis F. Dinu,[#] Klaus R. Liedl,[#] Florian R. Neururer,[#] Stephan Hohloch,[#] and Benno Bildstein[#,*]

[#]Institute of General, Inorganic and Theoretical Chemistry, Center for Chemistry and Biomedicine, University of Innsbruck, Innrain 80-82, 6020 Innsbruck, Austria

[◊]Institute of Organic Chemistry, Center for Chemistry and Biomedicine, University of Innsbruck, Innrain 80-82, 6020 Innsbruck, Austria

*E-mail for B.B.: benno.bildstein@uibk.ac.at

**1. Analytical Section** ........ **S1**

**Cymantrene (1)** ........ **S1**

**Cymantrenylboronic acid (2)** ........ **S2**

**Potassium cymantrenyltrifluoridoborate (4)** ........ **S6**

**Cymantrenylboronic acid pinacol ester (5)** ........ **S10**

**8-Tromanceniumylboronic acid pinacol ester hexafluoridophosphate (6)** ........ **S14**

**8-Tromanceniumyltrifluoridoborate (7)** ........ **S15**

**8-Tromanceniumylboronic acid pinacol ester triflate (8)** ........ **S20**

**8-Tromanceniumylboronic acid triflate (9)** ........ **S24**

**8-Aminotromancenium triflate (10)** ........ **S28**

**8-Chlorotromancenium triflate (12)** ........ **S29**

**2. Cyclic Voltammetry** ........ **S32**

**8-Tromanceniumyltrifluoridoborate (7)** ........ **S32**

**8-Tromanceniumylboronic acid pinacol ester triflate (8)** ........ **S33**

**8-Tromanceniumylboronic acid triflate (9)** ........ **S35**

**8-Chlorotromancenium triflate (12)** ........ **S36**

## 1. Analytical Section

### Cymantrene (1)

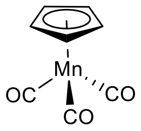


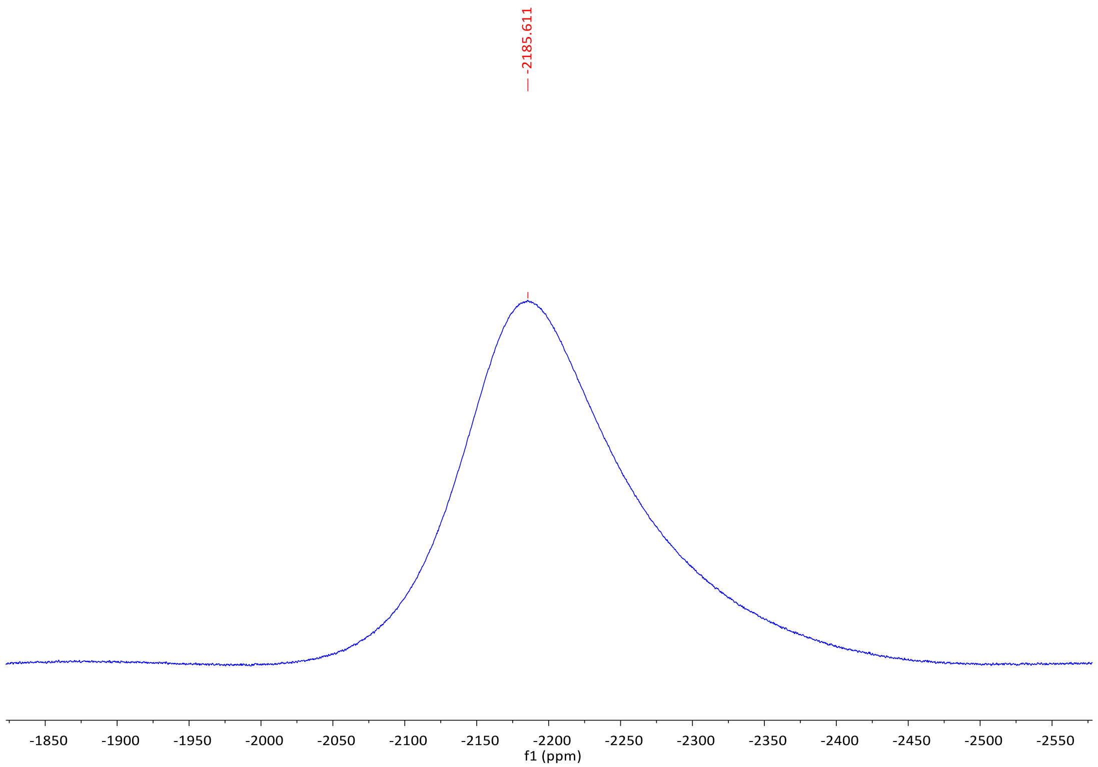


Figure S1. $^{55}$Mn-NMR spectrum of **1.**

## Cymantrenylboronic acid (2)

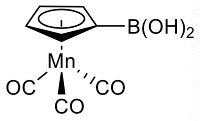


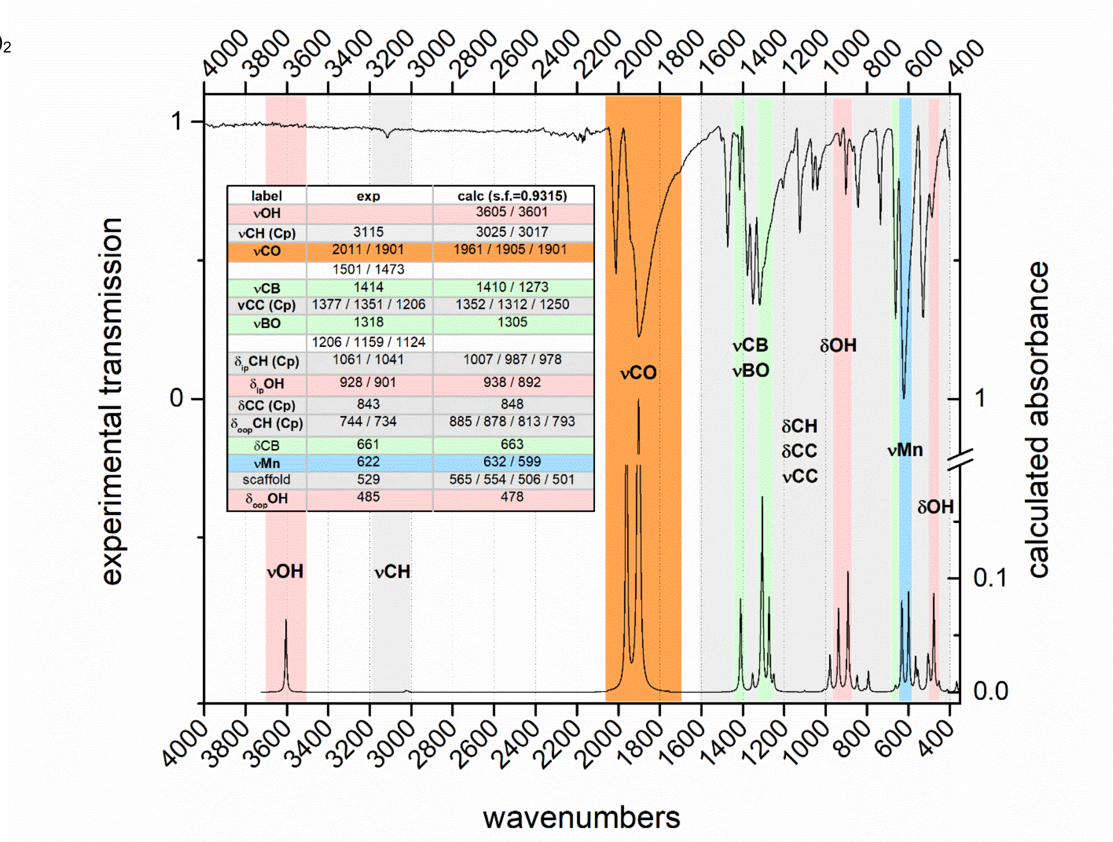


| label | exp | calc (s.f.=0.9315) |
|---|---|---|
| νOH | | 3605 / 3601 |
| νCH (Cp) | 3115 | 3025 / 3017 |
| νCO | 2011 / 1901 | 1961 / 1905 / 1901 |
| | 1501 / 1473 | |
| νCB | 1414 | 1410 / 1273 |
| νCC (Cp) | 1377 / 1351 / 1206 | 1352 / 1312 / 1250 |
| νBO | 1318 | 1305 |
| | 1206 / 1159 / 1124 | |
| $\delta_{ip}$CH (Cp) | 1061 / 1041 | 1007 / 987 / 978 |
| $\delta_{ip}$OH | 928 / 901 | 938 / 892 |
| δCC (Cp) | 843 | 848 |
| $\delta_{oop}$CH (Cp) | 744 / 734 | 885 / 878 / 813 / 793 |
| δCB | 661 | 663 |
| νMn | 622 | 632 / 599 |
| scaffold | 529 | 565 / 554 / 506 / 501 |
| $\delta_{oop}$OH | 485 | 478 |

Figure S2. IR spectrum of **2**.

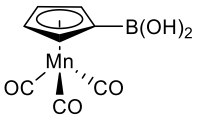


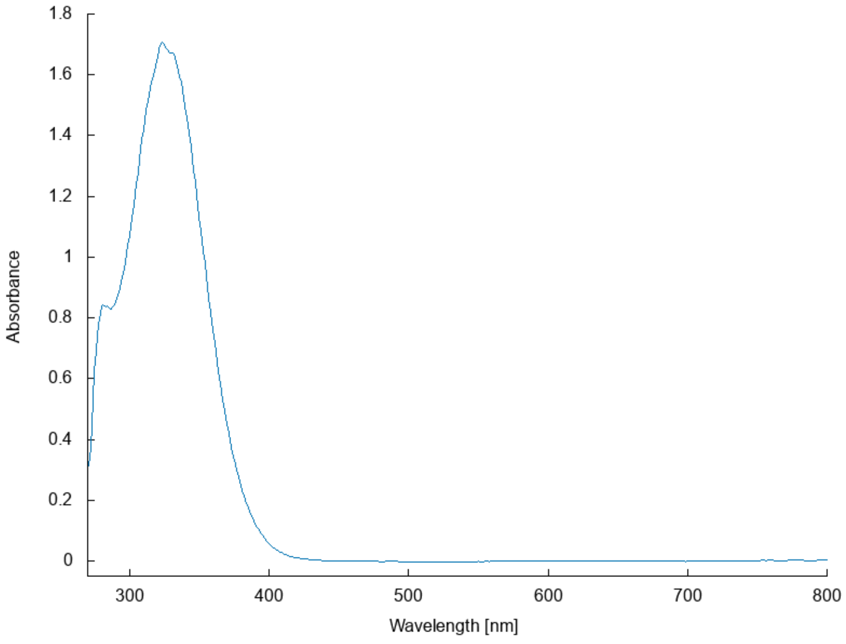


Figure S3. UV/vis spectrum of **2**.

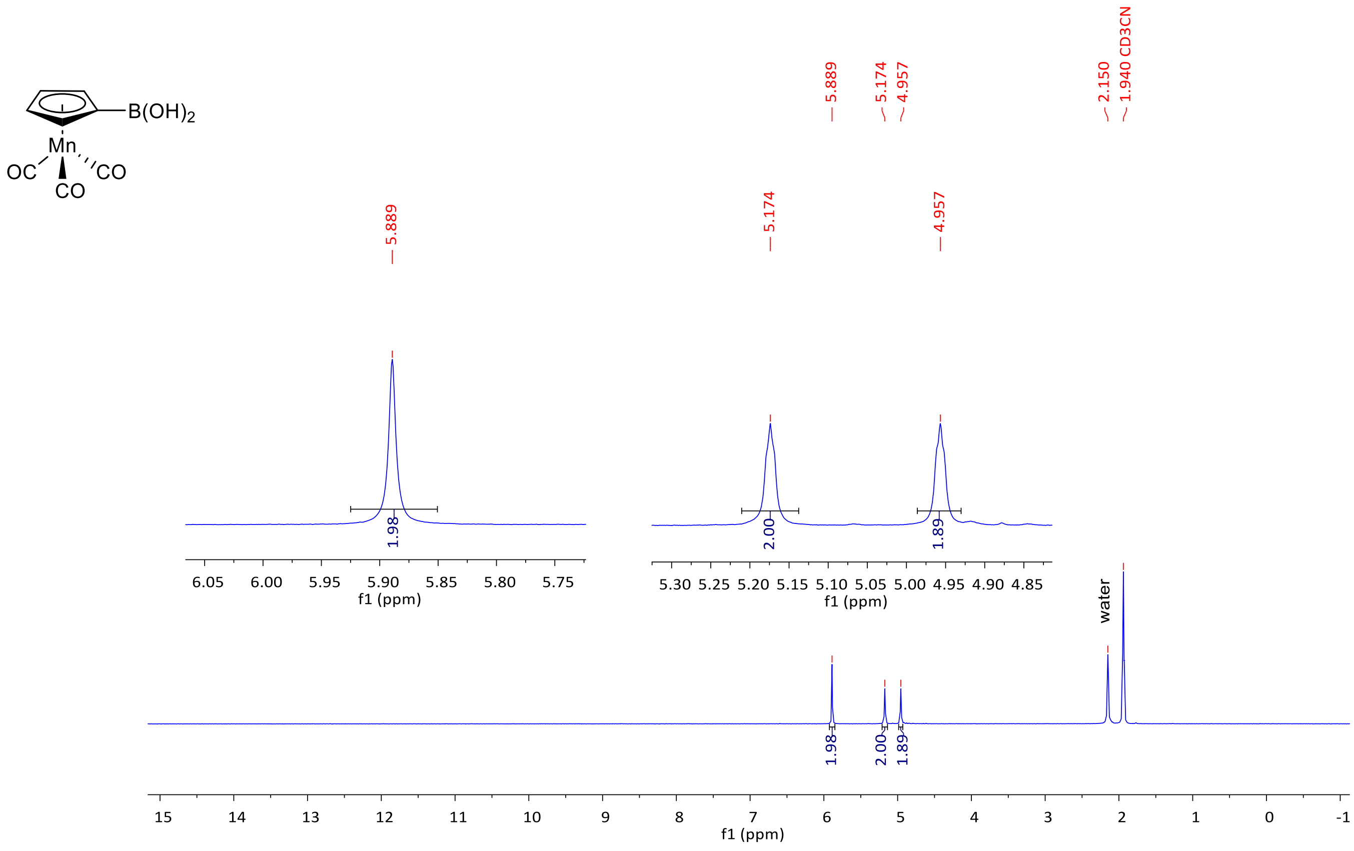


Figure S4. $^{1}$H-NMR spectrum of **2**.

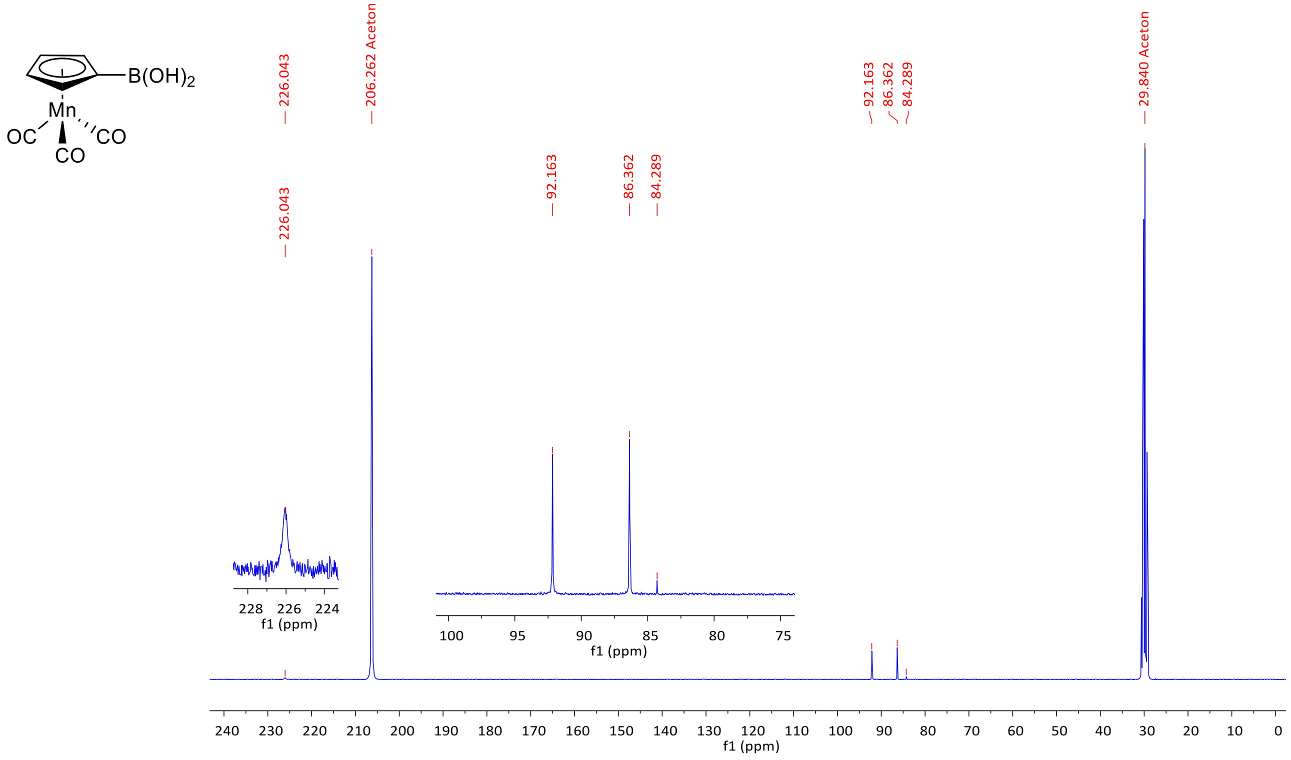


Figure S5. $^{13}$C-NMR spectrum of **2**.

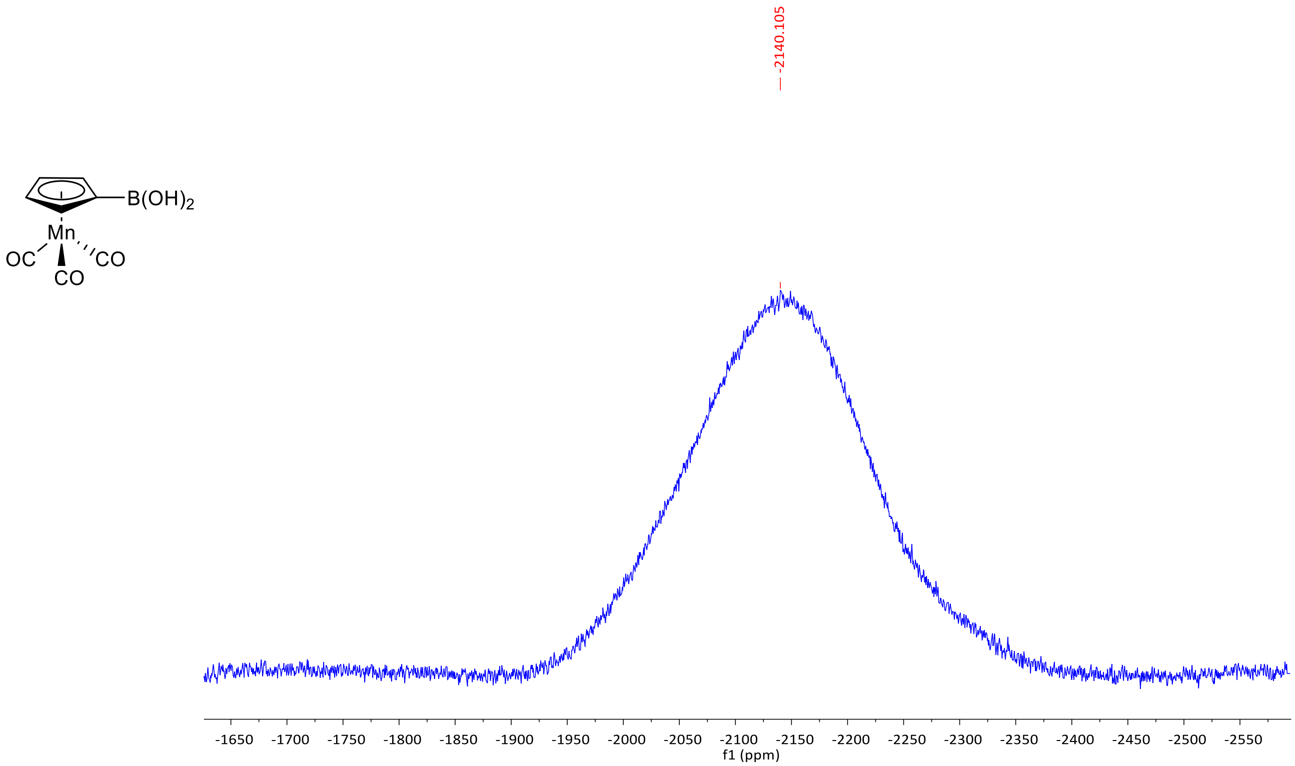


Figure S6. $^{55}$Mn-NMR spectrum of **2.**

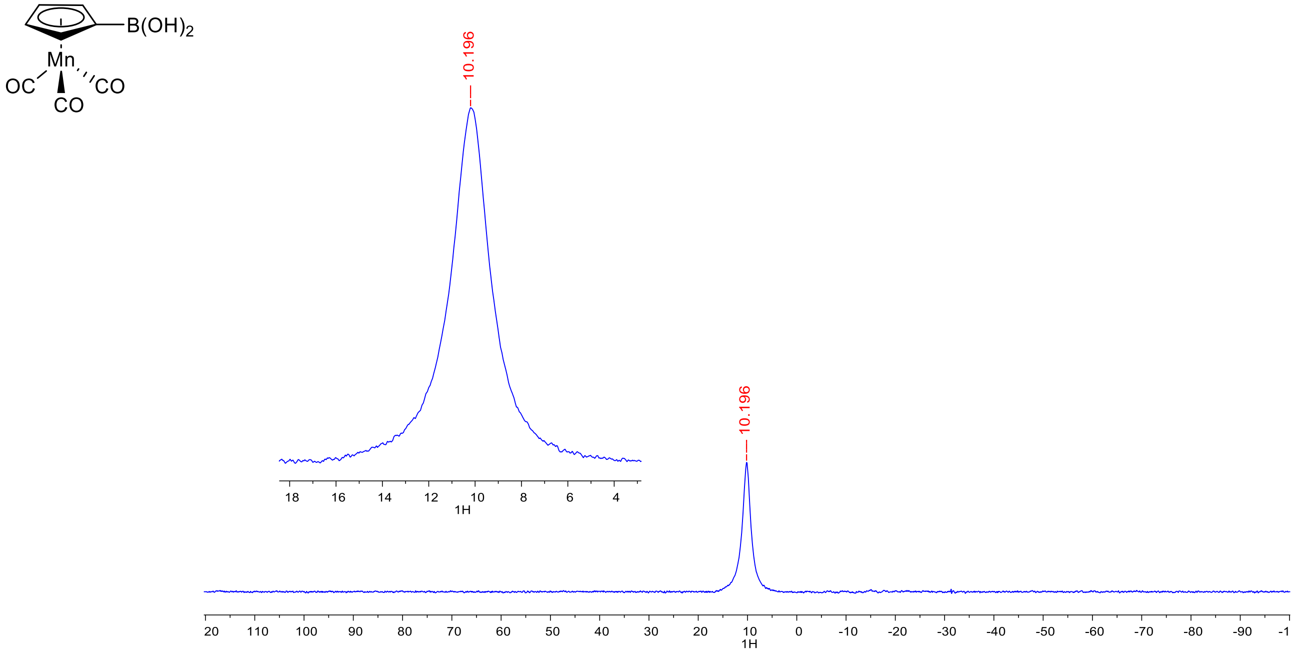


Figure S7. $^{11}$B-NMR spectrum of **2**.

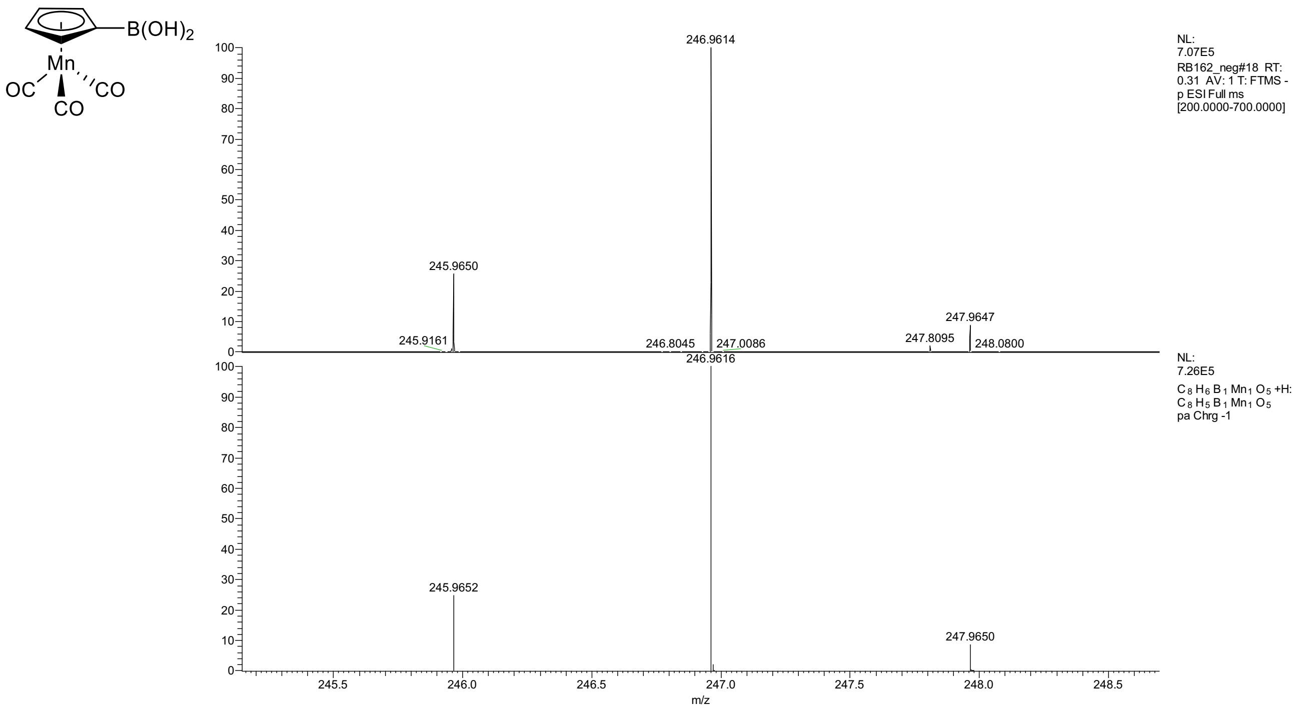


Figure S8. MS (ESI neg, [m/z]; *top*: experimental, *bottom*: simulated) of **2**.

**Potassium cymantrenyltrifluoridoborate (4)**

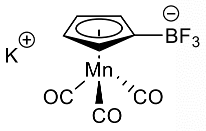


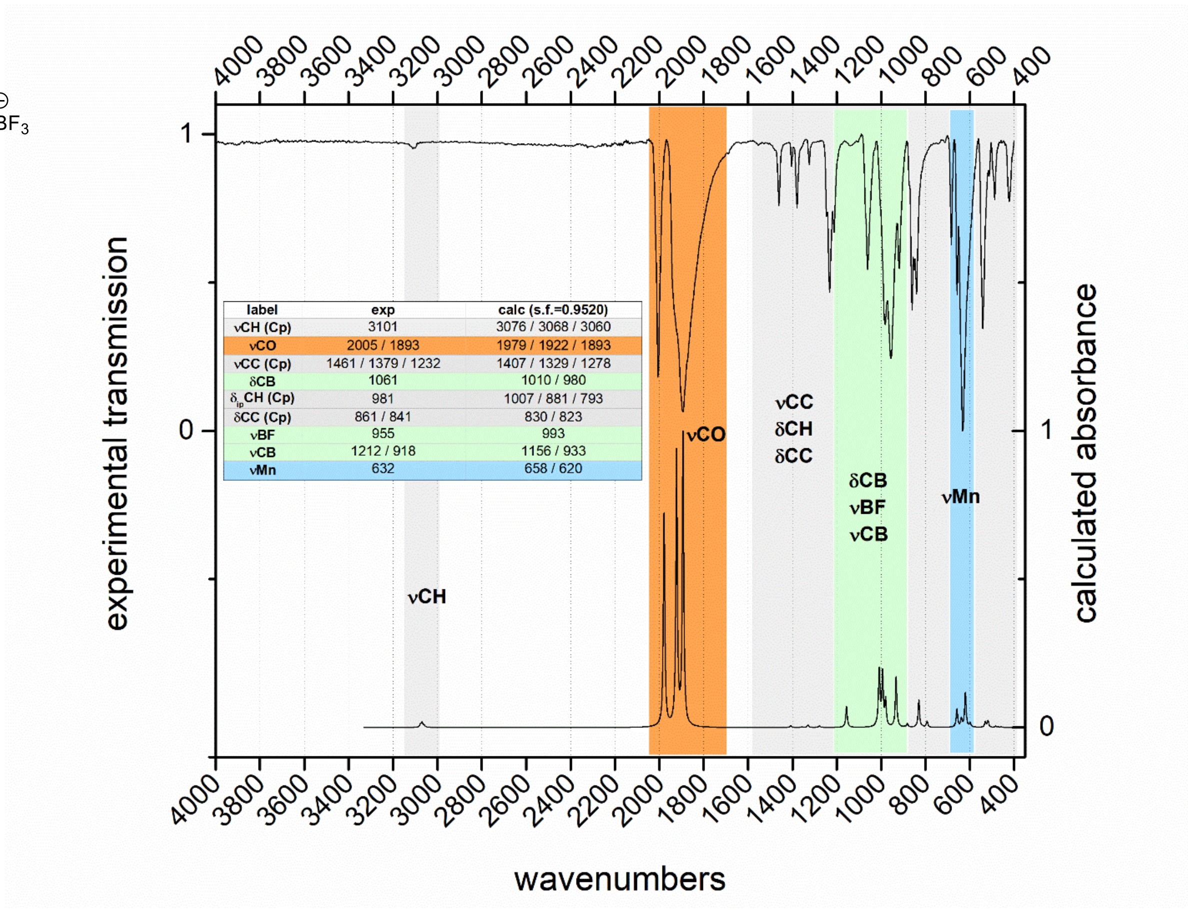


| label | exp | calc (s.f.=0.9520) |
| --- | --- | --- |
| νCH (Cp) | 3101 | 3076 / 3068 / 3060 |
| νCO | 2005 / 1893 | 1979 / 1922 / 1893 |
| νCC (Cp) | 1461 / 1379 / 1232 | 1407 / 1329 / 1278 |
| δCB | 1061 | 1010 / 980 |
| $\delta_{ip}$CH (Cp) | 981 | 1007 / 881 / 793 |
| δCC (Cp) | 861 / 841 | 830 / 823 |
| νBF | 955 | 993 |
| νCB | 1212 / 918 | 1156 / 933 |
| νMn | 632 | 658 / 620 |

Figure S9. IR spectrum of **4**.

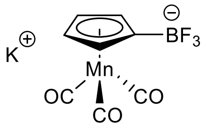


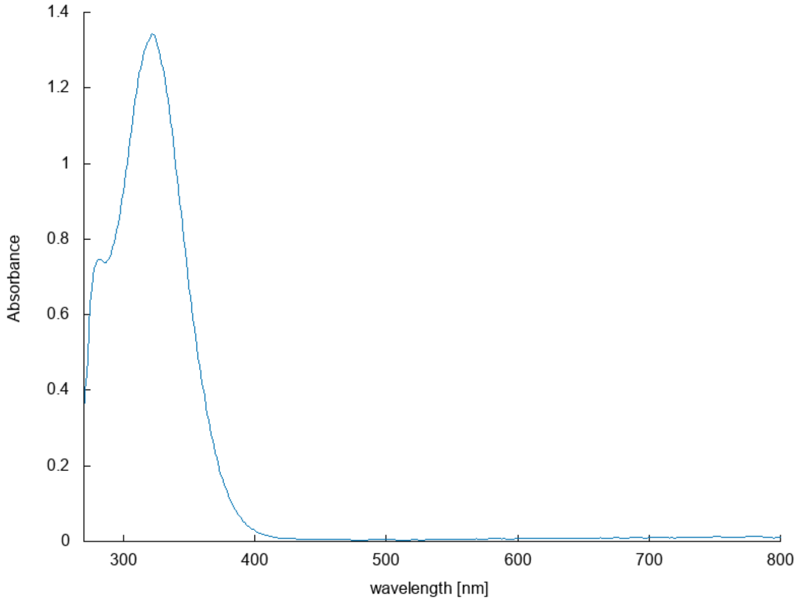


Figure S10. UV/vis spectrum of **4**.

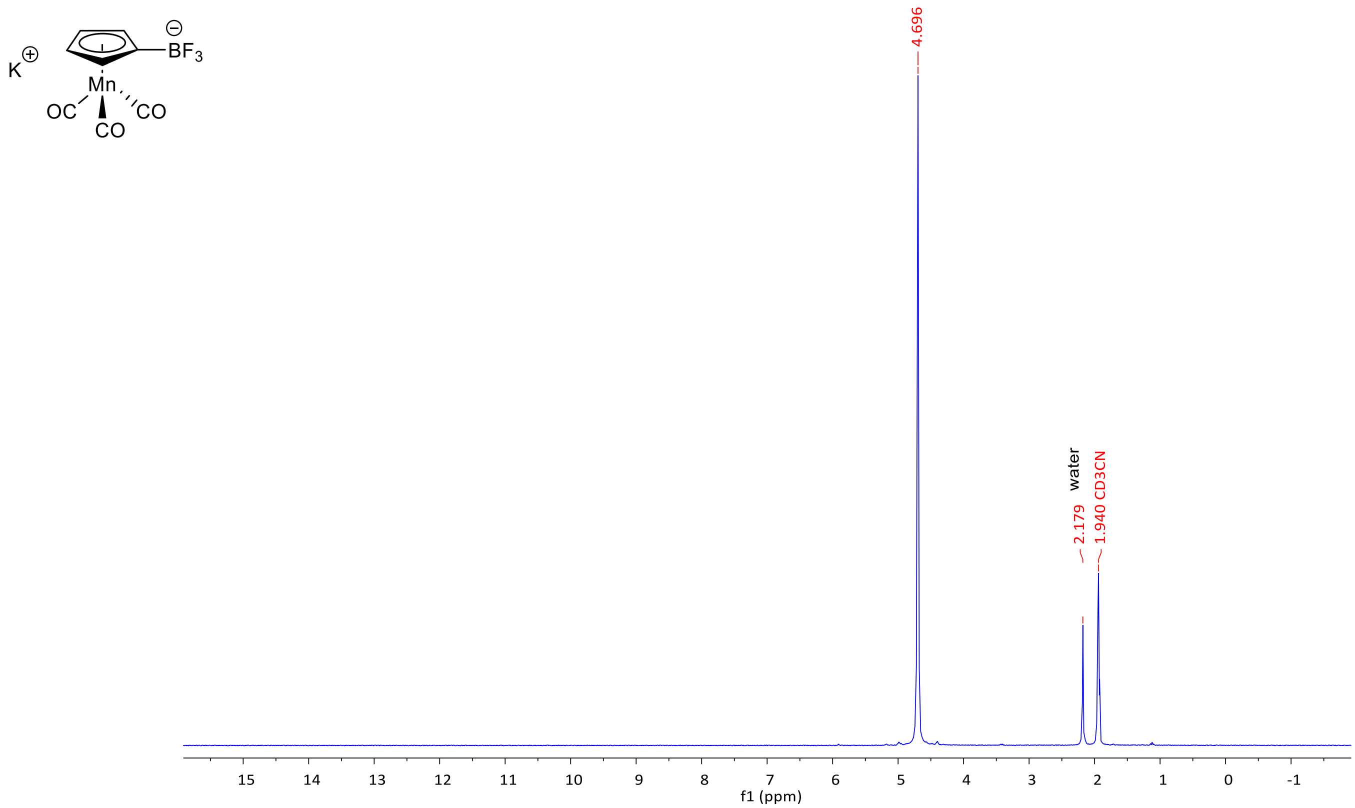


Figure S11. $^{1}$H-NMR spectrum of **4**.

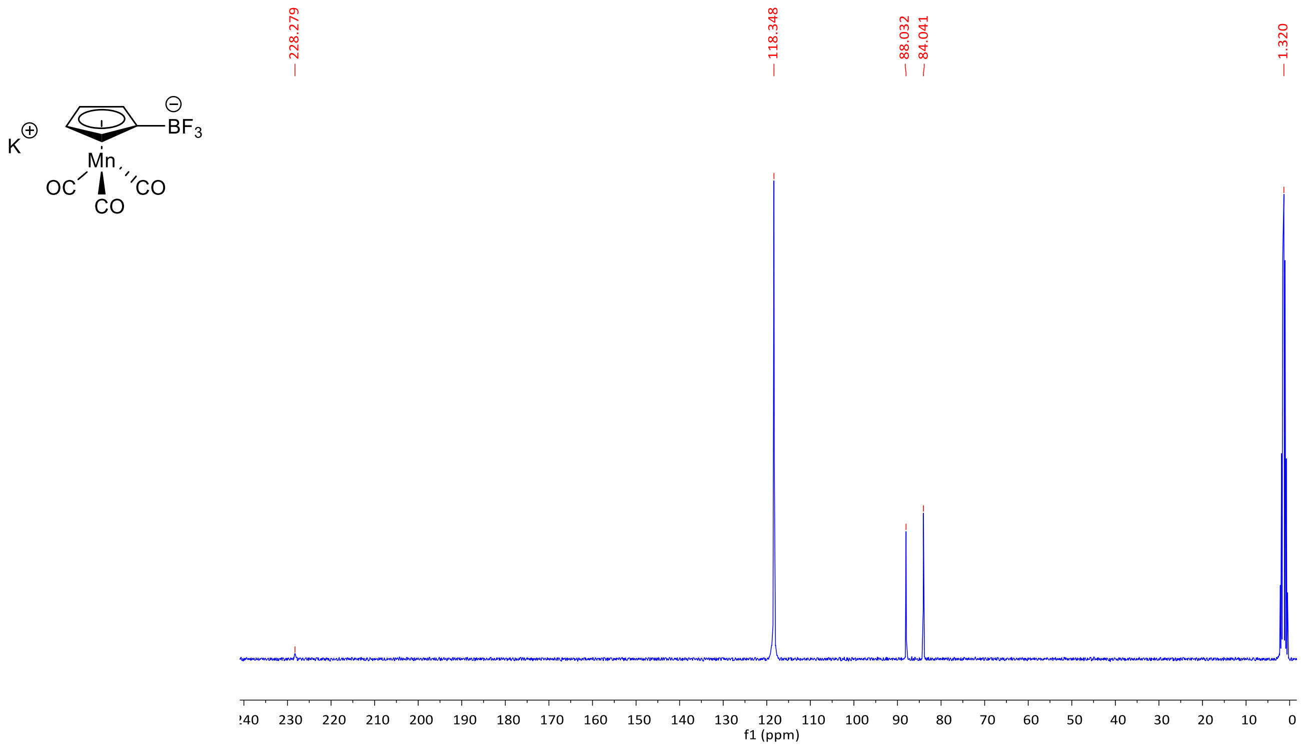


Figure S12. $^{13}$C-NMR spectrum of **4**.

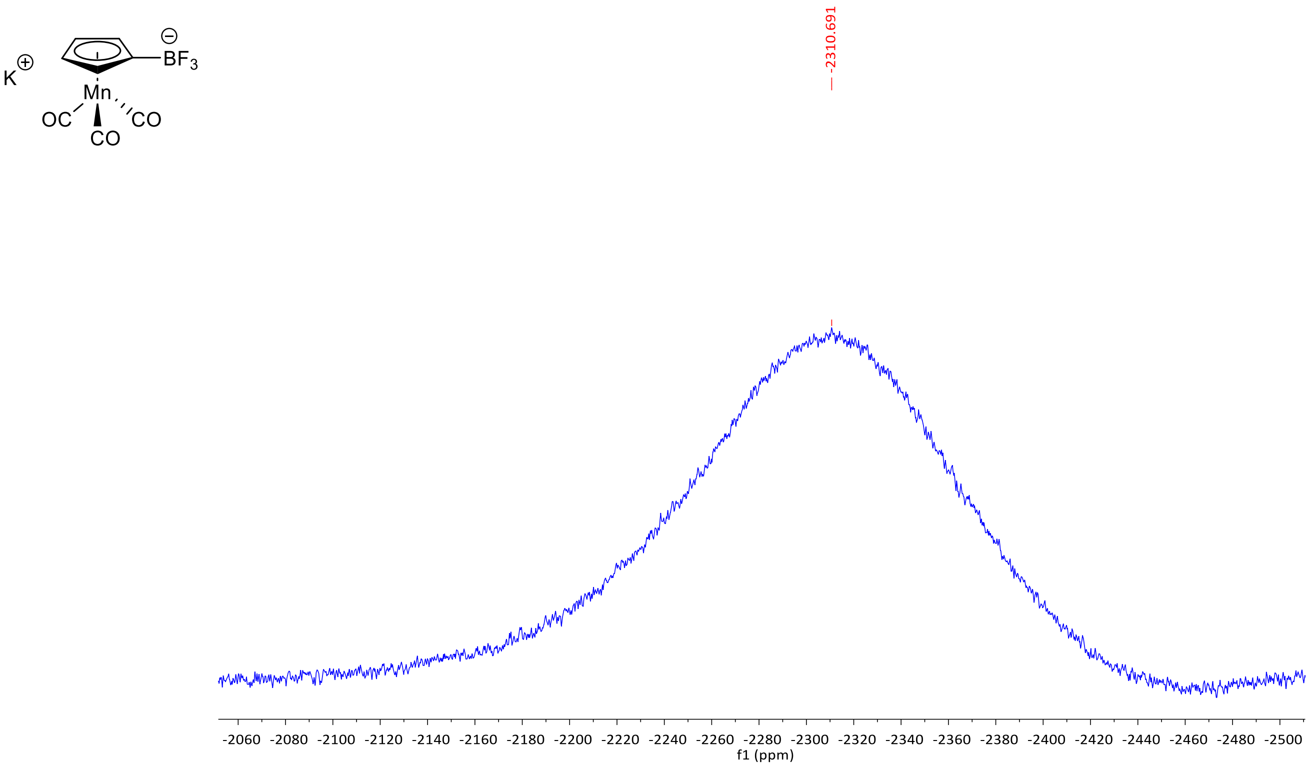


Figure S13. $^{55}$Mn-NMR spectrum of **4**.

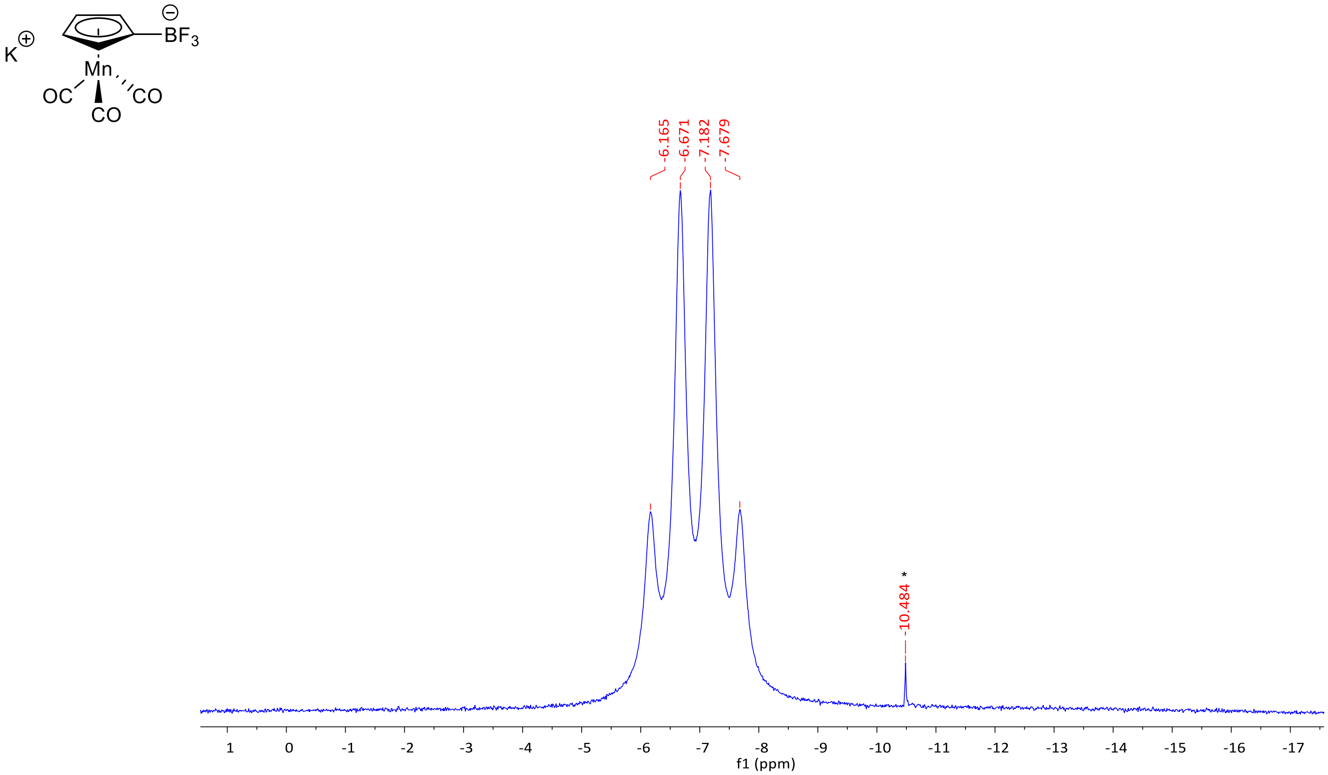


Figure S14. $^{11}$B-NMR spectrum of **4**.

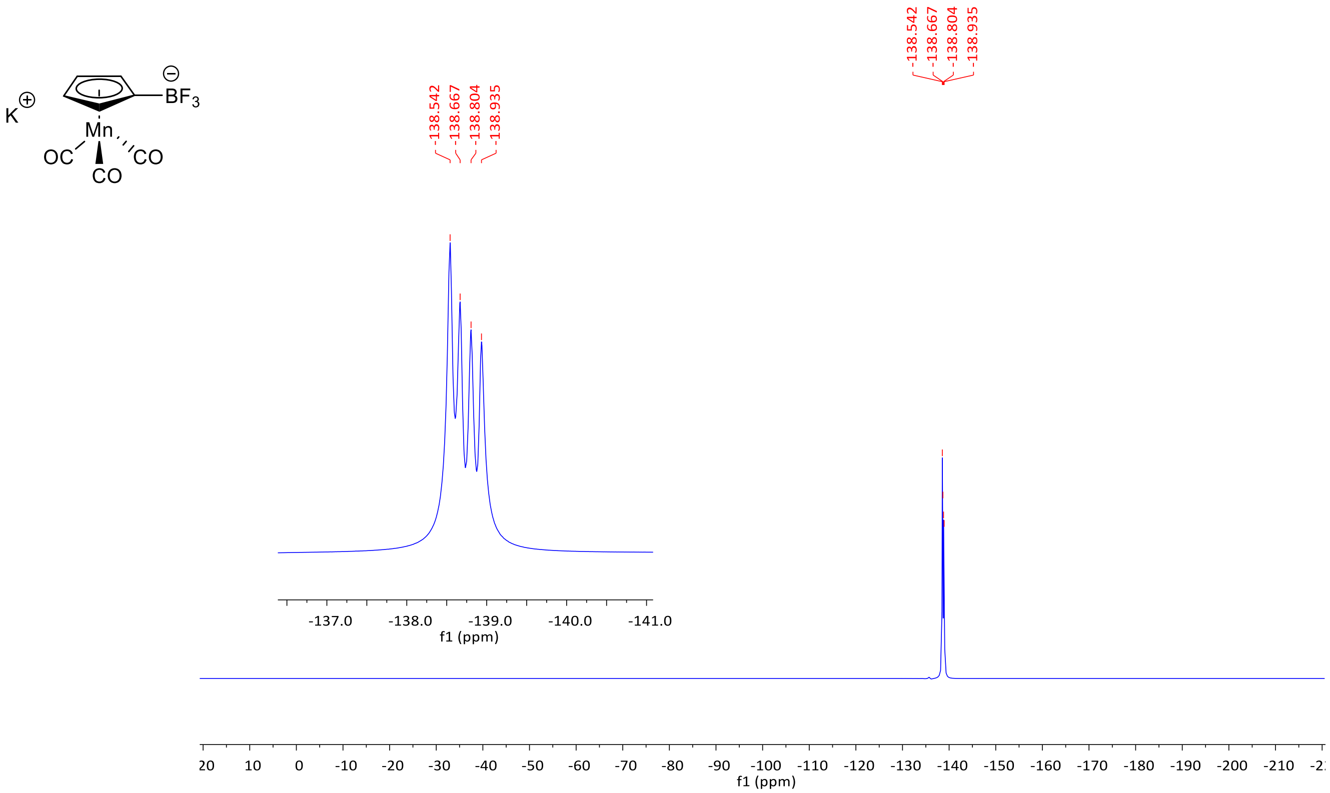


Figure S15. $^{19}$F-NMR spectrum of **4**.

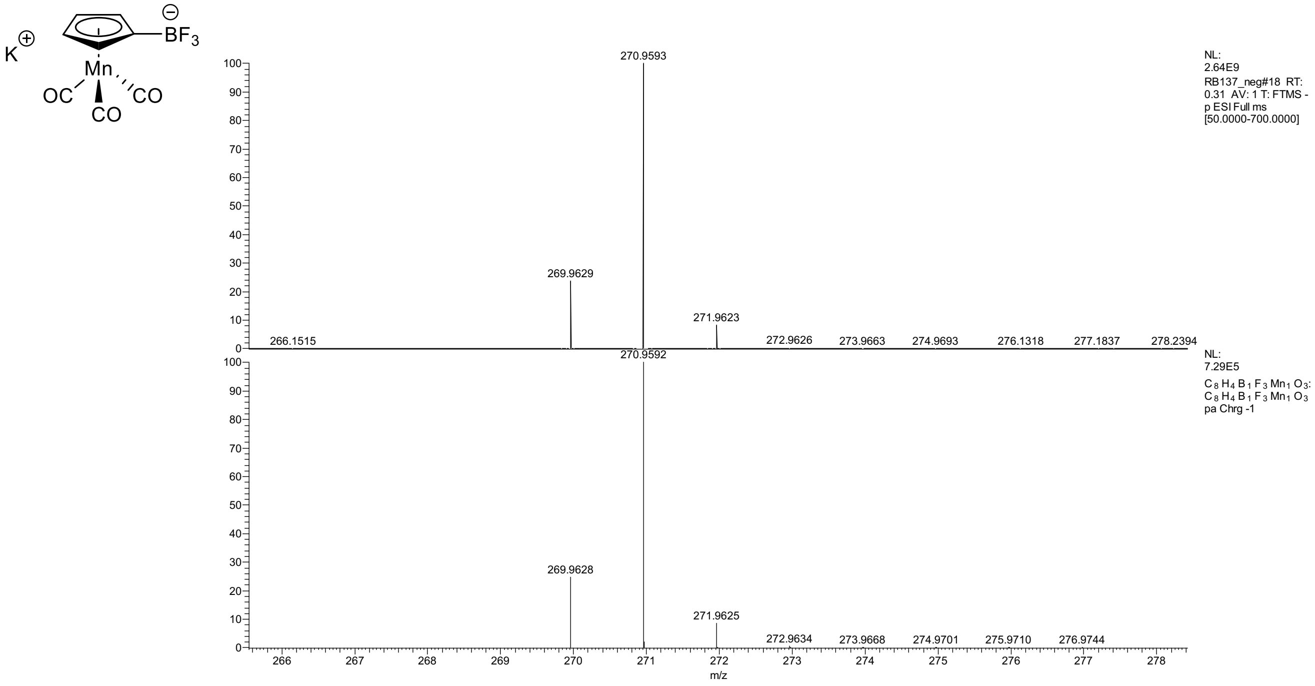


Figure S16. MS (ESI neg, [m/z]; *top*: experimental, *bottom*: simulated) of **4**.

**Cymantrenylboronic acid pinacol ester (5)**

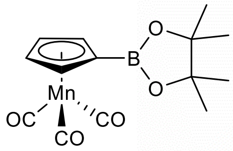

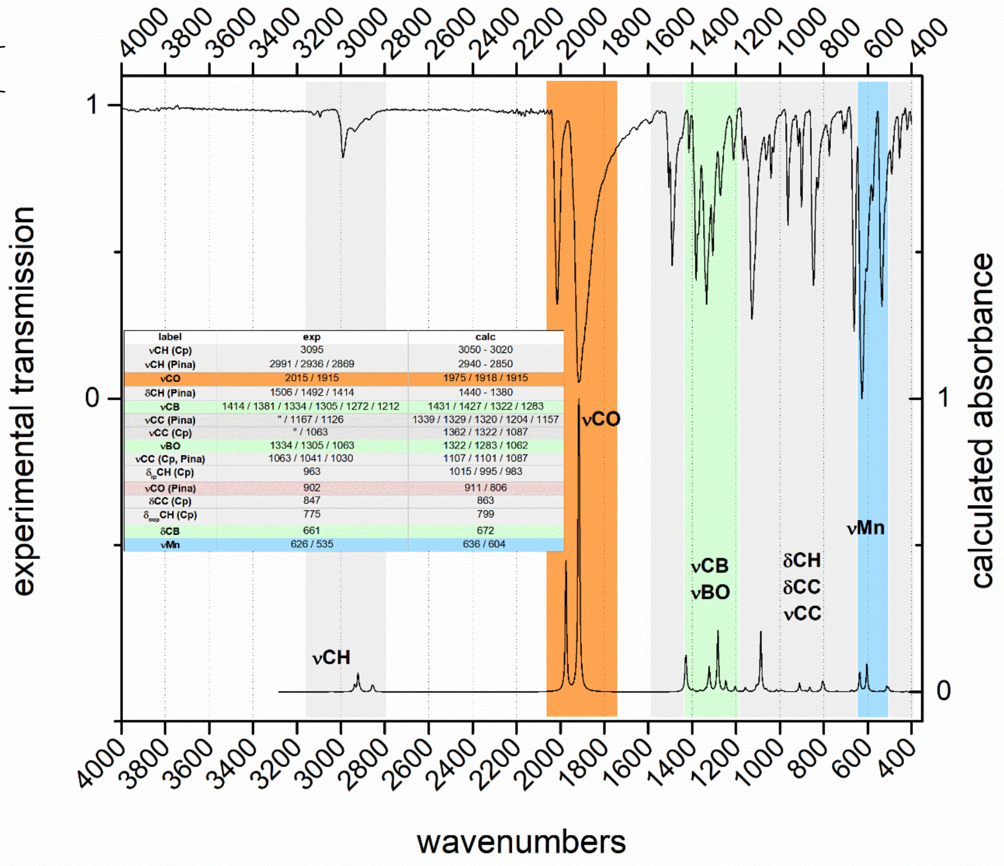


| label | exp | calc |
|---|---|---|
| νCH (Cp) | 3095 | 3050 - 3020 |
| νCH (Pina) | 2991 / 2936 / 2869 | 2940 - 2850 |
| νCO | 2015 / 1915 | 1975 / 1918 / 1915 |
| δCH (Pina) | 1506 / 1492 / 1414 | 1440 - 1380 |
| νCB | 1414 / 1381 / 1334 / 1305 / 1272 / 1212 | 1431 / 1427 / 1322 / 1283 |
| νCC (Pina) | " / 1167 / 1126 | 1339 / 1329 / 1320 / 1204 / 1157 |
| νCC (Cp) | " / 1063 | 1362 / 1322 / 1087 |
| νBO | 1334 / 1305 / 1063 | 1322 / 1283 / 1062 |
| νCC (Cp, Pina) | 1063 / 1041 / 1030 | 1107 / 1101 / 1087 |
| $\delta_{ip}$CH (Cp) | 963 | 1015 / 995 / 983 |
| νCO (Pina) | 902 | 911 / 806 |
| δCC (Cp) | 847 | 863 |
| $\delta_{oop}$CH (Cp) | 775 | 799 |
| δCB | 661 | 672 |
| νMn | 626 / 535 | 636 / 604 |

Figure S17. IR spectrum of **5**.

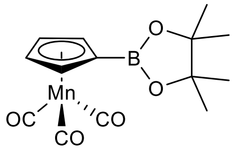

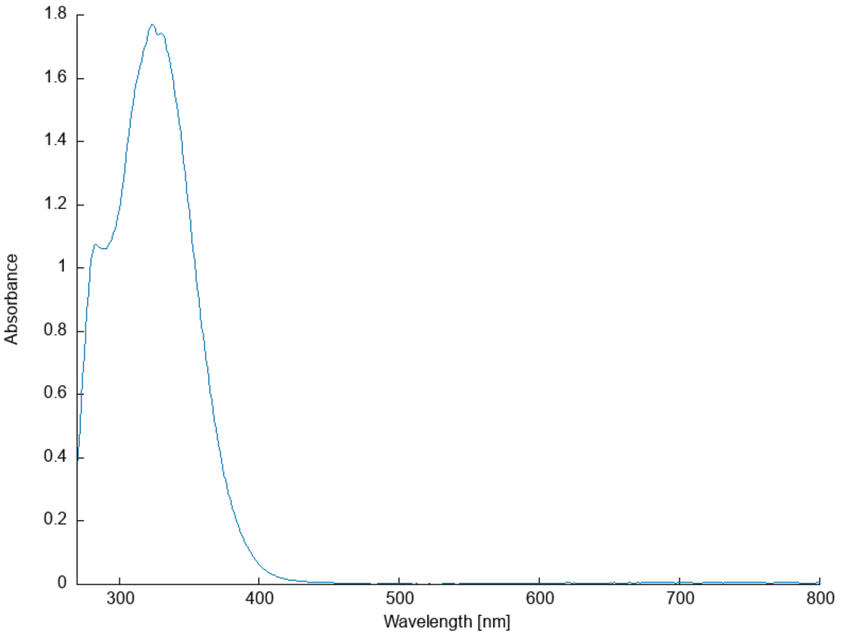


Figure S18. UV/vis spectrum of **5**.

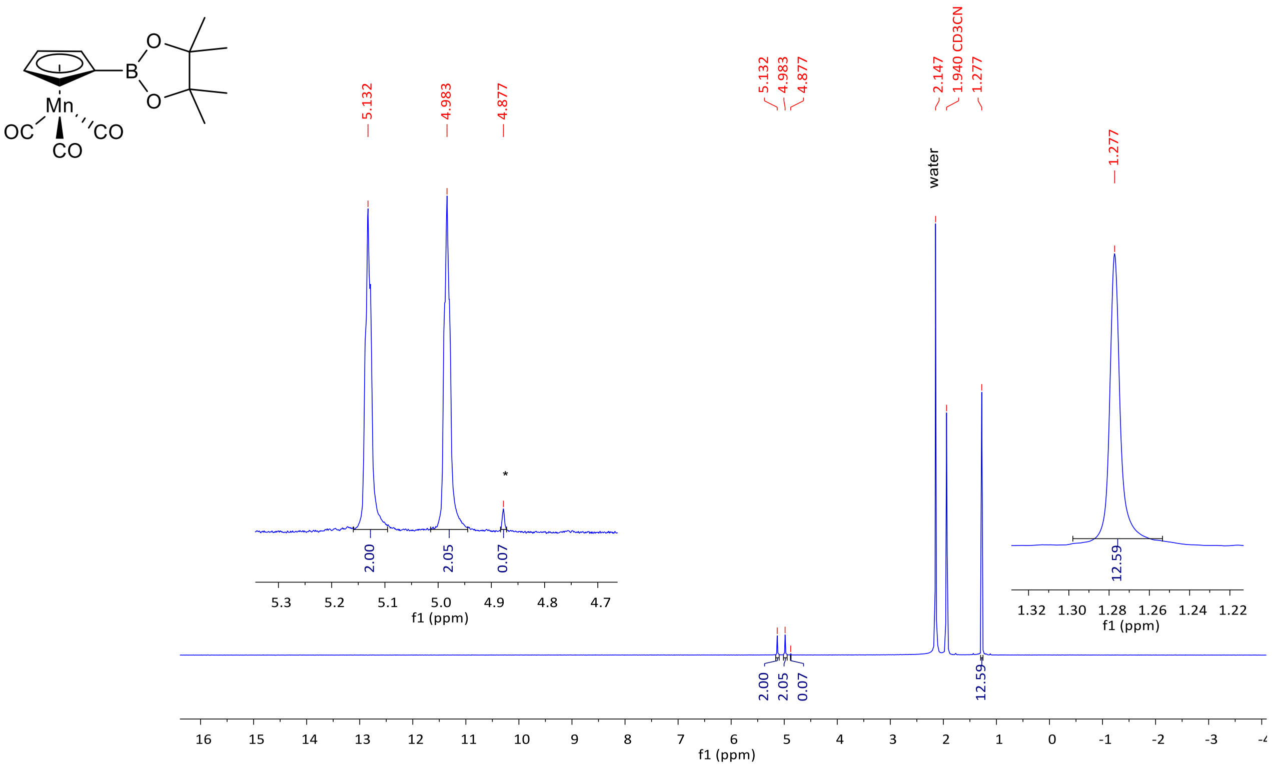


Figure S19. $^{1}$H-NMR spectrum of **5** (*marked:* traces of unsubstituted cymantrene).

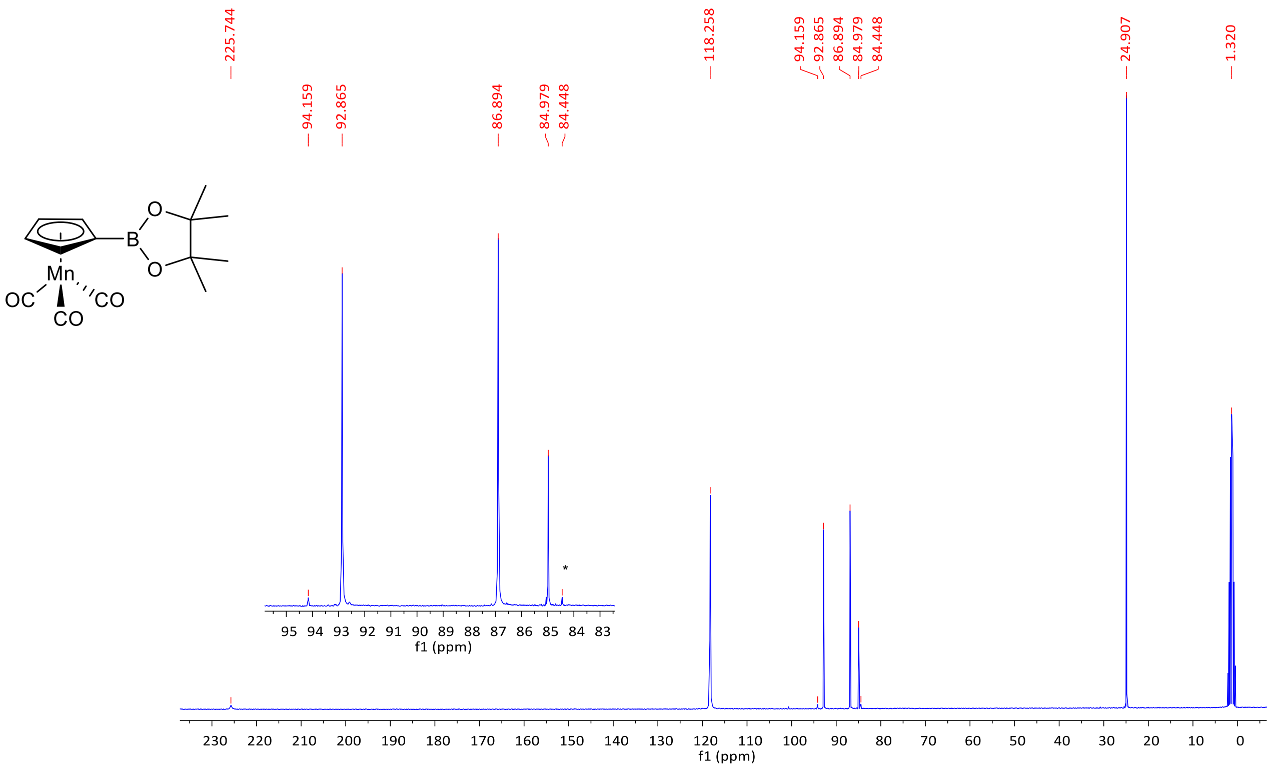


Figure S20. $^{13}$C-NMR spectrum of **5** (*marked:* traces of unsubstituted cymantrene).

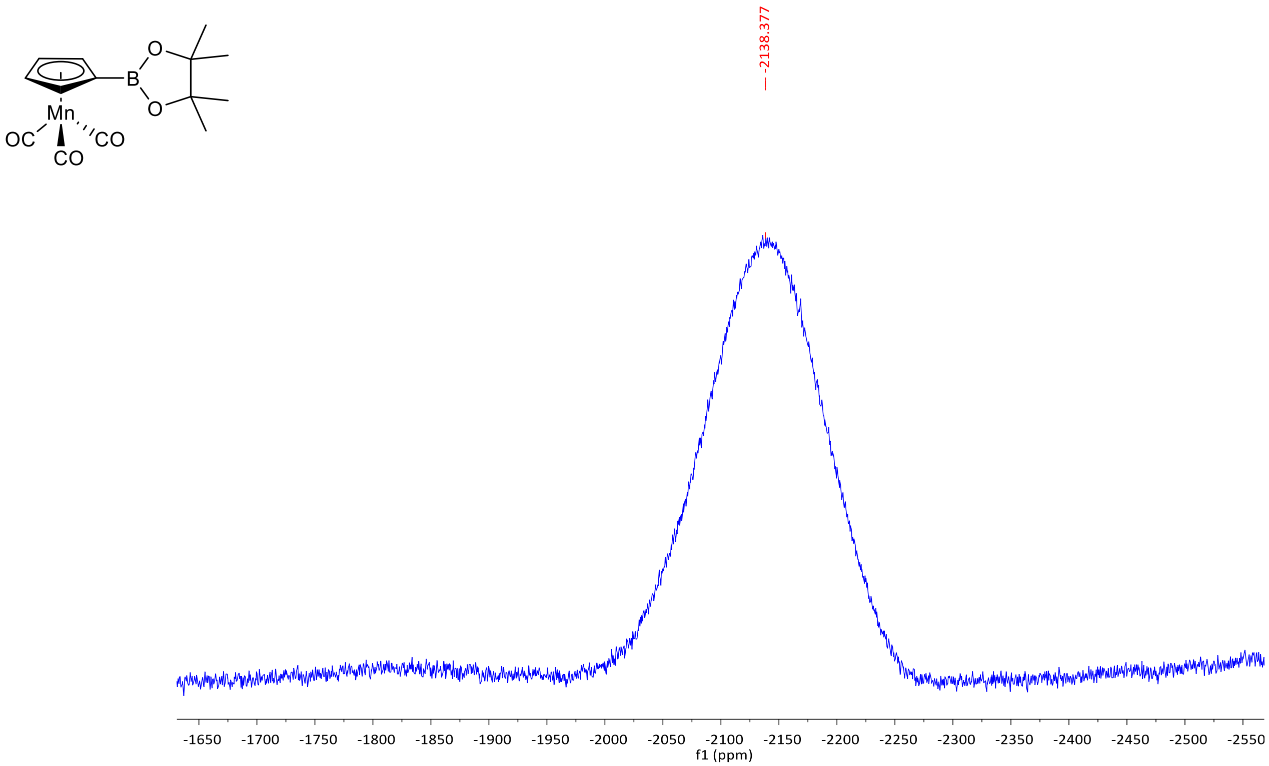


Figure S21. $^{55}$Mn-NMR spectrum of **5**.

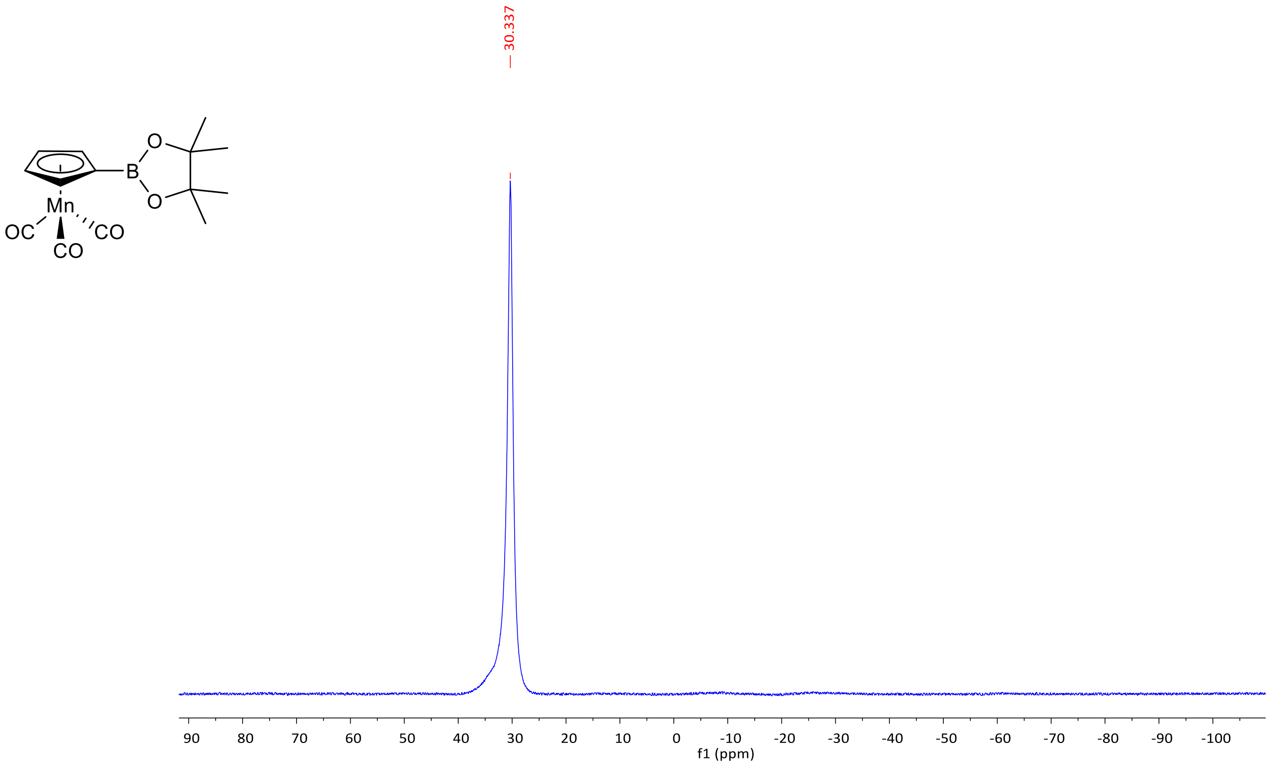


Figure S22. $^{11}$B-NMR spectrum of **5**.

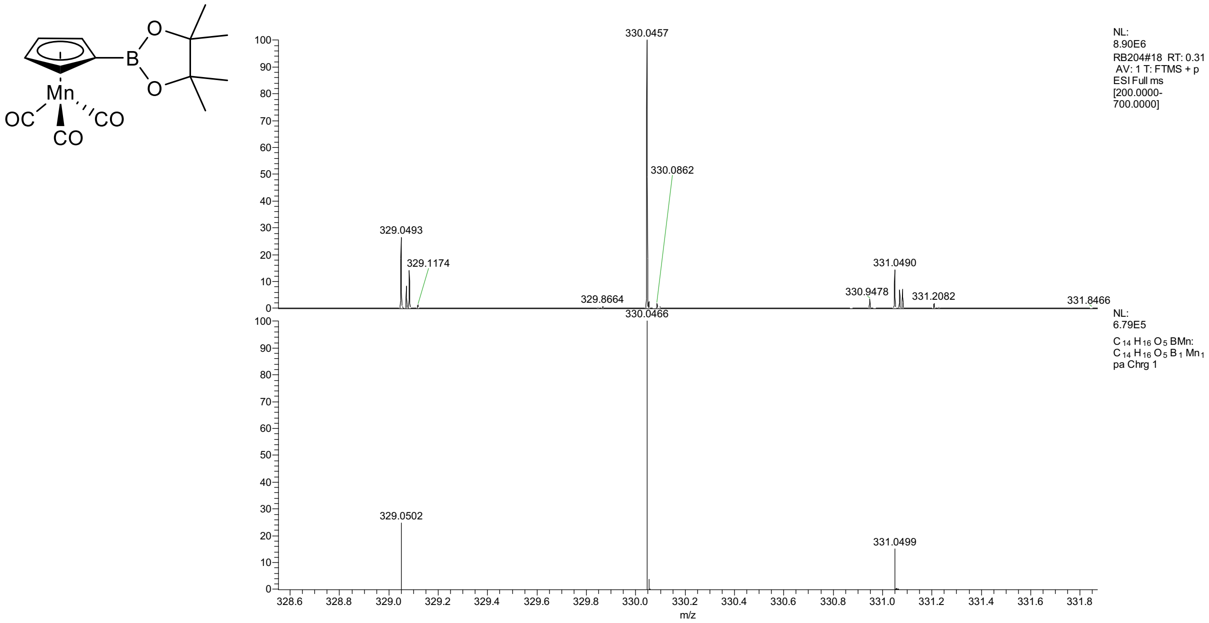


Figure S23. MS (ESI pos, [m/z]; *top*: experimental, *bottom*: simulated) of **5**.

**8-Tromanceniumylboronic acid pinacol ester hexafluoridophosphate (6)**

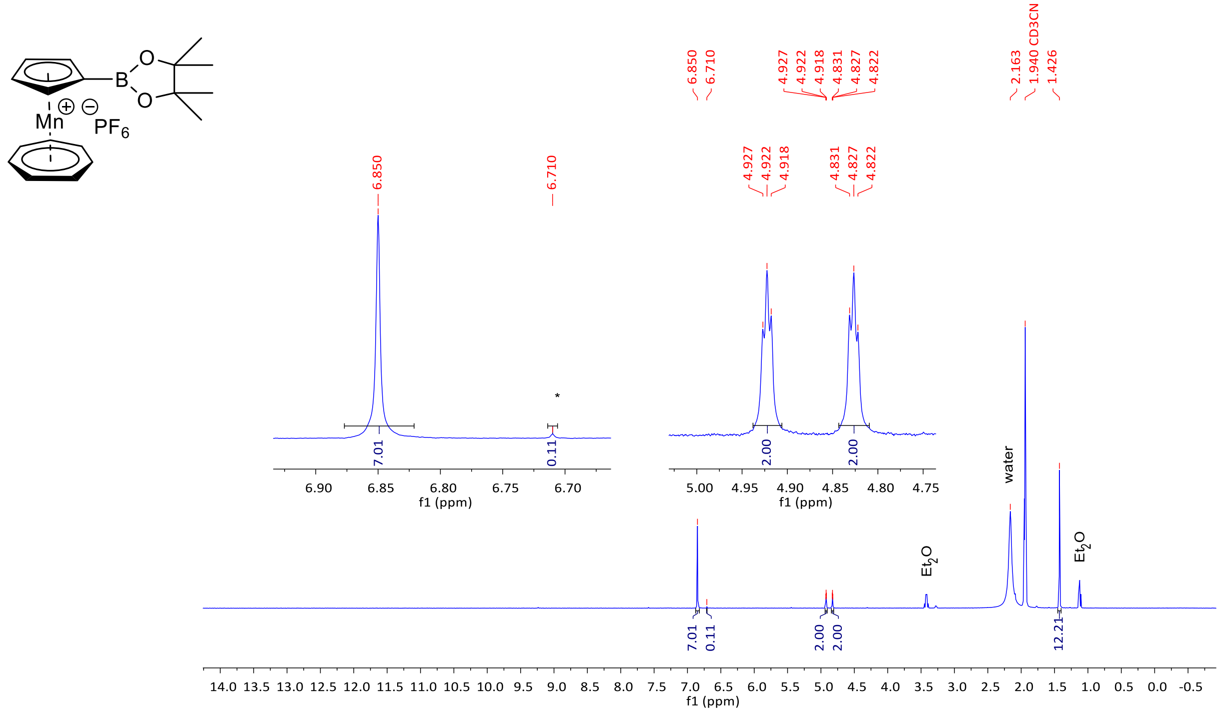


Figure S24. $^{1}$H-NMR spectrum of **6** (marked: traces of 8-tromanceniumyltrifluoridoborate **7**).

**8-Tromanceniumyltrifluoridoborate (7)**

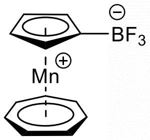


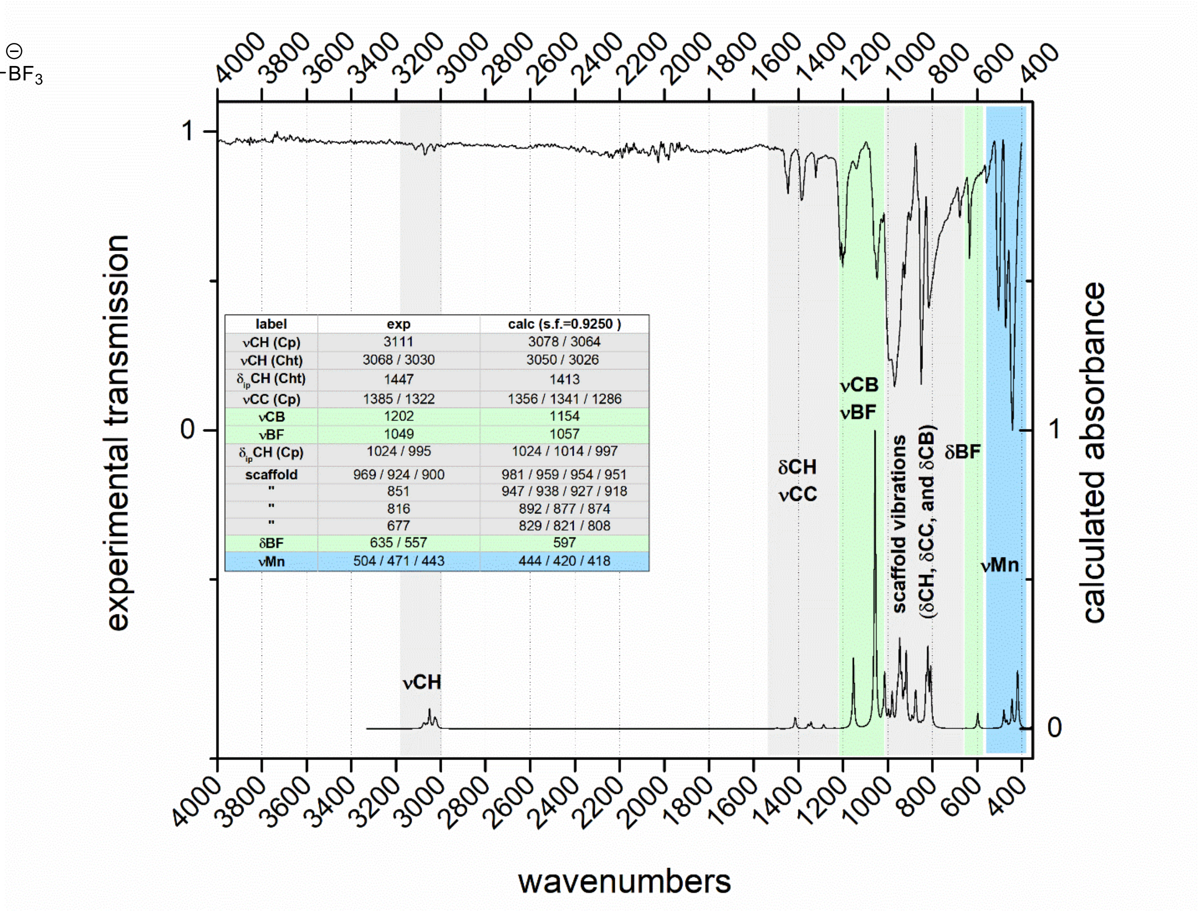


| label | exp | calc (s.f.=0.9250 ) |
|---|---|---|
| νCH (Cp) | 3111 | 3078 / 3064 |
| νCH (Cht) | 3068 / 3030 | 3050 / 3026 |
| $\delta_{ip}$CH (Cht) | 1447 | 1413 |
| νCC (Cp) | 1385 / 1322 | 1356 / 1341 / 1286 |
| νCB | 1202 | 1154 |
| νBF | 1049 | 1057 |
| $\delta_{ip}$CH (Cp) | 1024 / 995 | 1024 / 1014 / 997 |
| scaffold | 969 / 924 / 900 | 981 / 959 / 954 / 951 |
| " | 851 | 947 / 938 / 927 / 918 |
| " | 816 | 892 / 877 / 874 |
| " | 677 | 829 / 821 / 808 |
| δBF | 635 / 557 | 597 |
| νMn | 504 / 471 / 443 | 444 / 420 / 418 |

Figure S25. IR spectrum of **7**.

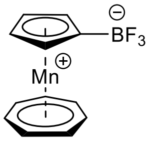


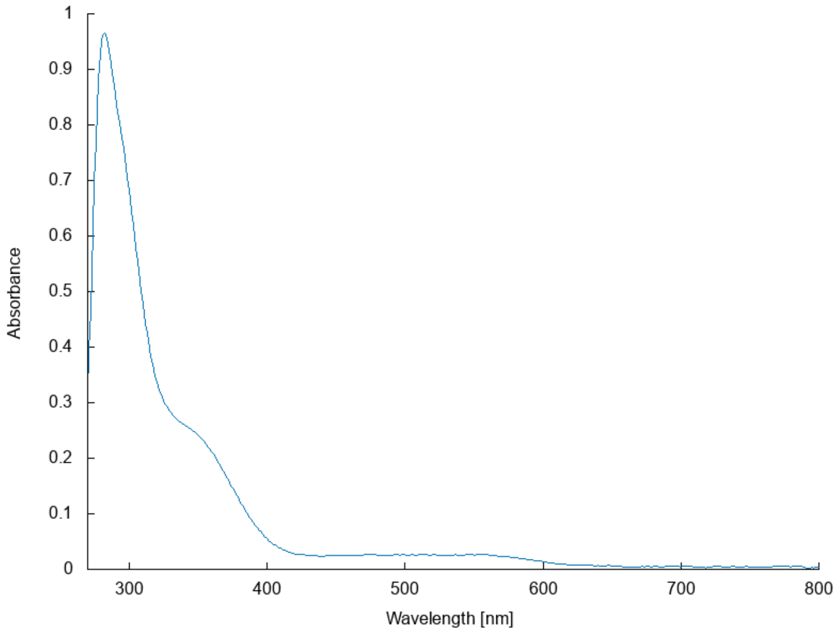


Figure S26. UV/vis spectrum of **7**.

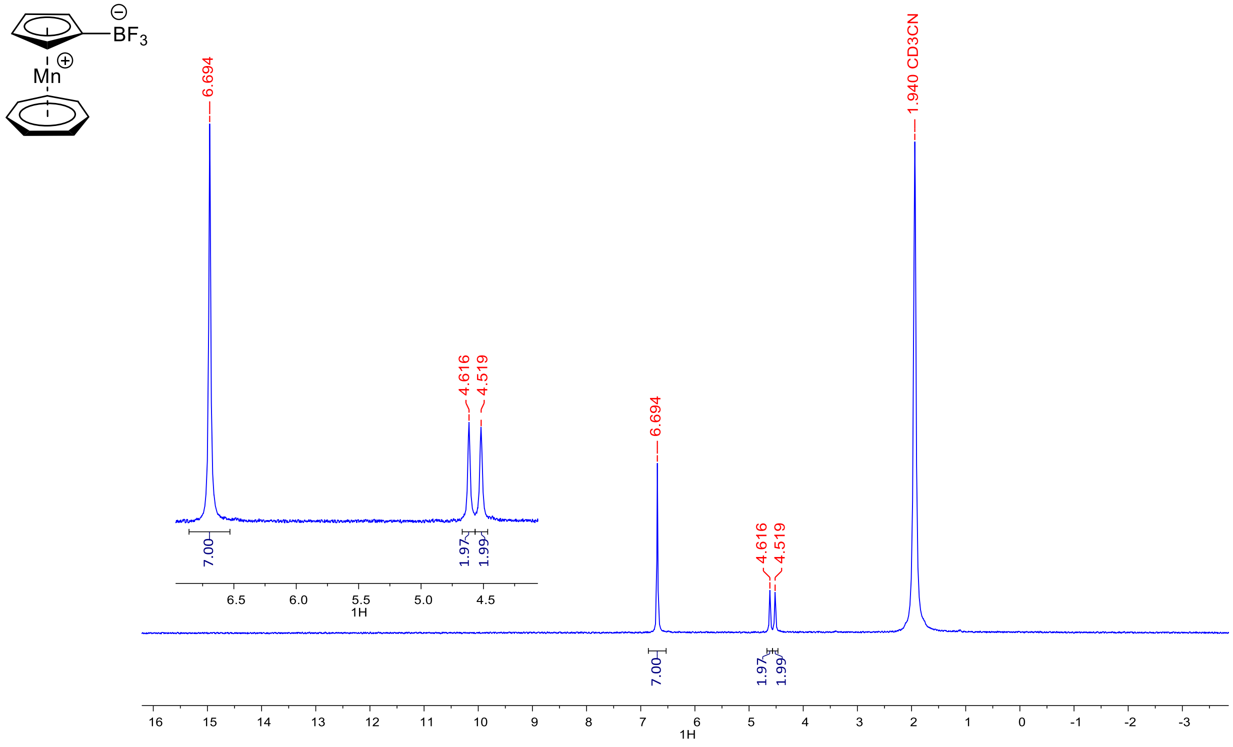


Figure S27. $^{1}$H-NMR spectrum of **7**.

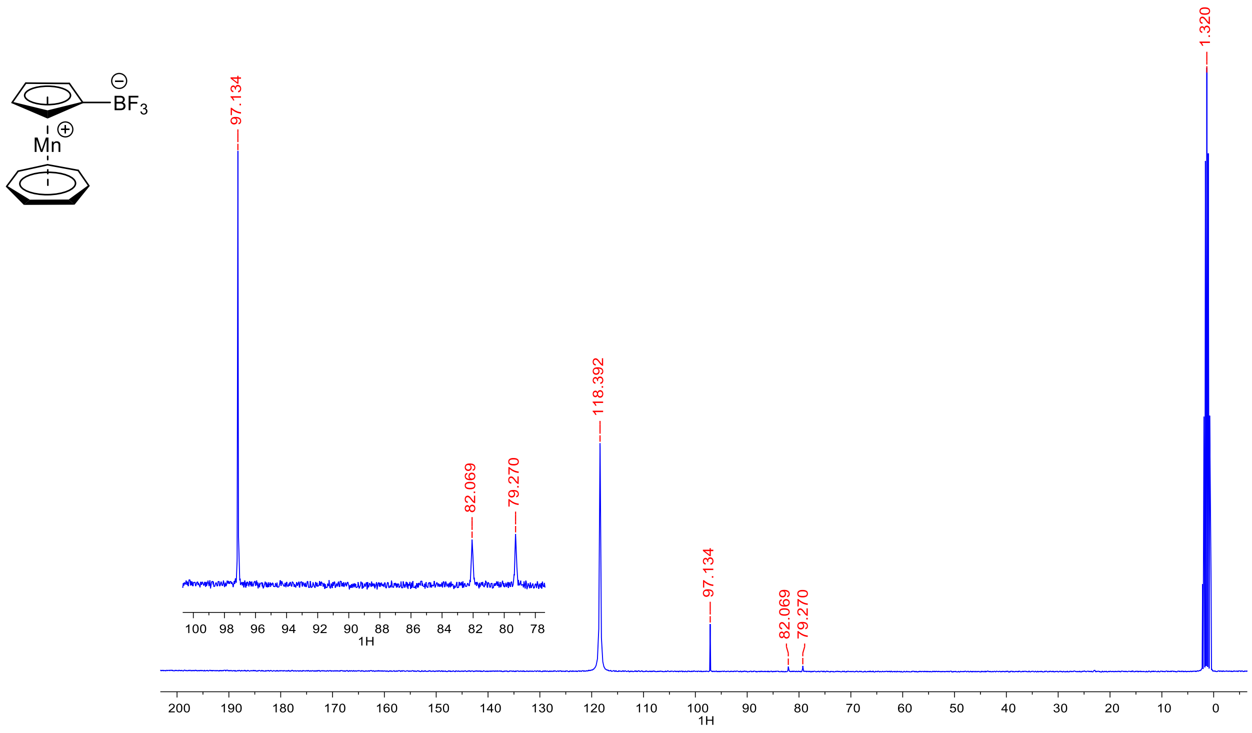


Figure S28. $^{13}$C-NMR spectrum of **7**.

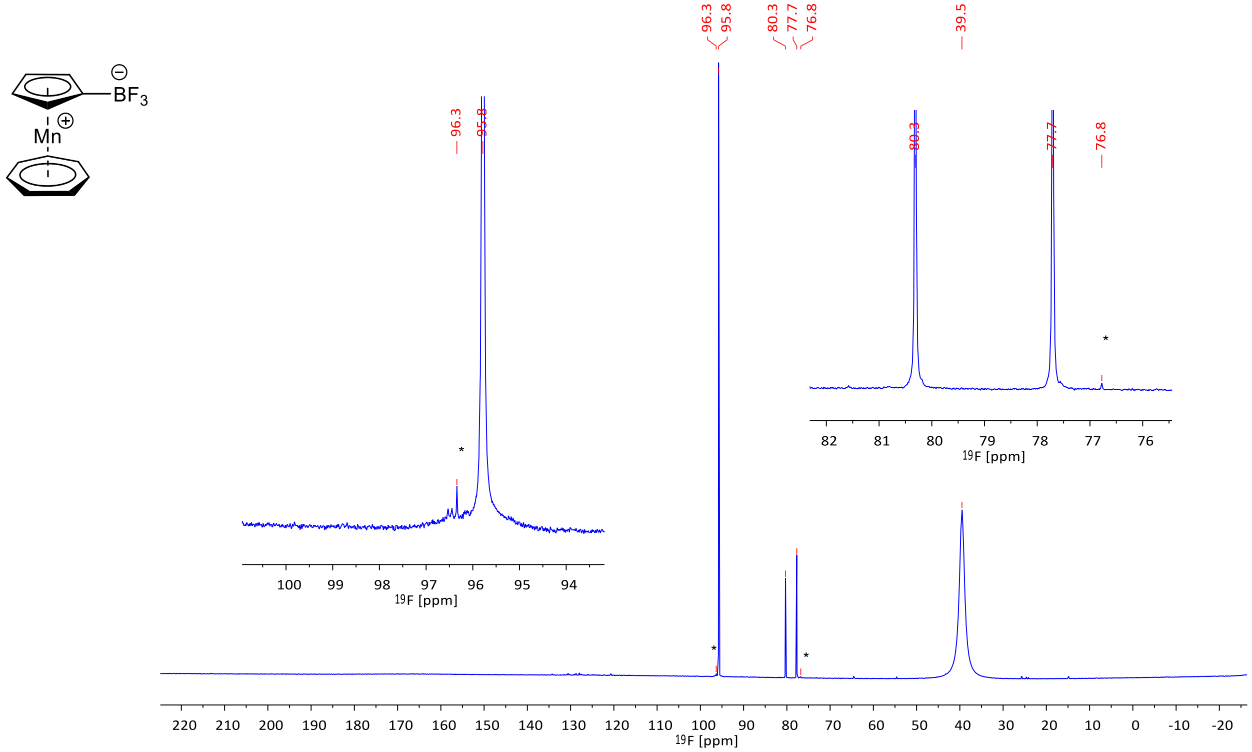


Figure S29. $^{13}C$-NMR spectrum of a saturated solution of **7** in DMSO (marked: traces of unsubstituted tromancenium).

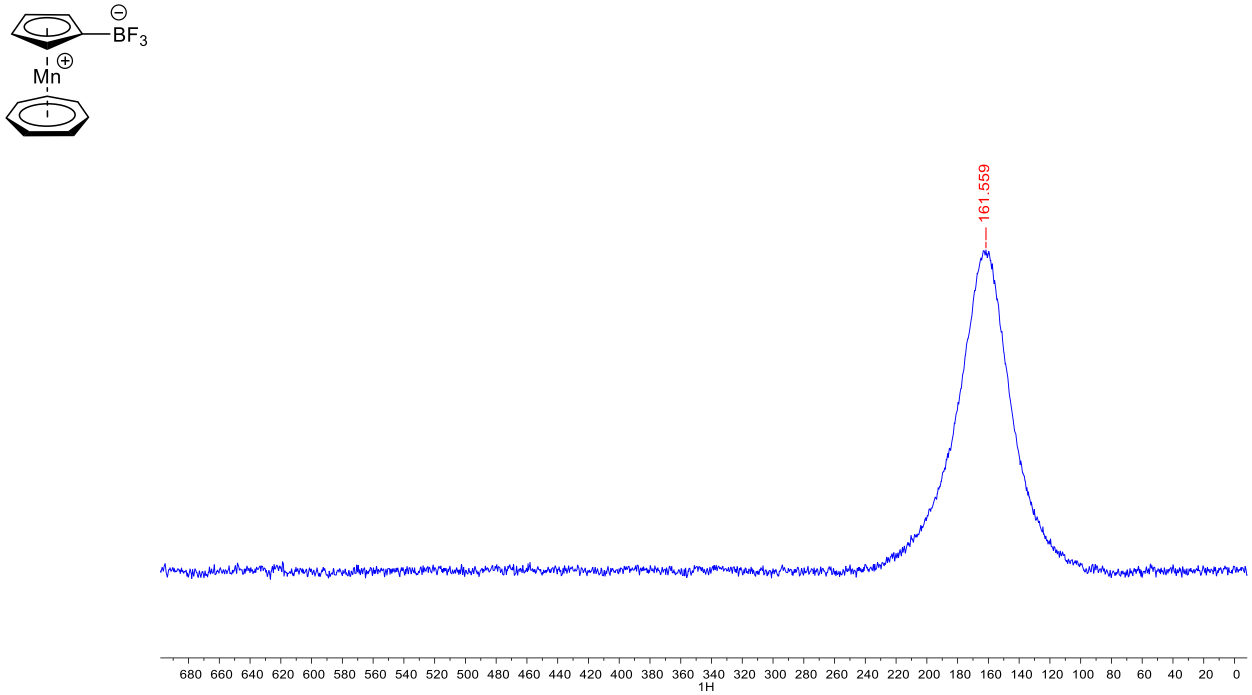


Figure S30. $^{55}Mn$-NMR spectrum of **7**.

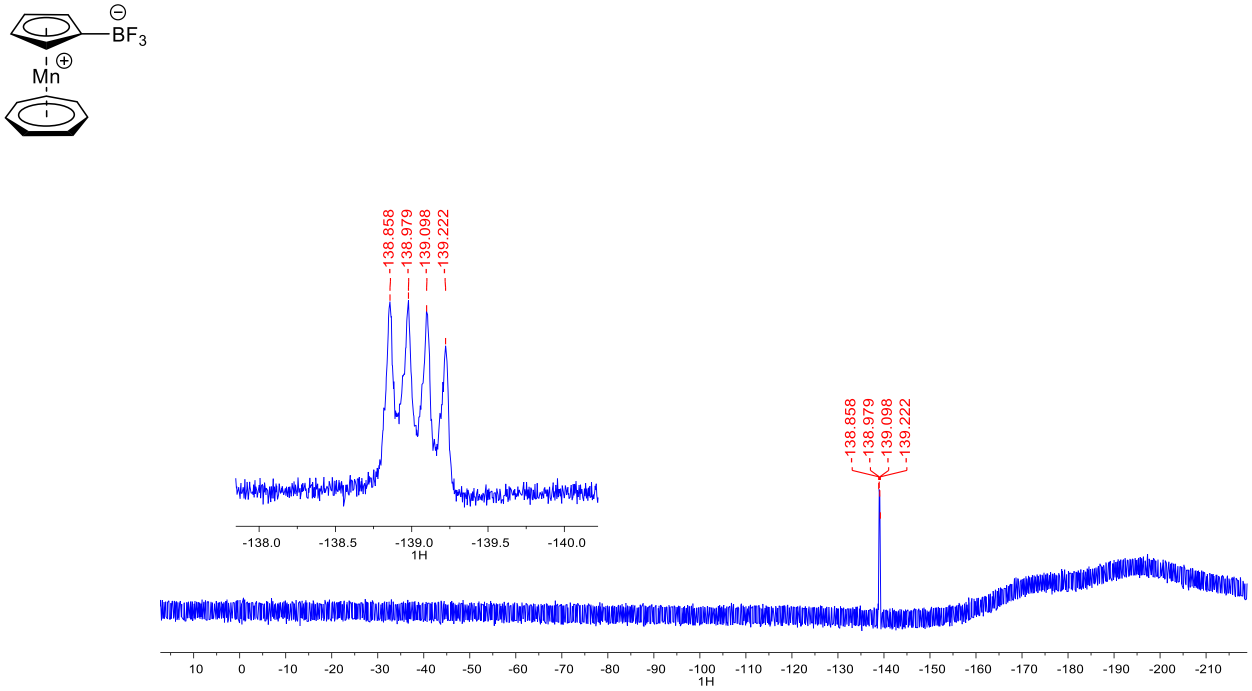


Figure S31. $^{19}$F-NMR spectrum of **7**.

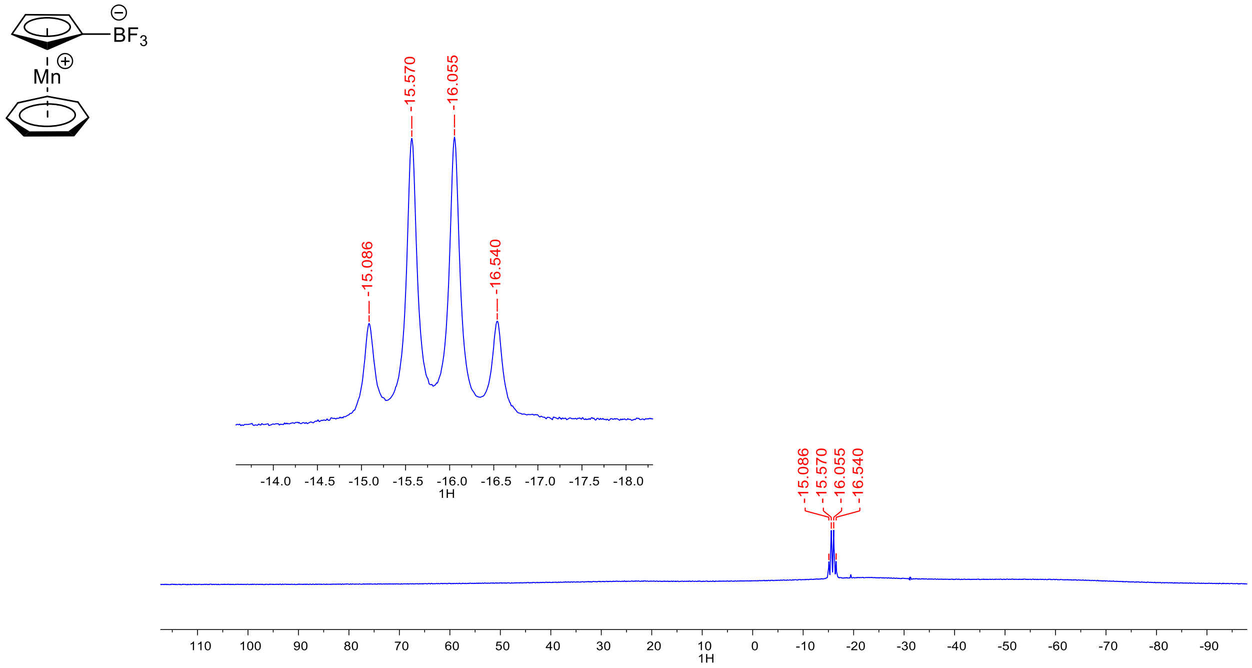


Figure S32. $^{11}$B-NMR spectrum of **7**.

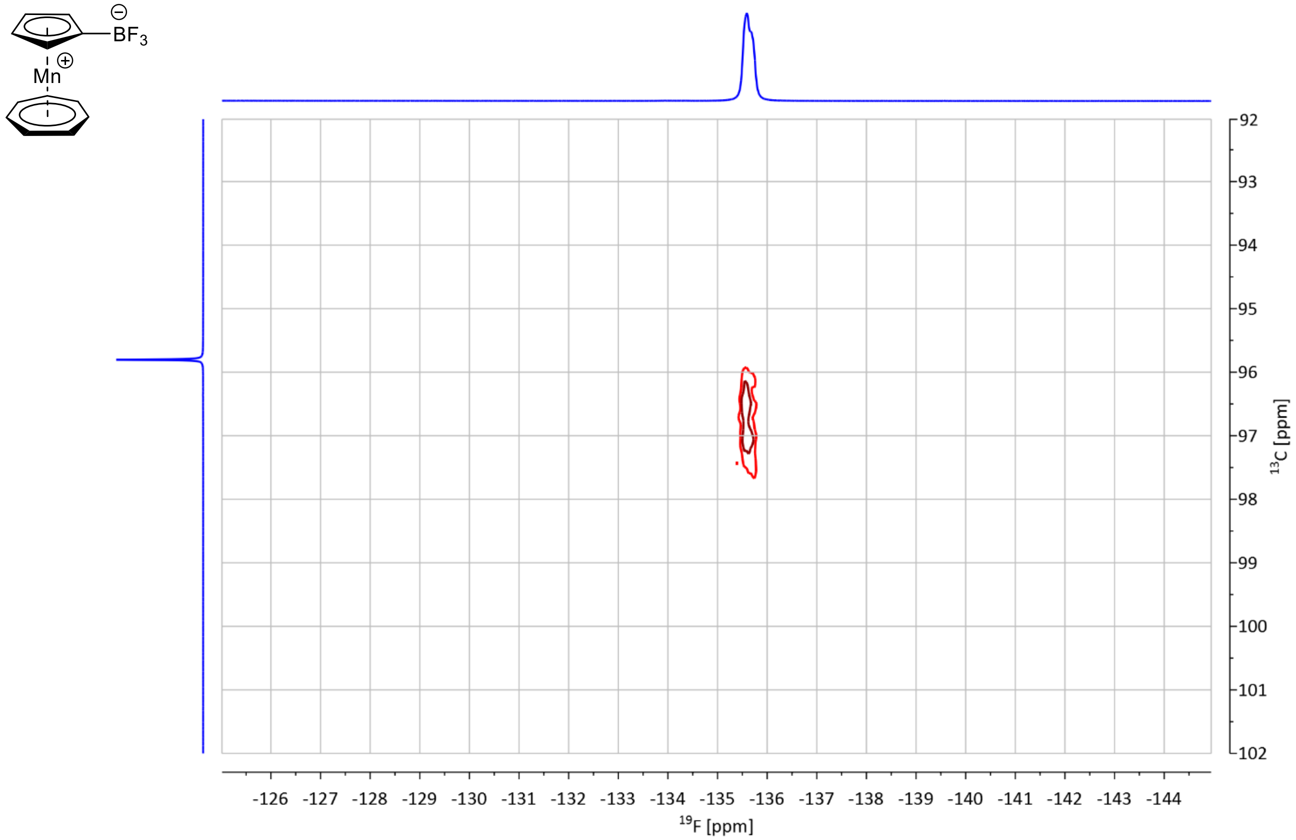


Figure S33. ($^{19}F/^{13}C$)-HSQC NMR spectrum of **7**.

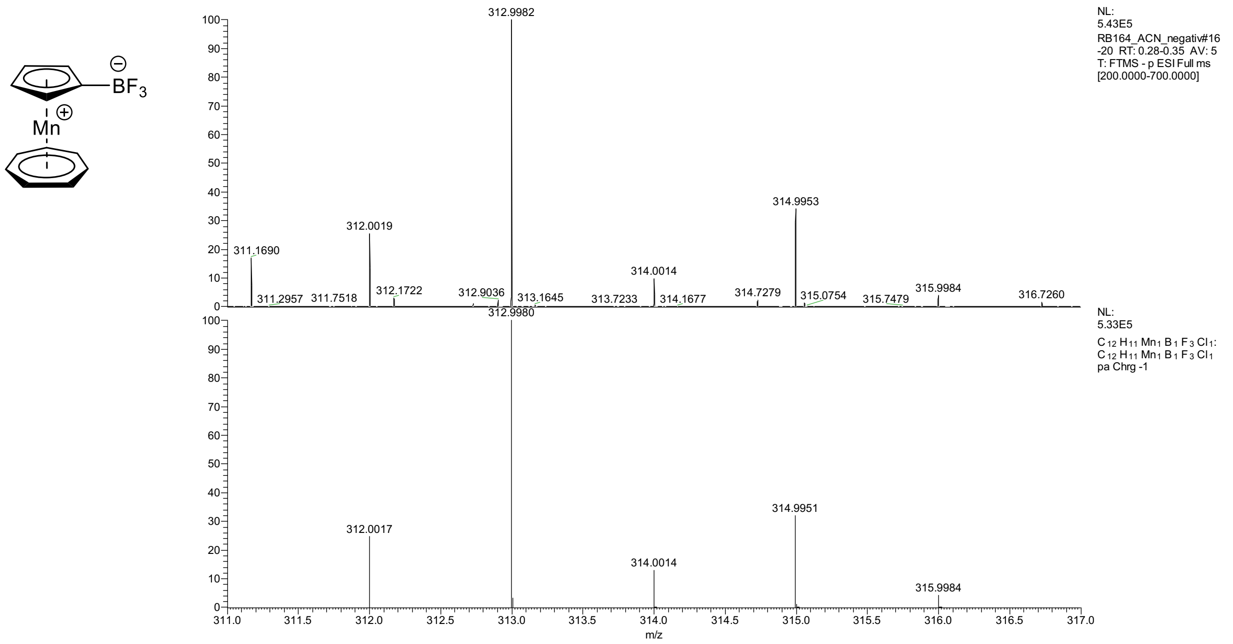


Figure S34. MS (ESI neg, [m/z]; *top*: experimental, *bottom*: simulated) of **7**.

**8-Tromanceniumylboronic acid pinacol ester triflate (8)**

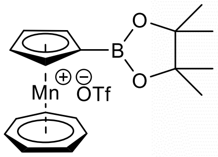


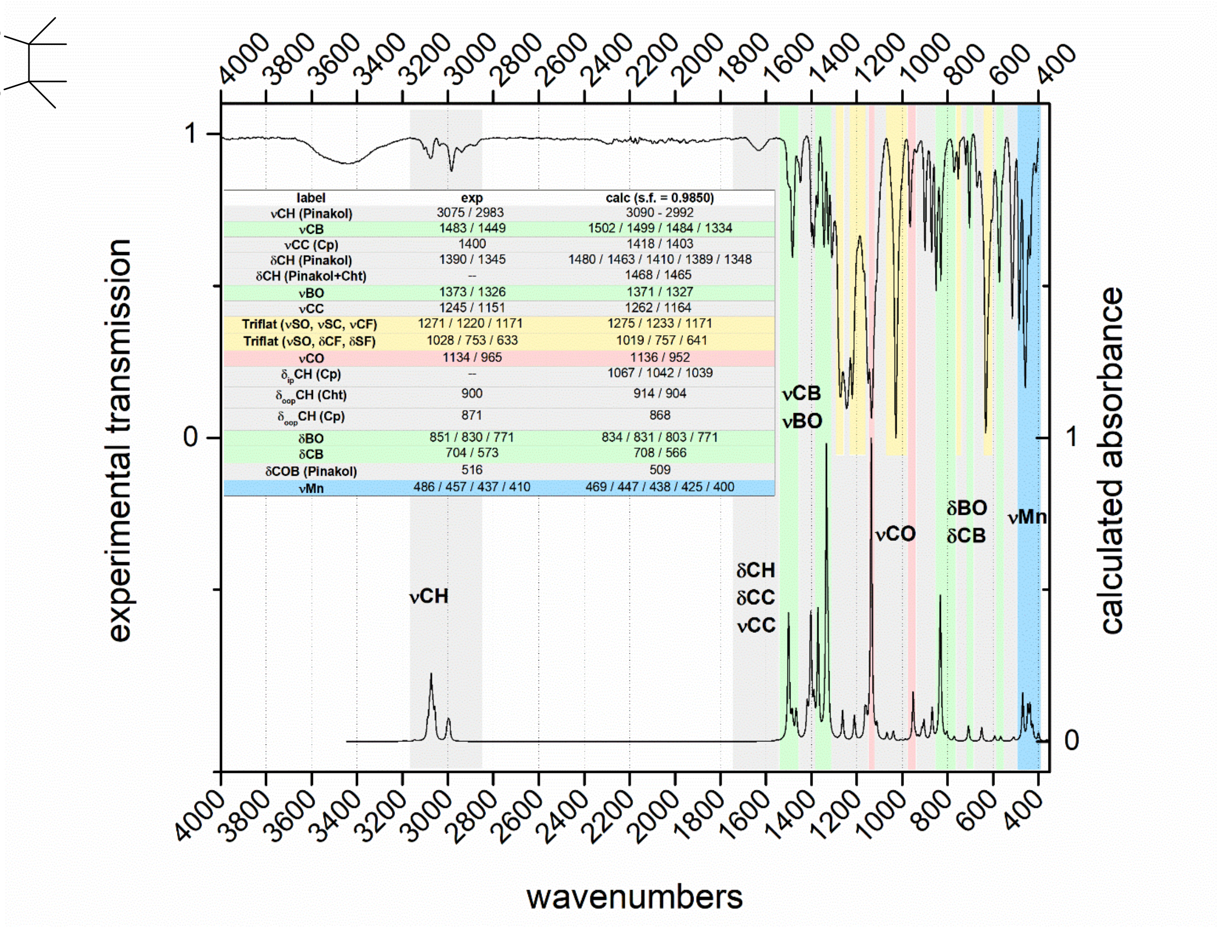


| label | exp | calc (s.f. = 0.9850) |
|---|---|---|
| νCH (Pinakol) | 3075 / 2983 | 3090 - 2992 |
| νCB | 1483 / 1449 | 1502 / 1499 / 1484 / 1334 |
| νCC (Cp) | 1400 | 1418 / 1403 |
| δCH (Pinakol) | 1390 / 1345 | 1480 / 1463 / 1410 / 1389 / 1348 |
| δCH (Pinakol+Cht) | -- | 1468 / 1465 |
| νBO | 1373 / 1326 | 1371 / 1327 |
| νCC | 1245 / 1151 | 1262 / 1164 |
| Triflat (νSO, νSC, νCF) | 1271 / 1220 / 1171 | 1275 / 1233 / 1171 |
| Triflat (νSO, δCF, δSF) | 1028 / 753 / 633 | 1019 / 757 / 641 |
| νCO | 1134 / 965 | 1136 / 952 |
| $\delta_{ip}$CH (Cp) | -- | 1067 / 1042 / 1039 |
| $\delta_{oop}$CH (Cht) | 900 | 914 / 904 |
| $\delta_{oop}$CH (Cp) | 871 | 868 |
| δBO | 851 / 830 / 771 | 834 / 831 / 803 / 771 |
| δCB | 704 / 573 | 708 / 566 |
| δCOB (Pinakol) | 516 | 509 |
| νMn | 486 / 457 / 437 / 410 | 469 / 447 / 438 / 425 / 400 |

Figure S35. IR spectrum of **8**.

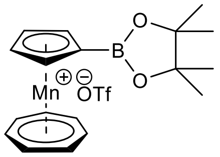


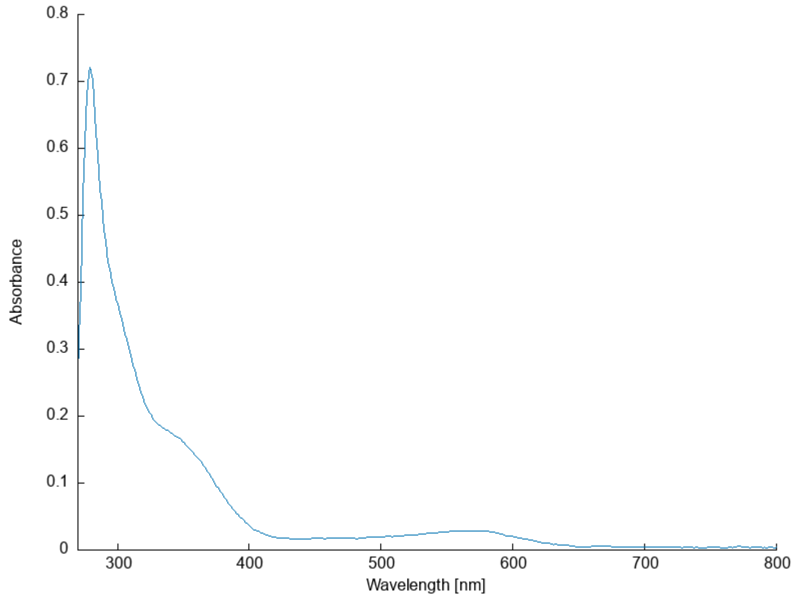


Figure S36. UV/vis spectrum **8**.

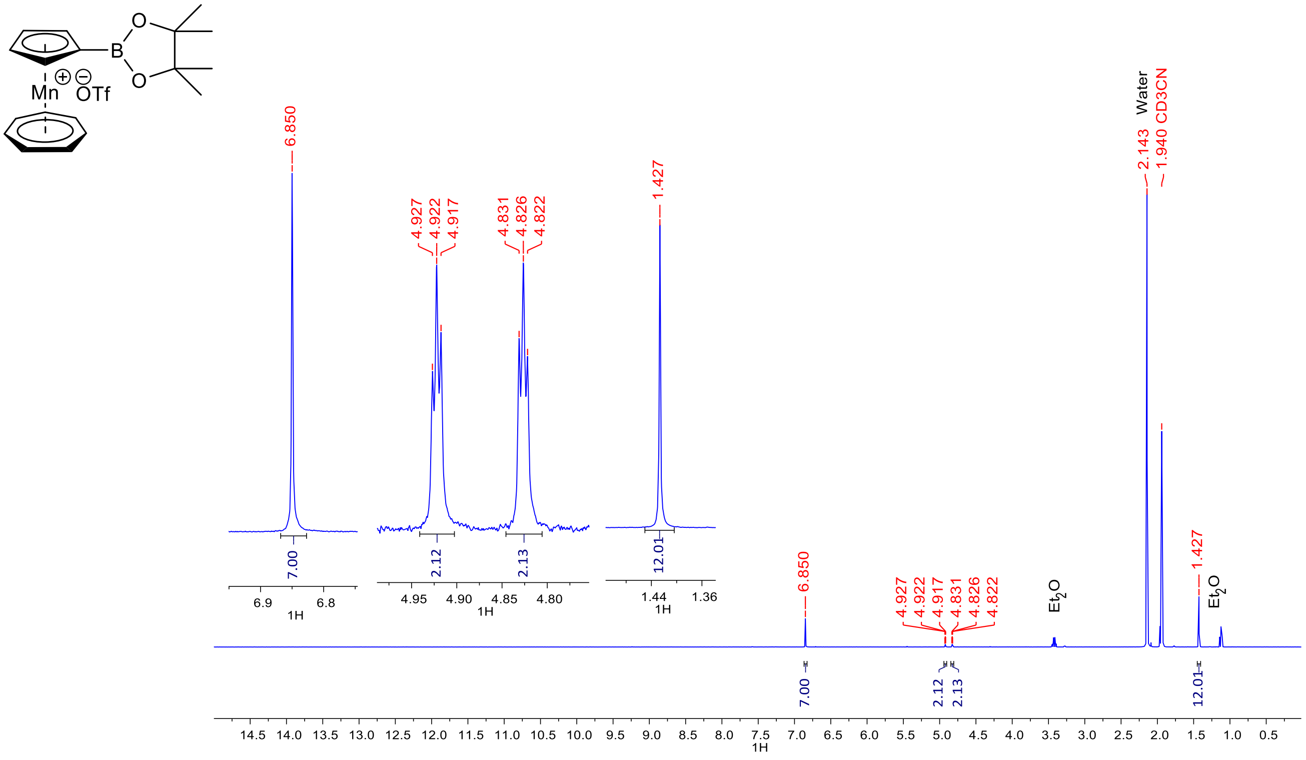

Figure S37. $^{1}$H-NMR spectrum of **8**.

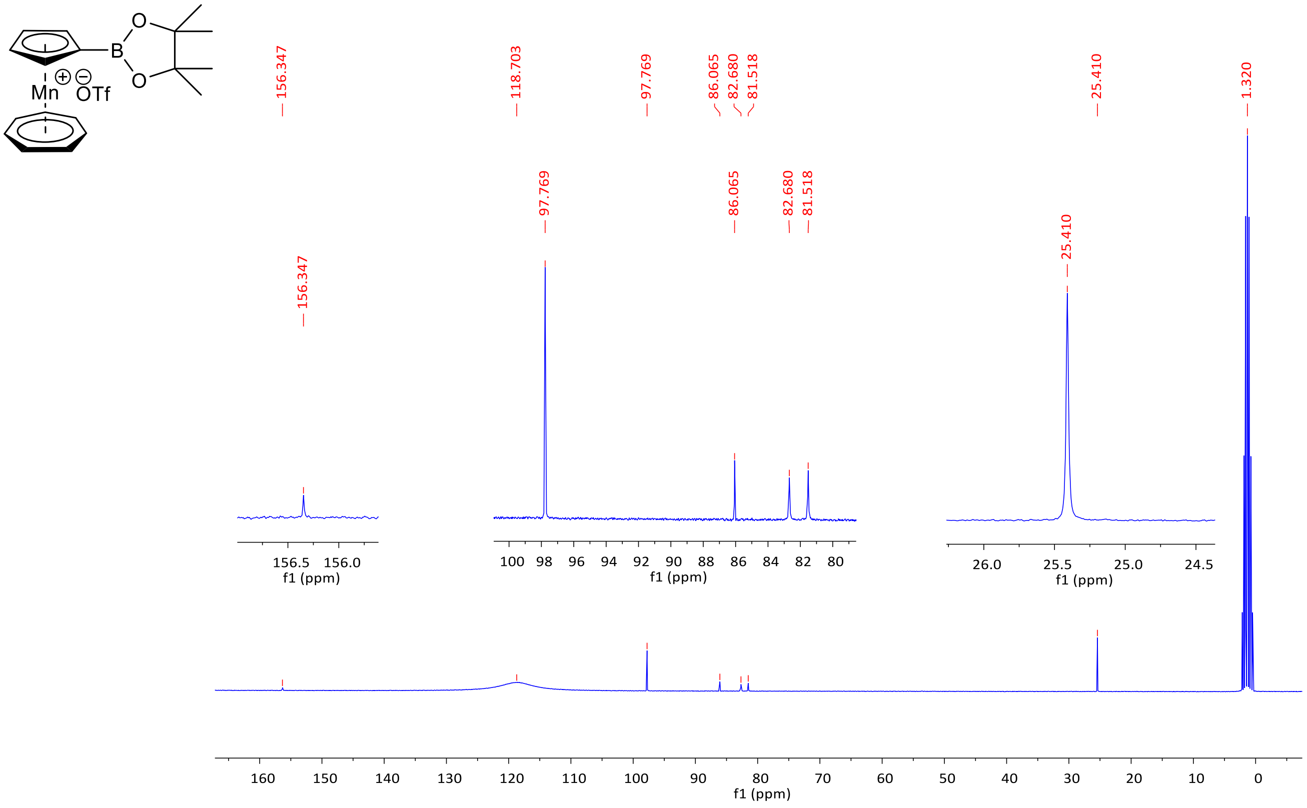

Figure S38. $^{13}$C-NMR spectrum of **8**.

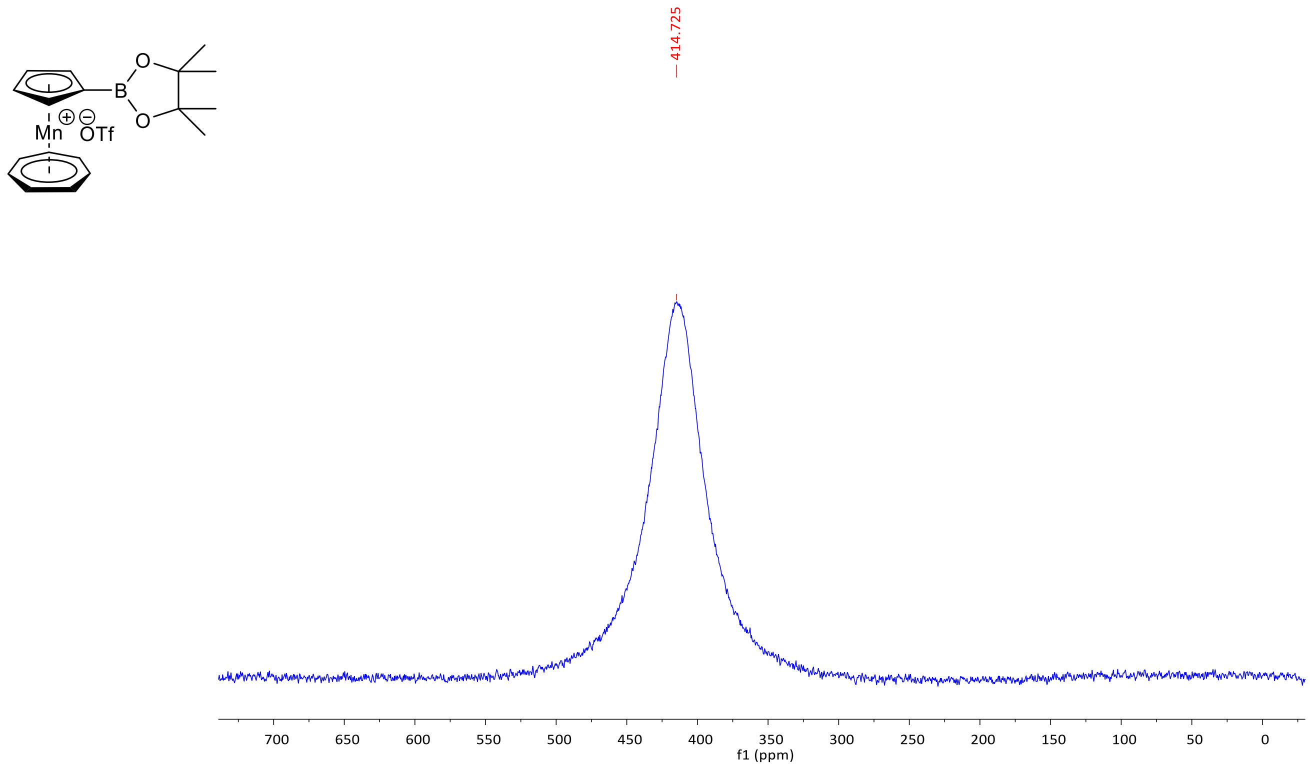


Figure S39. [55]Mn-NMR spectrum of **8**.

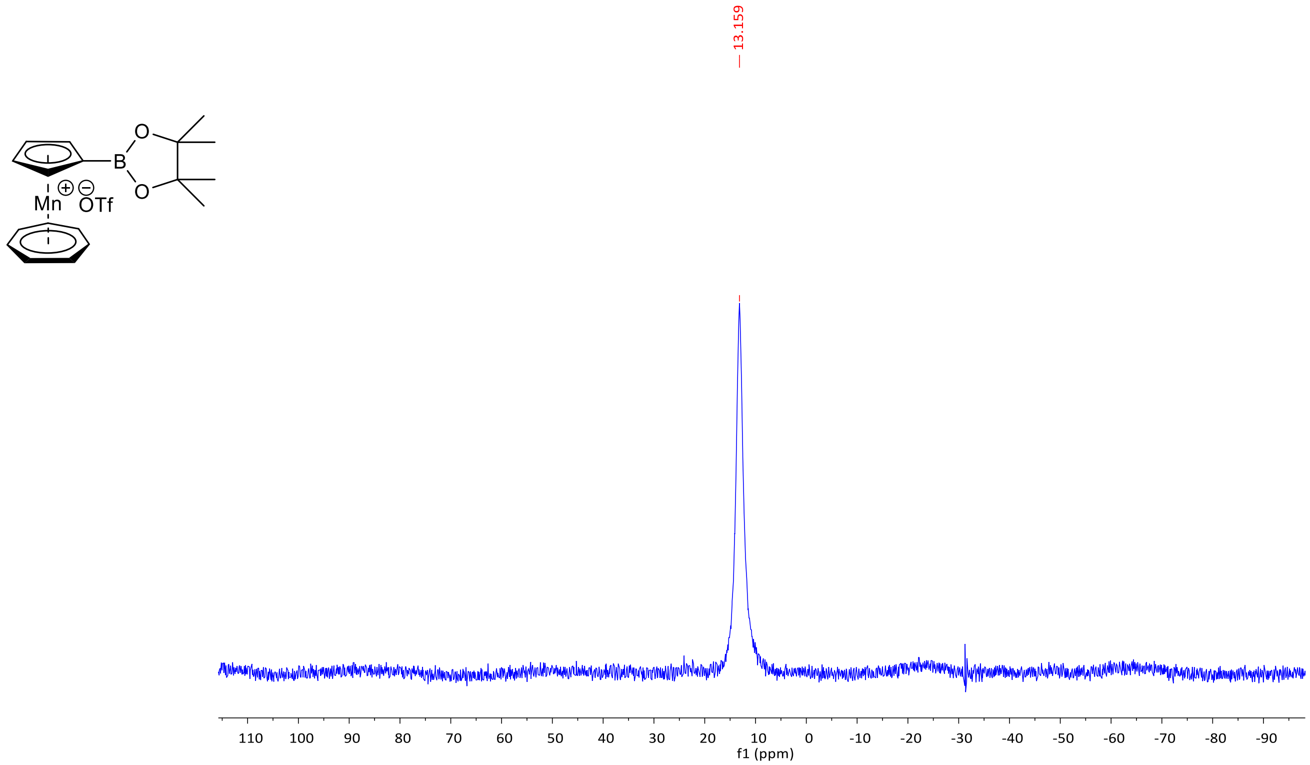


Figure S40. $^{11}$B-NMR spectrum of **8**.

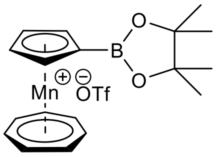

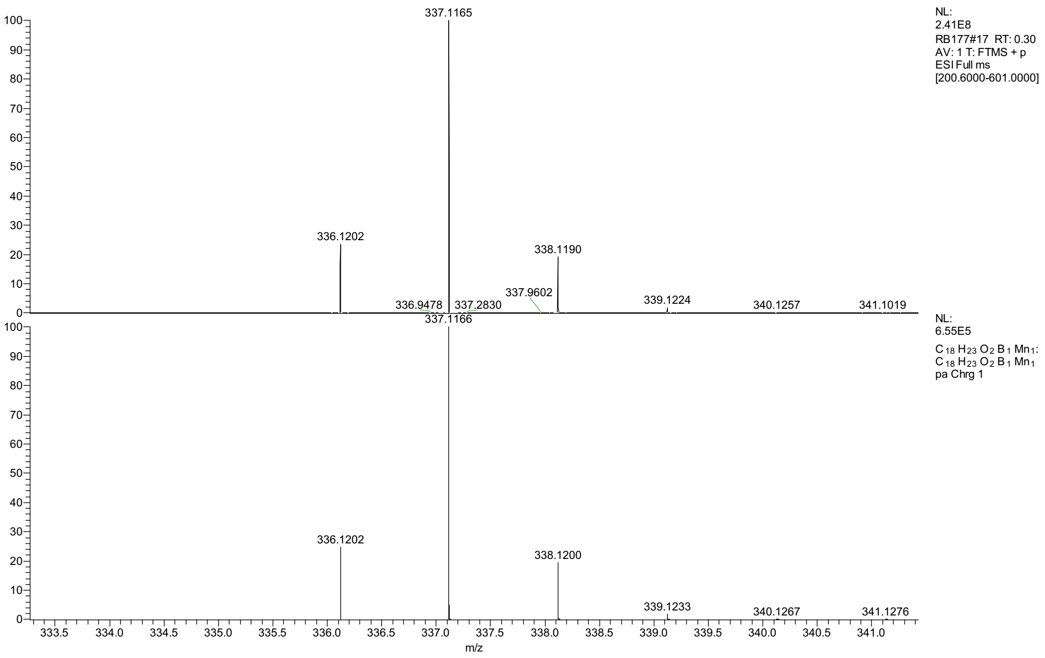


Figure S41. MS (ESI pos, [m/z]; *top*: experimental, *bottom*: simulated) of **8**.

**8-Tromanceniumylboronic acid triflate (9)**

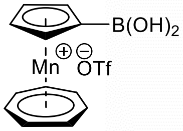


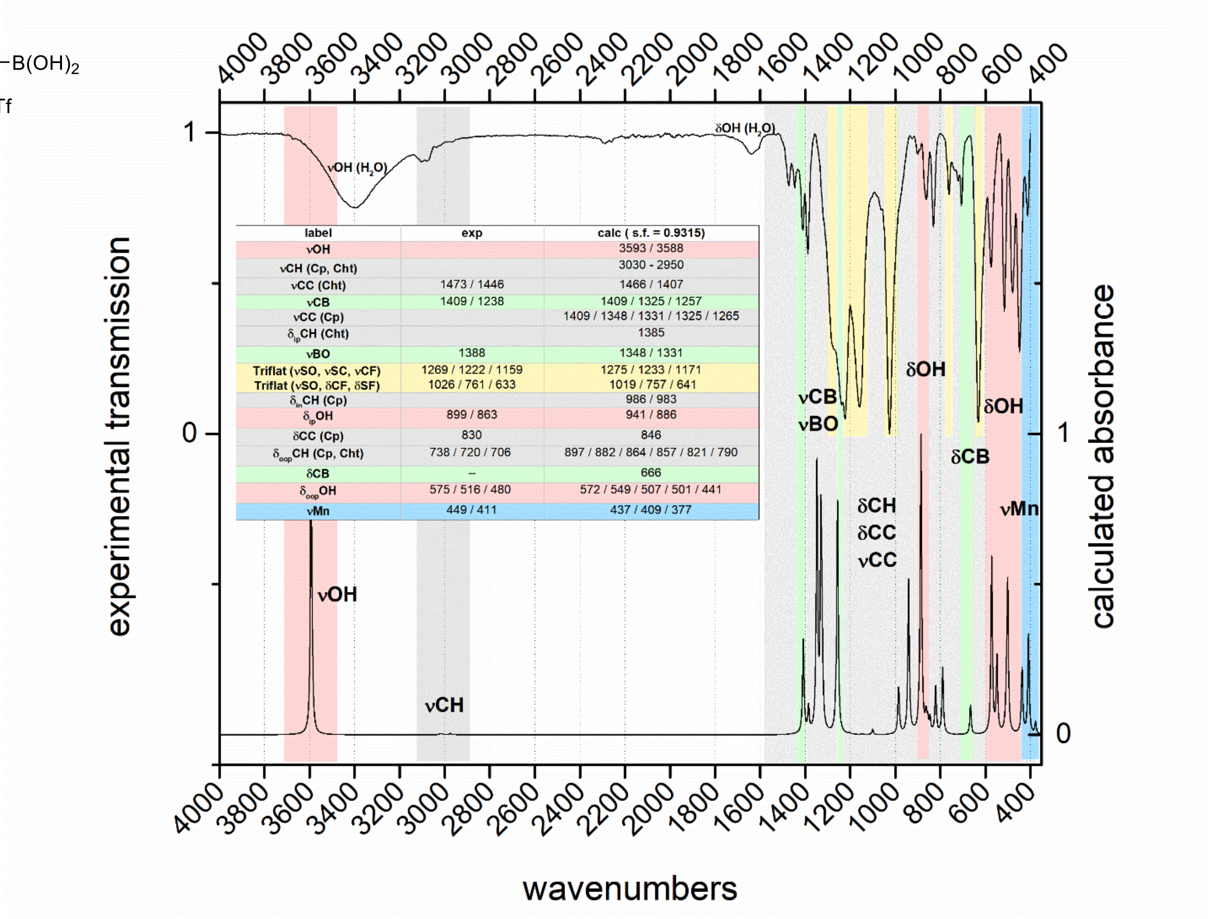


| label | exp | calc ( s.f. = 0.9315) |
|---|---|---|
| νOH | | 3593 / 3588 |
| νCH (Cp, Cht) | | 3030 - 2950 |
| νCC (Cht) | 1473 / 1446 | 1466 / 1407 |
| νCB | 1409 / 1238 | 1409 / 1325 / 1257 |
| νCC (Cp) | | 1409 / 1348 / 1331 / 1325 / 1265 |
| $\delta_{ip}$CH (Cht) | | 1385 |
| νBO | 1388 | 1348 / 1331 |
| Triflat (νSO, νSC, νCF) | 1269 / 1222 / 1159 | 1275 / 1233 / 1171 |
| Triflat (νSO, δCF, δSF) | 1026 / 761 / 633 | 1019 / 757 / 641 |
| $\delta_{in}$CH (Cp) | | 986 / 983 |
| $\delta_{ip}$OH | 899 / 863 | 941 / 886 |
| δCC (Cp) | 830 | 846 |
| $\delta_{oop}$CH (Cp, Cht) | 738 / 720 / 706 | 897 / 882 / 864 / 857 / 821 / 790 |
| δCB | – | 666 |
| $\delta_{oop}$OH | 575 / 516 / 480 | 572 / 549 / 507 / 501 / 441 |
| νMn | 449 / 411 | 437 / 409 / 377 |

Figure S42. IR spectrum of **9**.

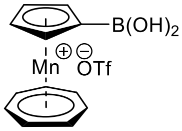


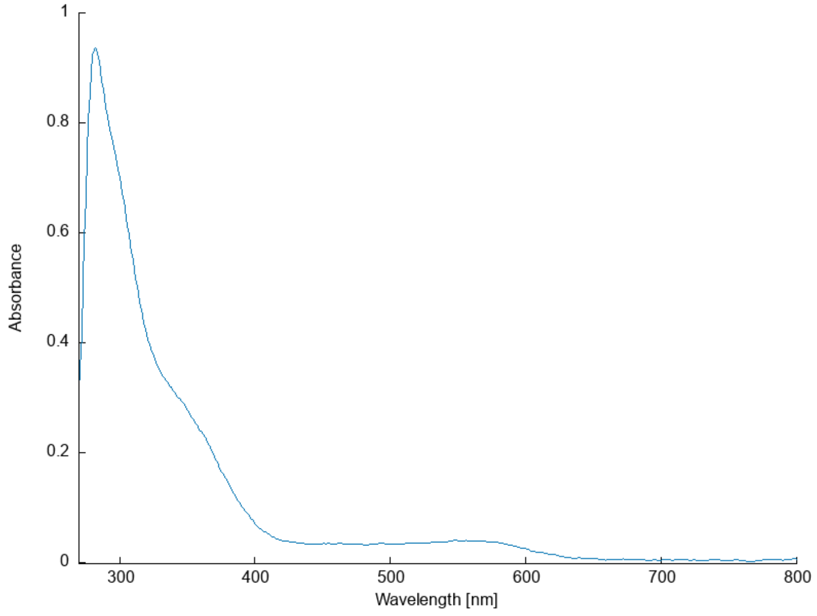


Figure S43. UV/vis spectrum of **9**.

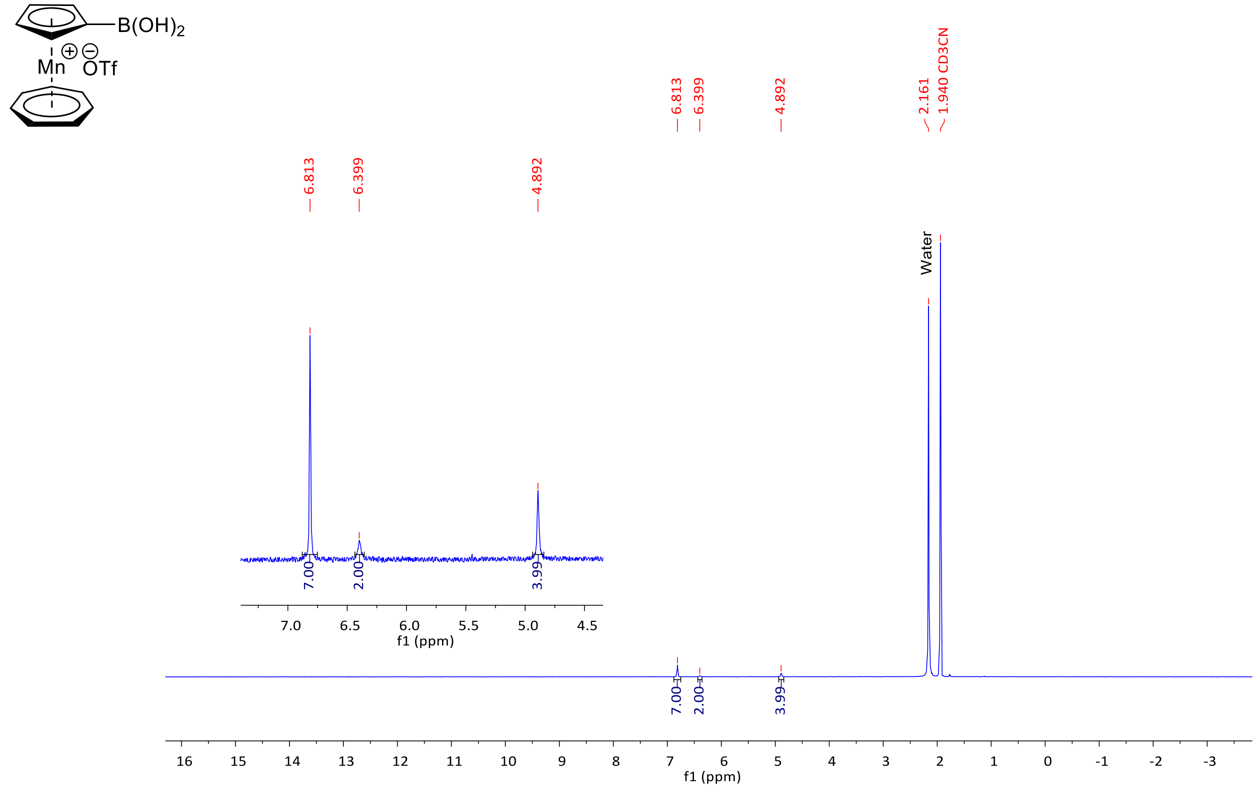


Figure S44. $^{1}$H-NMR spectrum of **9**.

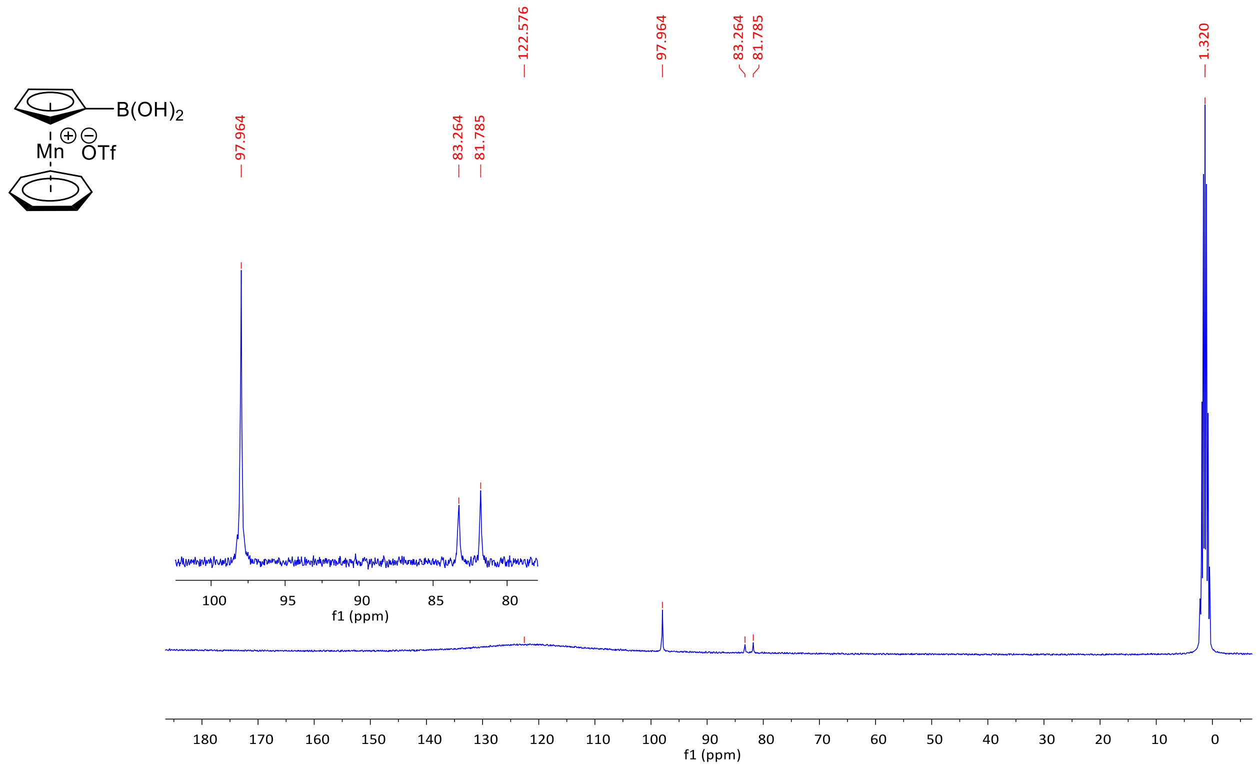


Figure S45. $^{13}$C-NMR spectrum of **9**.

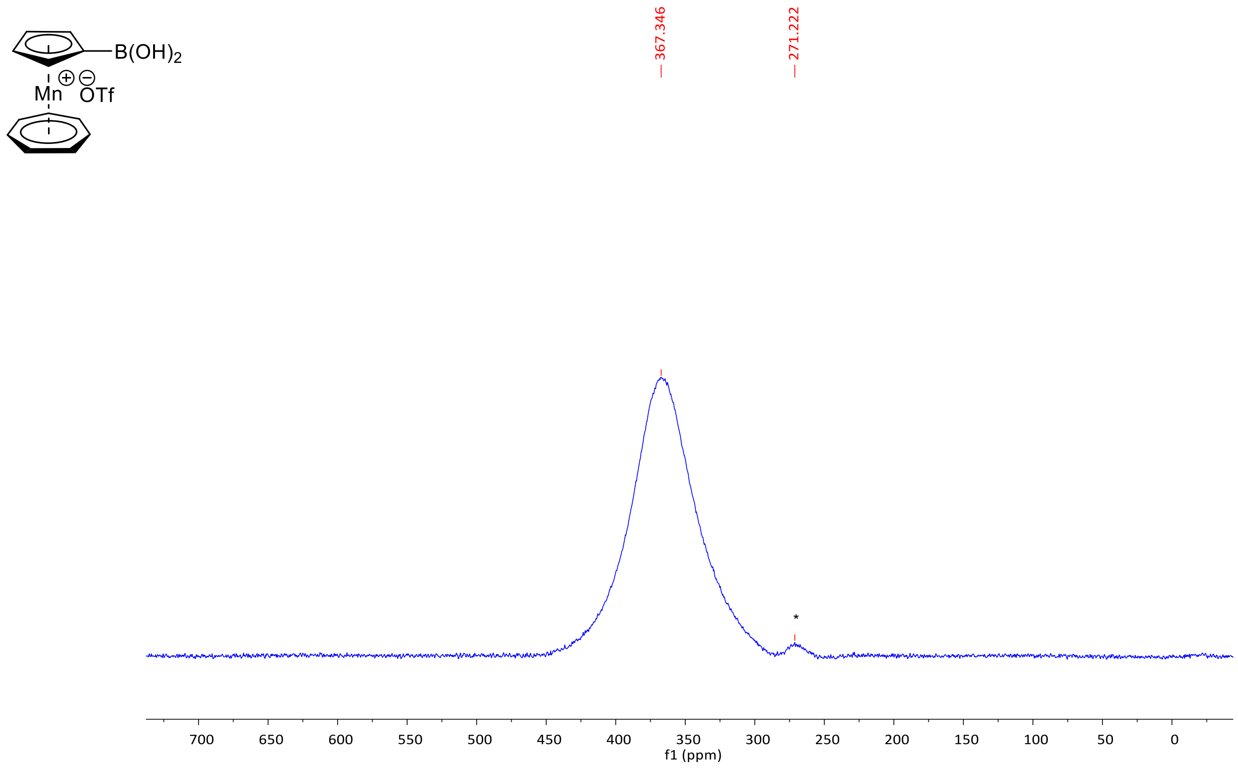


Figure S46. $^{55}$Mn-NMR spectrum of **9** (*marked:* traces (~5%) of unsubstituted tromancenium).

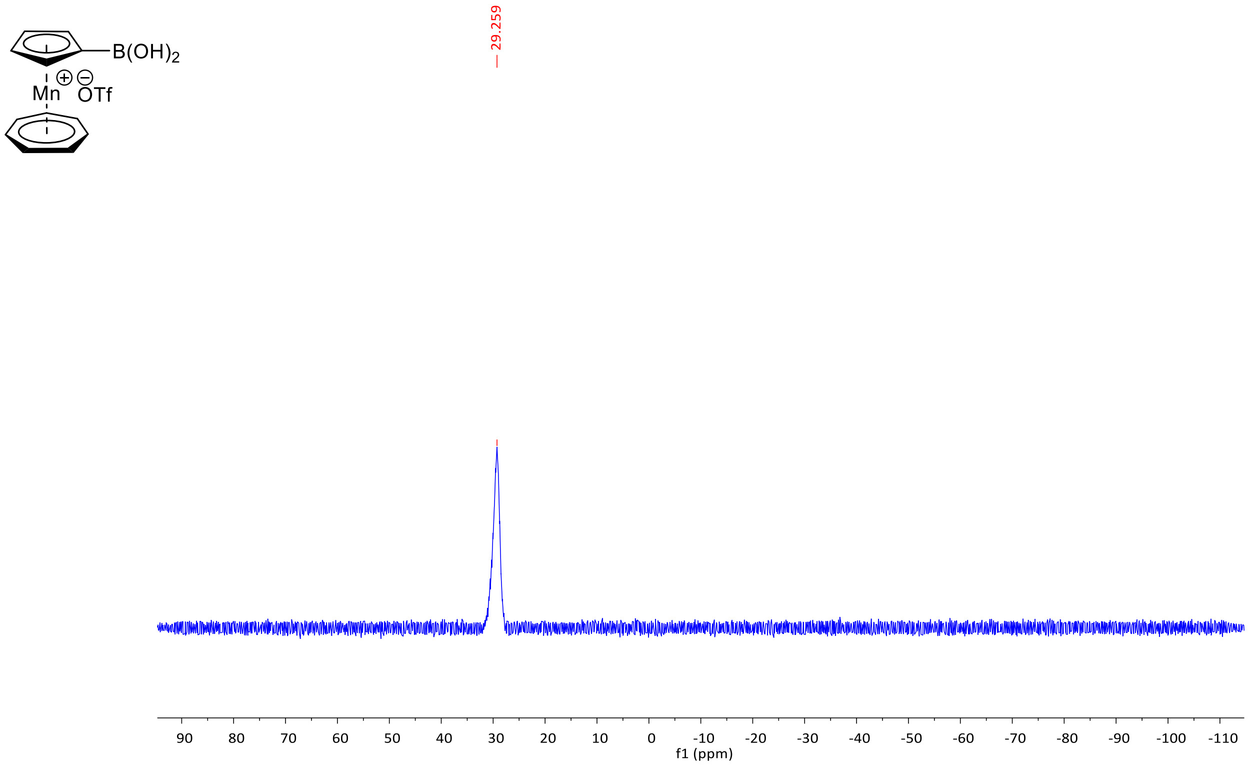


Figure S47. $^{11}$B-NMR spectrum of **9.**

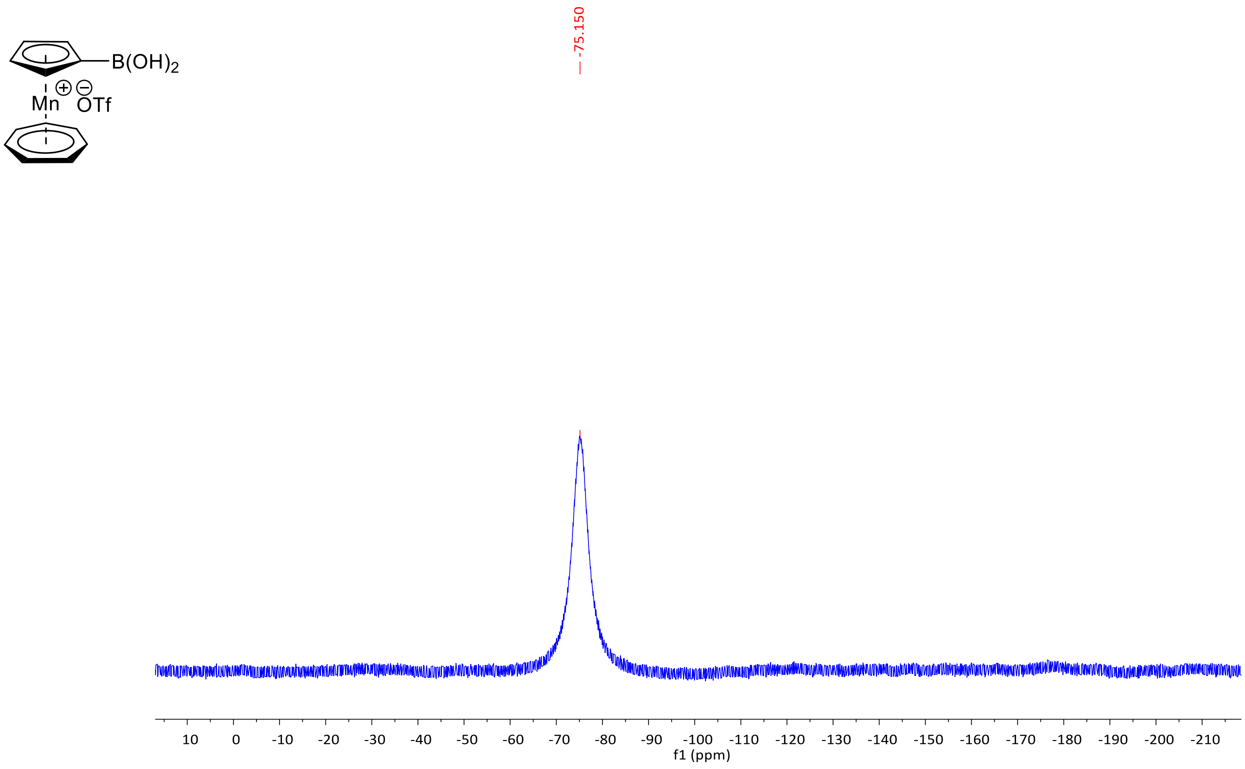


Figure S48. $^{19}$F-NMR spectrum of **9**.

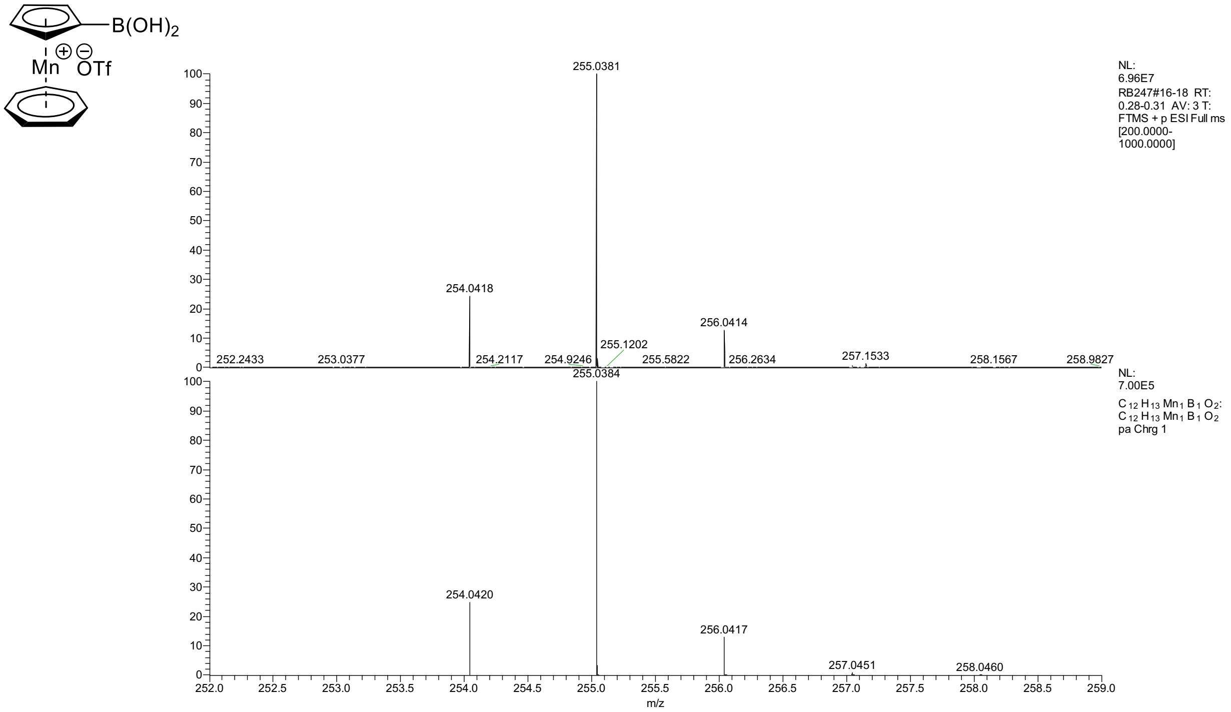


Figure S49. MS (ESI pos, [m/z]; *top*: experimental, *bottom*: simulated) of **9**.

## 8-Aminotromancenium triflate (10)

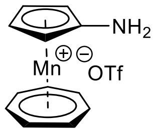


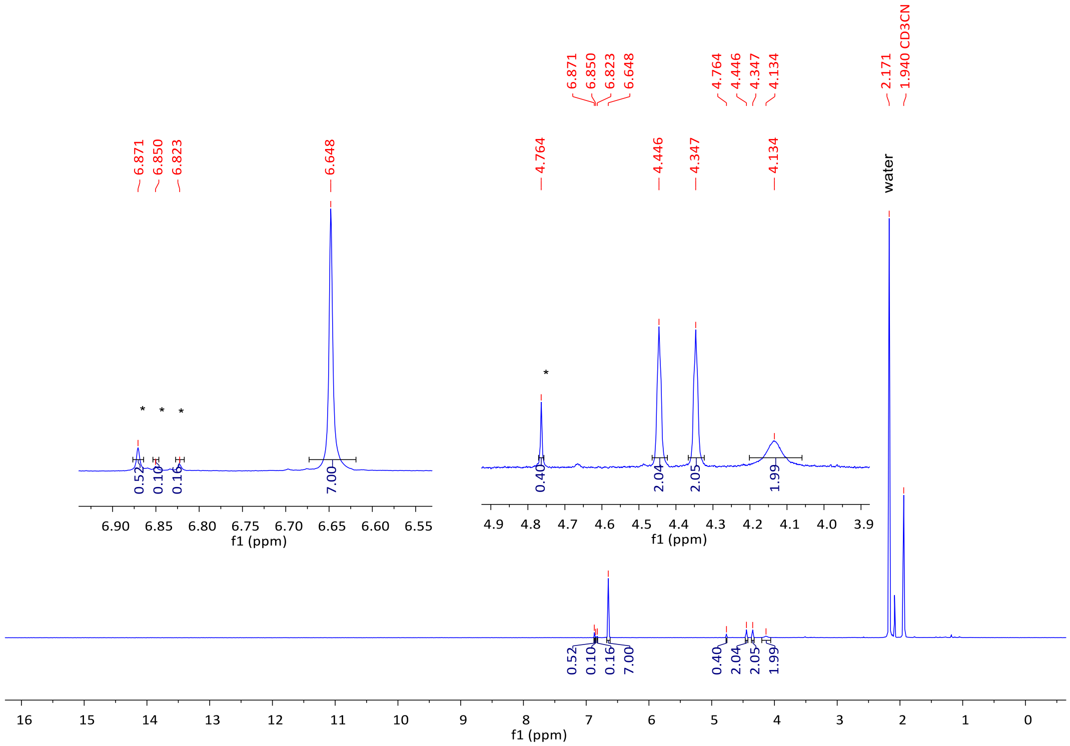


Figure S50. $^1$H-NMR spectrum of **10** (marked: traces of unsubstituted tromancenium, educt **8** and an unknown compound).

**8-Chlorotromancenium triflate (12)**

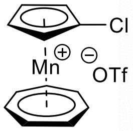


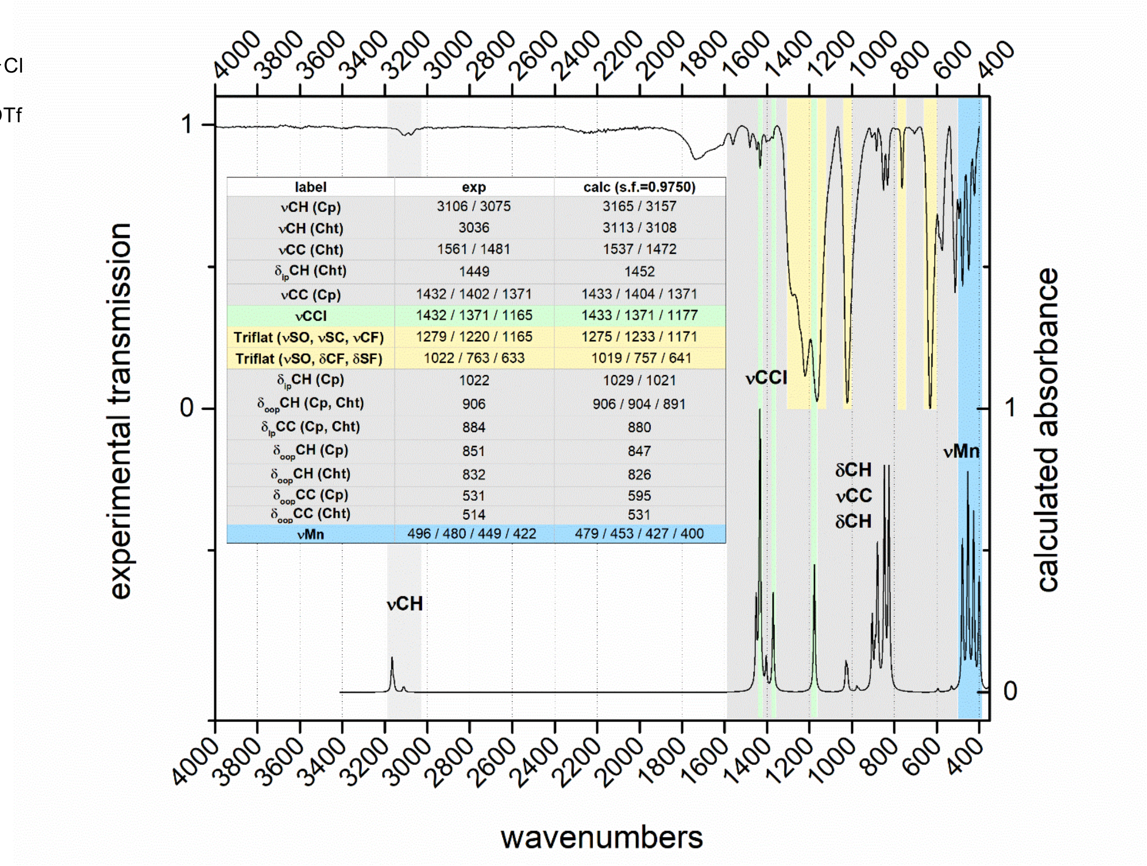


| label | exp | calc (s.f.=0.9750) |
|---|---|---|
| νCH (Cp) | 3106 / 3075 | 3165 / 3157 |
| νCH (Cht) | 3036 | 3113 / 3108 |
| νCC (Cht) | 1561 / 1481 | 1537 / 1472 |
| $\delta_{ip}$CH (Cht) | 1449 | 1452 |
| νCC (Cp) | 1432 / 1402 / 1371 | 1433 / 1404 / 1371 |
| νCCl | 1432 / 1371 / 1165 | 1433 / 1371 / 1177 |
| Triflat (νSO, νSC, νCF) | 1279 / 1220 / 1165 | 1275 / 1233 / 1171 |
| Triflat (νSO, δCF, δSF) | 1022 / 763 / 633 | 1019 / 757 / 641 |
| $\delta_{ip}$CH (Cp) | 1022 | 1029 / 1021 |
| $\delta_{oop}$CH (Cp, Cht) | 906 | 906 / 904 / 891 |
| $\delta_{ip}$CC (Cp, Cht) | 884 | 880 |
| $\delta_{oop}$CH (Cp) | 851 | 847 |
| $\delta_{oop}$CH (Cht) | 832 | 826 |
| $\delta_{oop}$CC (Cp) | 531 | 595 |
| $\delta_{oop}$CC (Cht) | 514 | 531 |
| νMn | 496 / 480 / 449 / 422 | 479 / 453 / 427 / 400 |

Figure S51. IR spectrum of **12**.

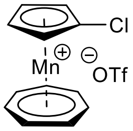


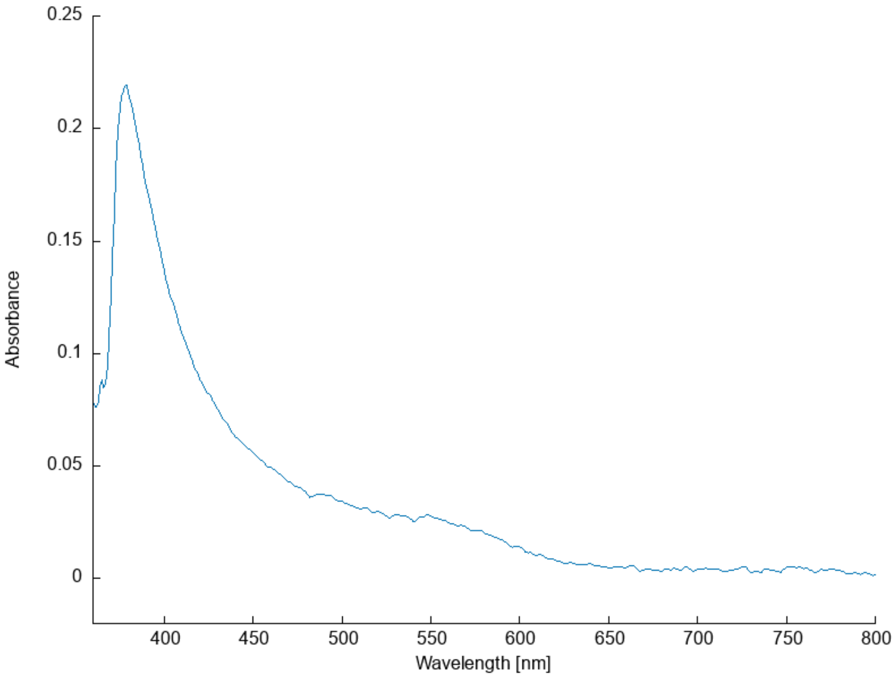


Figure S52. UV/vis spectrum **12**.

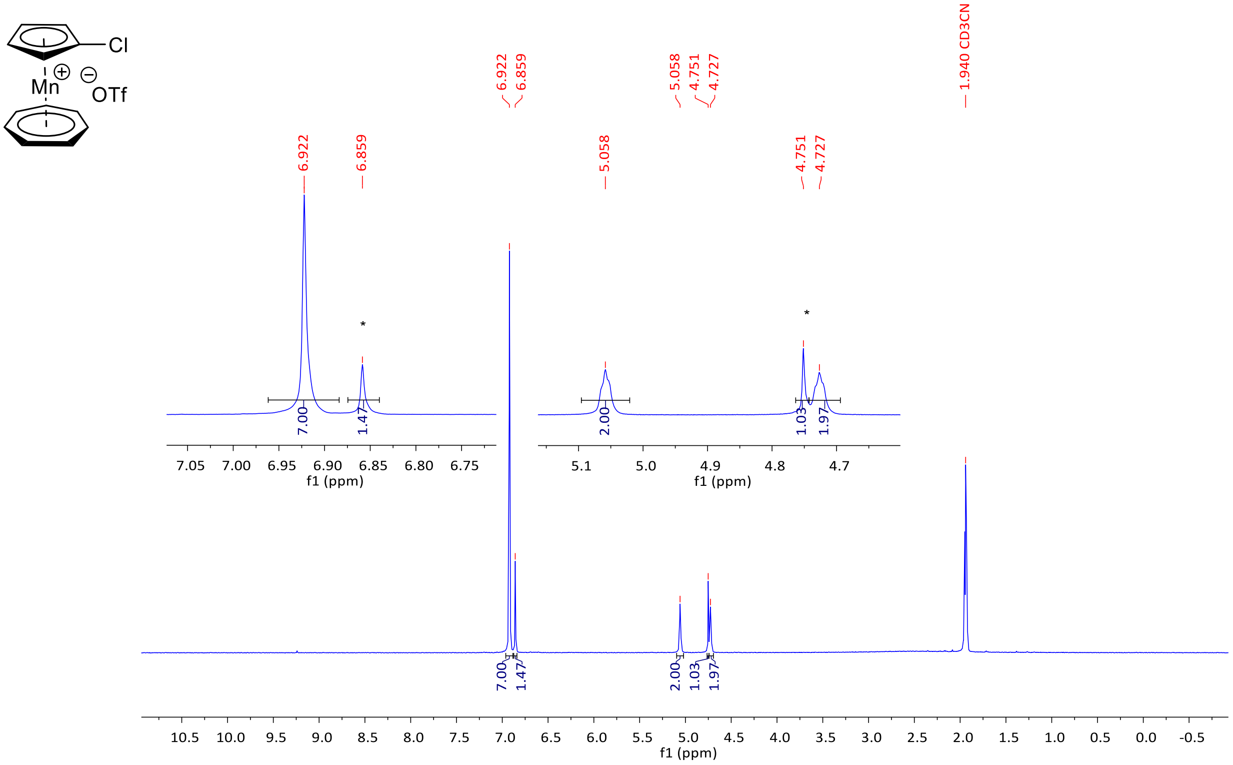


Figure S53. $^{1}$H-NMR spectrum of **12** (marked: traces (~15%) of unsubstituted tromancenium).

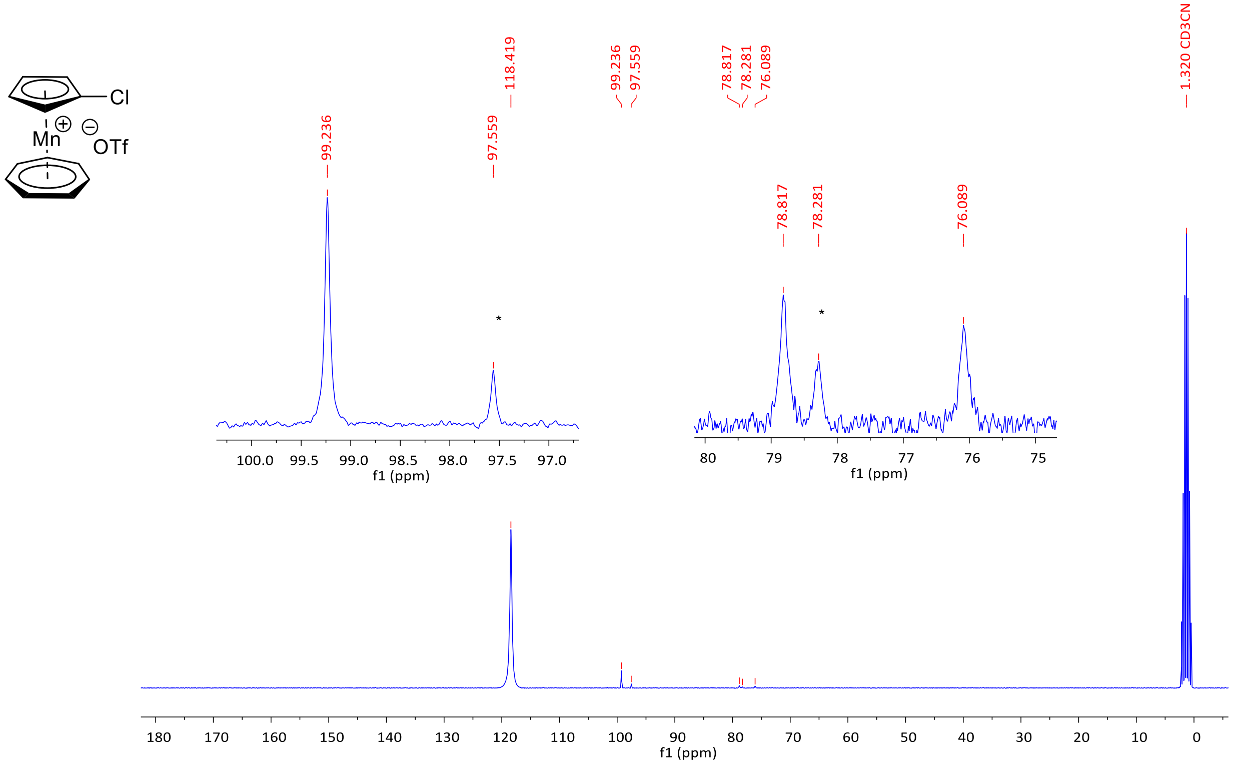


Figure S54. $^{13}$C-NMR spectrum of **12** (marked: traces (~15%) of unsubstituted tromancenium).

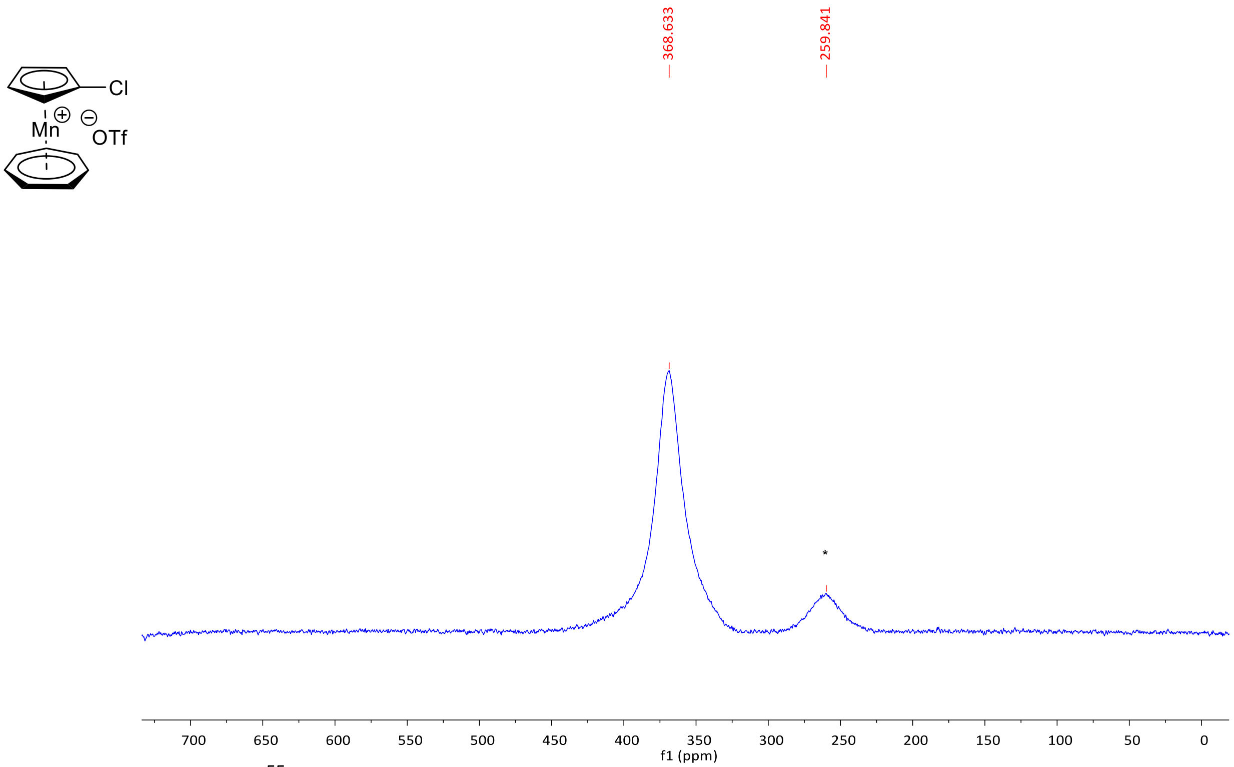


Figure S55. $^{55}$Mn-NMR spectrum of **12** (marked: traces (~15%) of unsubstituted tromancenium).

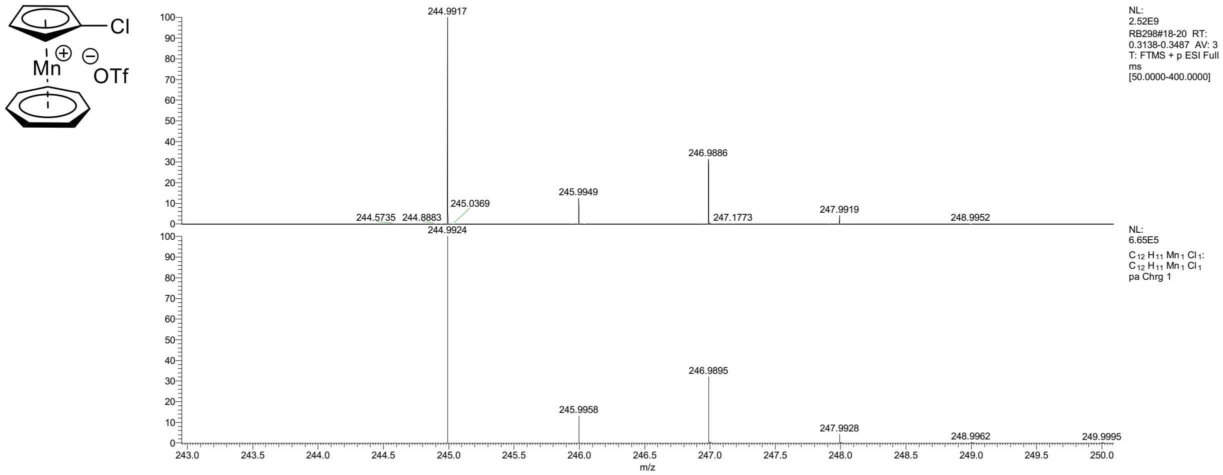


Figure S56. MS (ESI pos, [m/z]; *top*: experimental, *bottom*: simulated) of **12**.

## 2. Cyclic Voltammetry

### 8- Tromanceniumyltrifluoridoborate (7)

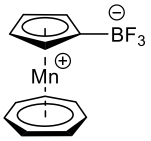


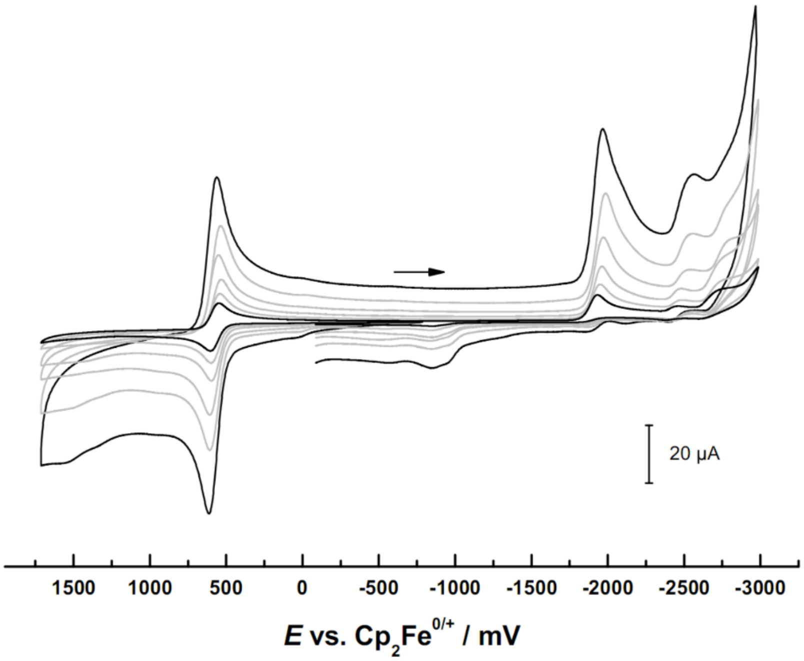


Figure S57. Cyclic voltammogram of **7** in acetonitrile at scan rates of 25 $mVs^{-1}$ to 1000 $mVs^{-1}$.

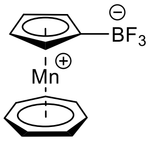


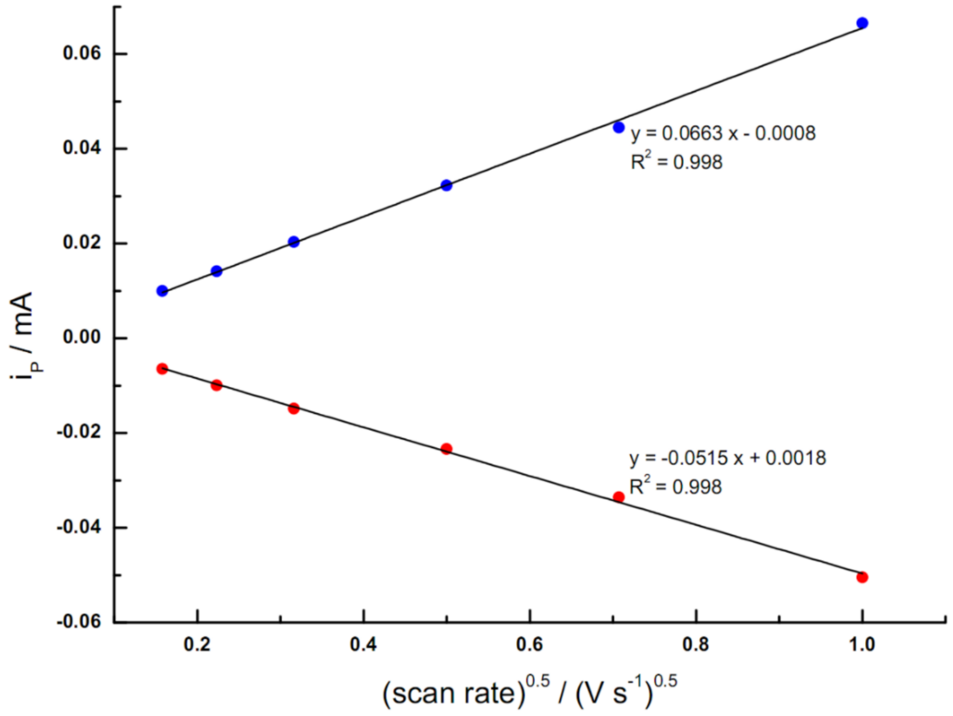


Figure S58. Reversibility plot for the first oxidation of **7**.

**8-Tromanceniumylboronic acid pinacol ester triflate (8)**

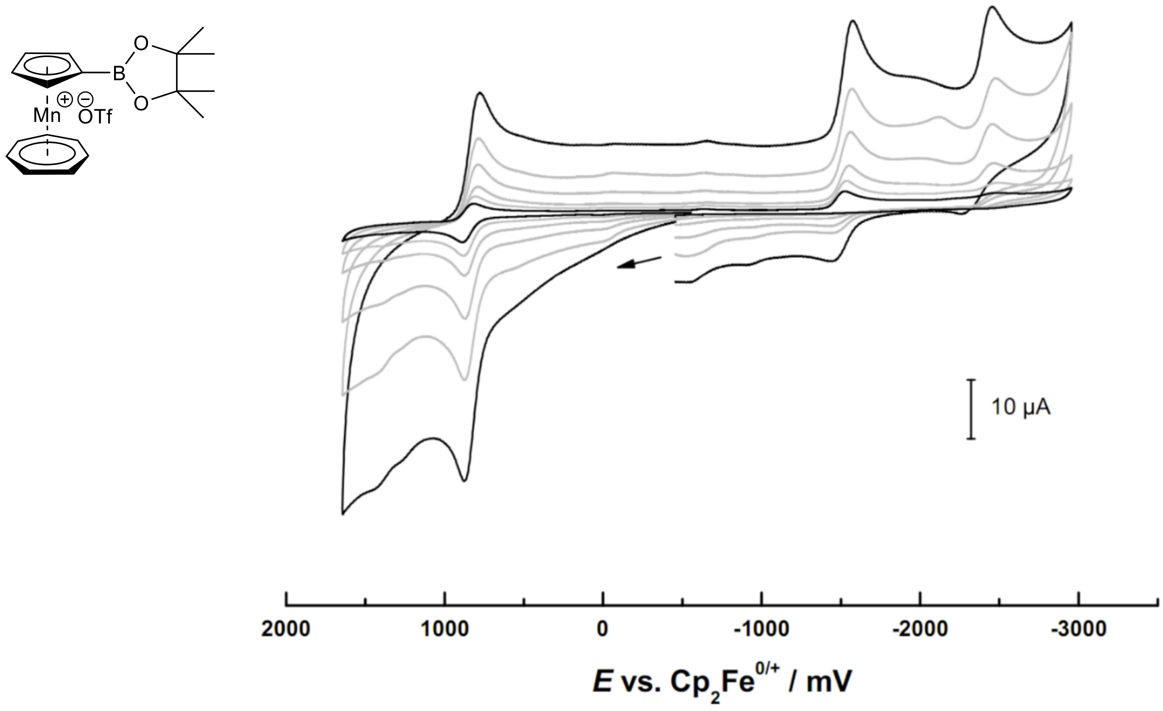


Figure S59. Cyclic Voltammogram of **8** recorded in acetonitrile at scan rates from 25 mVs$^{-1}$ to 1000 mVs$^{-1}$.

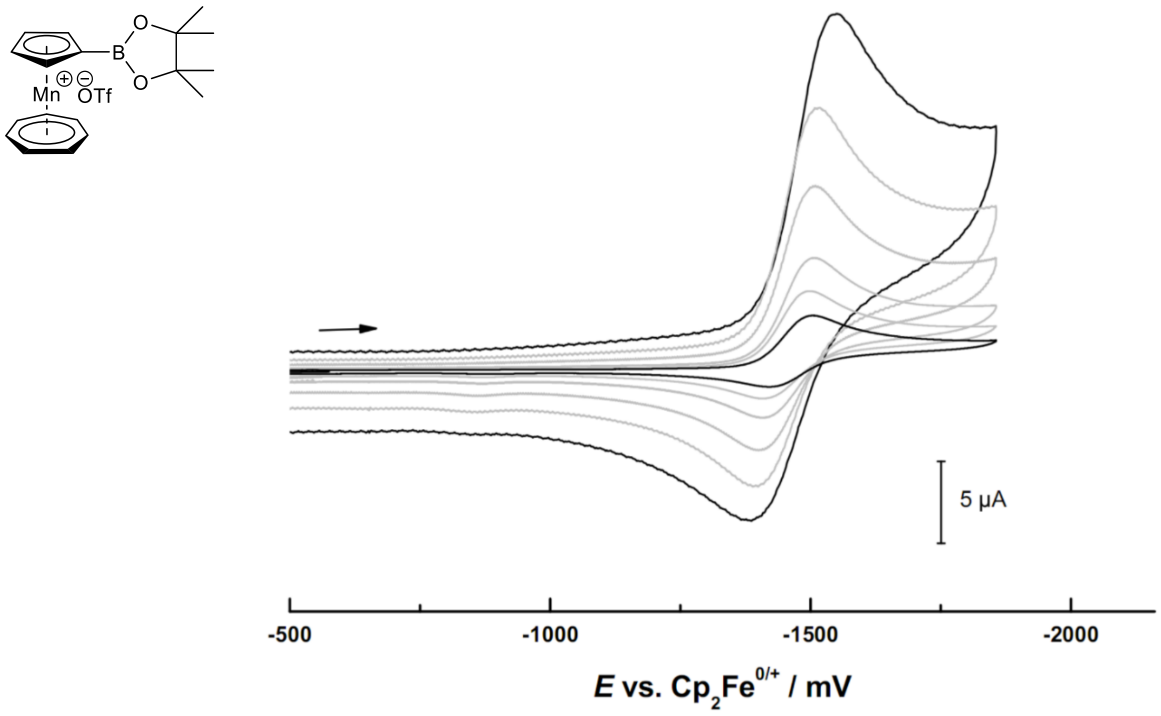


Figure S60. Full scan of the first irreversible reduction of **8** at scan rates from 25 mVs$^{-1}$ to 1000 mVs$^{-1}$.

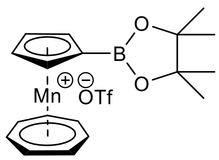


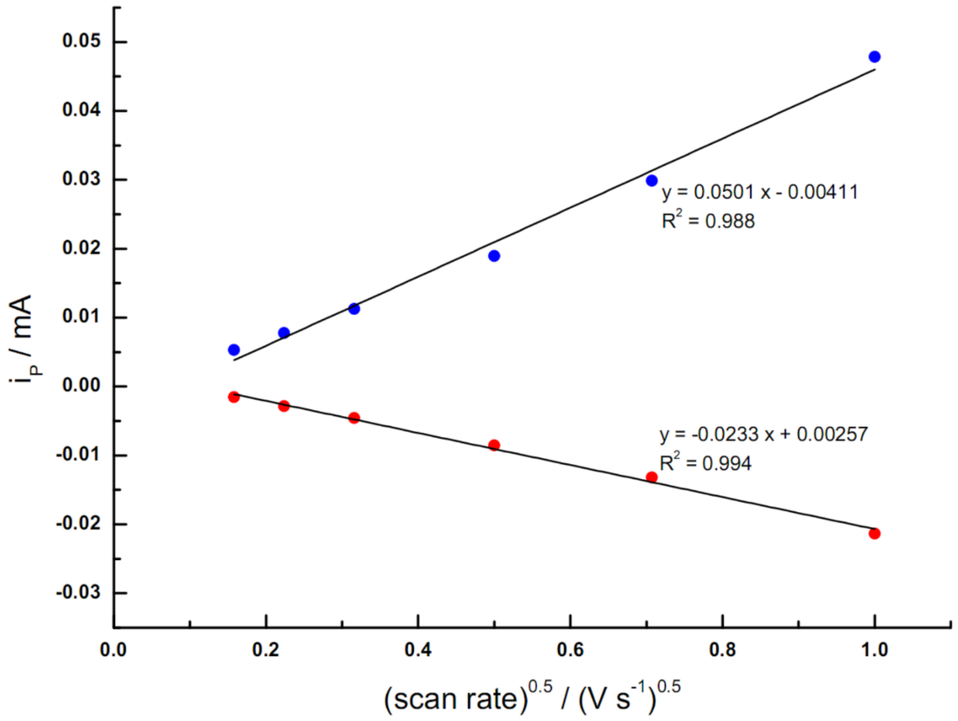


Figure S61. Reversibility plot of the first quasi-reversible oxidation of **8**.

**8-Tromanceniumyl boronic acid triflate (9)**

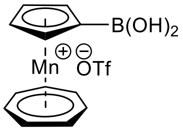


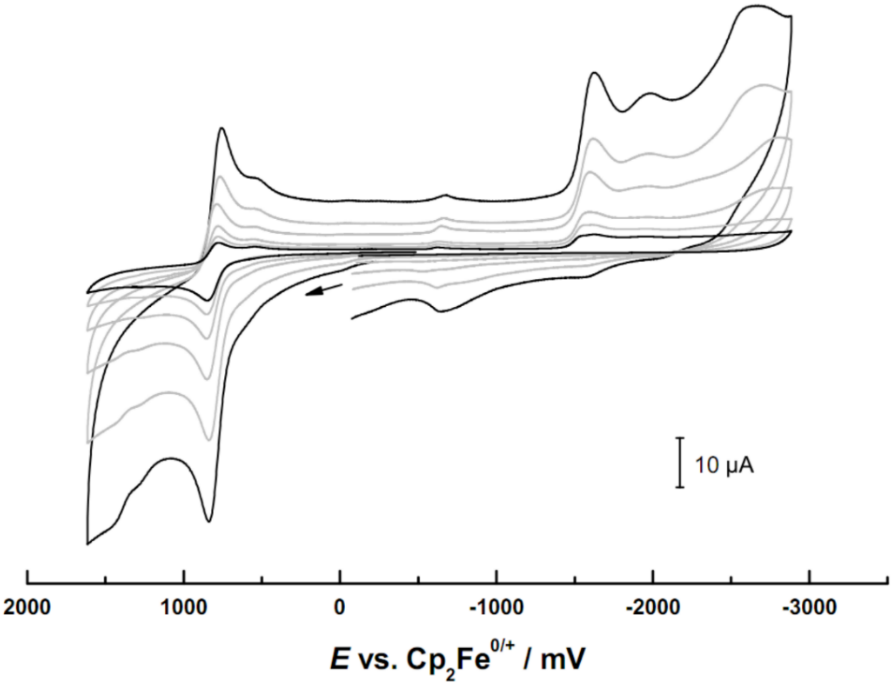


Figure S62. Cyclic voltammogram of **9** in acetonitrile at varying scan rates from 25 $mVs^{-1}$ to 1000 $mVs^{-1}$.

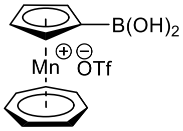


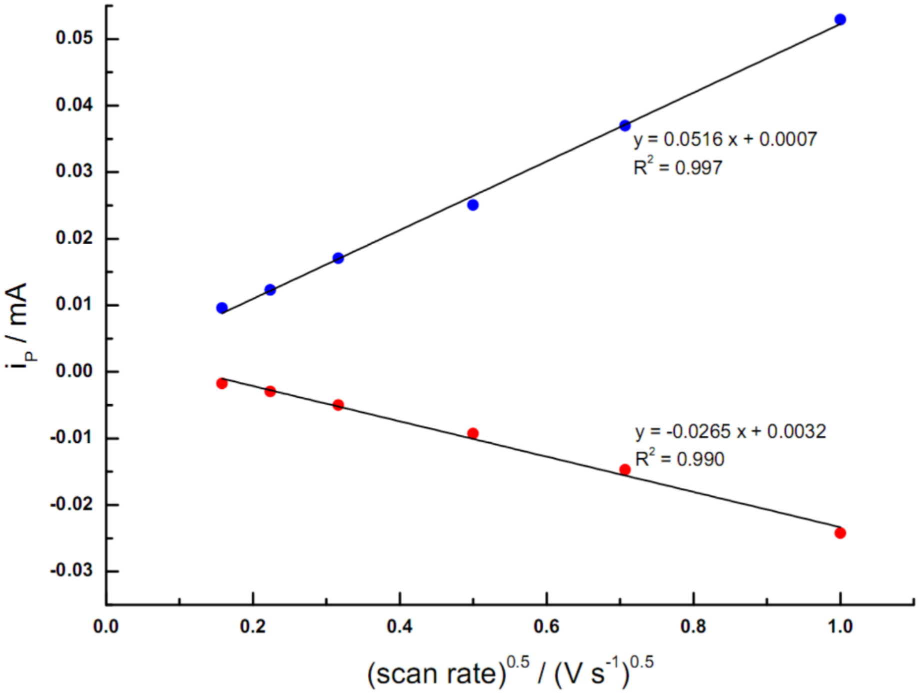


Figure S63. Reversibility plot for the first quasi-reversible oxidation of **9**.

**8-Chlorotromancenium triflate (12)**

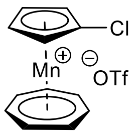


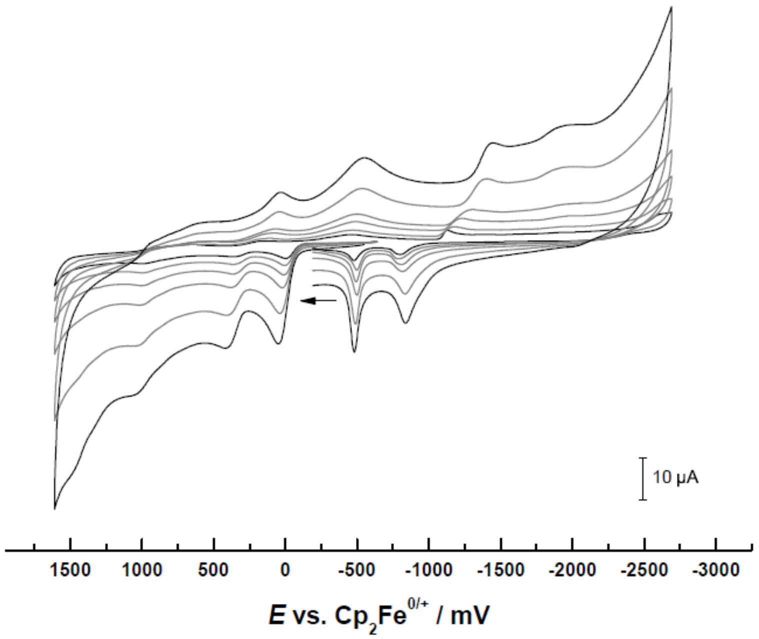


Figure S64. Cyclic voltammogram of **12** recorded in acetonitrile. Scan rate vary from 25 $mVs^{-1}$ to 1000 $mVs^{-1}$.
